# Before Azaria: A Historical Perspective on Dingo Attacks

**DOI:** 10.3390/ani12121592

**Published:** 2022-06-20

**Authors:** Adam Brumm

**Affiliations:** Australian Research Centre for Human Evolution, Griffith University, Nathan Campus, Brisbane, Queensland 4111, Australia; a.brumm@griffith.edu.au

**Keywords:** dingo attacks, past human–dingo interactions, Azaria Chamberlain, Australian cultural history

## Abstract

**Simple Summary:**

When nine-week-old Azaria Chamberlain disappeared from a central Australian campsite in 1980, few accepted her mother’s claim that ‘A dingo’s got my baby’. Dingoes, it was popularly believed at this time, simply did not attack humans. A recent spate of dingo attacks has repudiated this widespread narrative—but when and why did it arise? Analysis of historical Australian print media shows that, in fact, dozens of accounts of dingo attacks were published between 1804 and 1928. It is difficult to separate the empirical events from the cultural milieu in which they were reported, but some of these historical accounts are credible, resembling those of modern attacks. It is also evident that up until the early 20th century it was a commonly held perception that dingoes did occasionally prey on humans. By the 1920s, however, a popular belief had taken hold that dingoes were far too timid to attack even children and had never represented a threat to human safety. This cultural shift in the image of the dingo may be traced to the reduced frequency of human–dingo interactions in the more settled regions of eastern Australia, where a veritable war of destruction waged by pastoralists nearly eradicated the population. Intensive shooting, trapping and poisoning may also have selected for dingoes that were more wary of humans, such that the changing public attitude towards the dingo reflected the reality of rural life.

**Abstract:**

This paper investigates the origin of the once popular belief in Australian society that wild dingoes do not attack humans. To address this problem, a digital repository of archived newspaper articles and other published texts written between 1788 and 1979 were searched for references to dingoes attacking non-Indigenous people. A total of 52 accounts spanning the period between 1804 and 1928 was identified. A comparison of these historical accounts with the details of modern dingo attacks suggests that at least some of the former are credible. The paper also examined commonly held attitudes towards dingoes in past Australian society based on historical print media articles and other records. Early chroniclers of Australian rural life and culture maintained that dingoes occasionally killed and ate humans out of a predatory motivation. By the early decades of the 20th century, however, an opposing view of this species had emerged: namely, that dingoes were timid animals that continued to pose a danger to livestock, but never to people. This change in the cultural image of dingoes can possibly be linked to more than a century of lethal dingo control efforts greatly reducing the frequency of human–dingo interactions in the most populous parts of the country. This intensive culling may also have expunged the wild genetic pool of dingoes that exhibited bold behaviour around people and/or created a dingo population that was largely wary of humans.

## 1. Introduction

**Figure 1:**
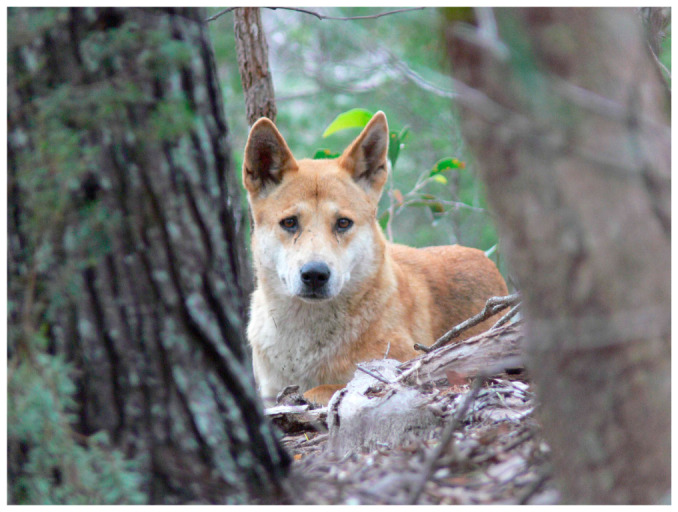
A wild dingo (*C. dingo*). This photograph, taken in 2007 on K’gari (Fraser Island), is of a five- to six-year-old male. Credit: Rob Appleby.

The dingo (*Canis dingo*) is a medium-sized (15–16 kg) free-ranging canid (Figure 1) that is ‘native’ to Australia [1], having been an integral part of the mainland fauna for at least 3200 years [2]. Dingoes appear to have been introduced to northern Australia from a source in island Southeast Asia, probably arriving initially on these shores as the domesticated dogs (*C. familiaris*) of seafaring hunter-gatherers [3]. If the latter supposition is correct, then it seems possible that the antecedent dingo population (or populations) underwent a process of feralisation in Australia, eventually reverting to a wild ancestral state [4]. Much about the dingo’s history remains uncertain, however [4,5].

This paper examines the origin of the formerly widespread narrative in Australian society that the wild dingo does not attack humans. This once popular notion came to the fore in the early 1980s with the disappearance of baby Azaria Chamberlain and the subsequent wrongful conviction of her mother, Lindy, for her murder [6,7,8,9,10,11,12,13]. The Chamberlain affair was a key event in the modern cultural story of Australia [14,15], one in which public debate about the behaviour of free-ranging dingoes was central (see Appendix A). Lindy Chamberlain’s claim that a dingo took her infant child from the family’s tent at Uluru in central Australia in August 1980 was greeted with widespread incredulity [6,9]. Most Australians at this time held to the belief that the dingo is a timid animal that had never been known to prey on humans, or attack or harm them physically in any way them without provocation, at any time in Australia’s history [6]. The media hysteria surrounding the Chamberlain case [14,15], and the flawed forensic evidence presented by the public prosecutor (the ‘Crown’), were key factors in this egregious legal miscarriage [16], but so too was this entrenched cultural attitude towards dingoes [6,9]. As one contemporary observer pointed out: ‘The prosecutors’ case [in the Chamberlain murder trial] began with this premise: dingo attack is an implausible excuse for the disappearance of a baby, an unprecedented suggestion, unworthy of belief’ [6] (p. 281) (see also Appendix A).

It is now evident that this belief that dingoes do not attack humans without provocation was wrong: indeed, since the 1990s there have been many documented incidences of dingo attacks on K’gari (Fraser Island) [17,18], including the death of nine-year-old Clinton Gage in 2001 (Appendix B). The current picture of the dingo is of an intelligent and powerful hunter, but also a highly versatile scavenger that readily exploits anthropogenic foods when available. The latter behaviour brings some dingoes into close contact with people, and, in very rare cases, for reasons that are not yet fully understood, attacks have occurred [17,18]. The number of serious dingo attacks in modern Australia is very small compared with those attributed to sharks—and even they occur relatively infrequently [19]—but they have taken place on a few occasions, with tragic results [17,18]. It is thus worth examining why the concept that dingoes were not a danger to people was so common until recent decades, and when and how this narrative arose.

To address this problem the paper considers two interrelated issues: (1) if there is any plausible evidence from the earlier history of Australia for wild-living dingoes preying on humans (i.e., attacking and/or killing and eating them); and (2) if the idea was given credence at these times that the wild dingo has the propensity to do physical harm to humans in the form of an unprovoked predatory attack. Much the same issues were raised during the court proceedings and public hearings related to the disappearance of Azaria Chamberlain [6,9]. For instance, in the first inquest (1981) the coroner directly asked the public for reports of dingoes attacking children, citing as evidence ‘the disappearance of a two-year-old child from a veranda near Gympie about a hundred years ago’ [6] (p. 224)—this referred to the 1880 case of Willie Gesch (see Appendix C). Furthermore, as indicated in Appendix A, the Chamberlains received various letters from members of the public about historical attacks, including old stories about children being taken by dingoes. In preparing their defence the Chamberlains probed deeper into some of these accounts, but, as Lindy Chamberlain later recounted in her autobiography, their results were not taken seriously:

When we tried to document some of these incidents for court, we were told they were too early to be listed in court records, considered irrelevant, or misleading (since they were usually listed as death by ‘misadventure’), or just reported in local newspapers of the day

[9] (p. 91)

It is evident that this issue of whether dingoes ever preyed on humans in the earlier history of Australia has never been adequately addressed. This leaves a gap not just in our understanding of the Chamberlain affair, a landmark event in the Australian story [14,15], but in the historical development of cultural attitudes towards this icon of the mainland fauna. This study may also have implications for our knowledge of dingo behaviour and for current dingo management strategies. The present consensus is that dingo attacks are a distinctly modern phenomenon linked to the changing behaviour of dingoes in a few areas where, over recent decades, increased human presence has led to the habituation of these canids (especially at popular camping grounds on K’gari) [17,18]. Hence, if there is credible evidence for dingo attacks prior to these modern human-induced changes, then there may be shortcomings in current scientific knowledge about dingo behaviour and the causes of negative human–dingo interactions in the present.

In the first part of this study, a number of historical accounts of dingo attacks are identified that are held to have taken place many decades before the disappearance of Azaria Chamberlain. The inherent problems in the nature of these historical accounts, their veracity, and degree of similarity (or not) to modern dingo attacks as documented, are considered. In the second part of the study, it is argued that there was, in fact, at one stage, a longstanding belief in Australian society that wild dingoes do sometimes attack and kill humans without provocation—that is, out of a predatory motivation. It is contended that this belief had roots in the earliest colonial period and that it persisted over many generations. It is further evident that by a particular point in Australia’s modern cultural history this notion had been replaced by an opposing image of dingoes—that of benign creatures that never directly harm humans. This cultural shift in the perception of dingoes and their propensity to attack humans sheds new light on the development of the Chamberlain-era attitude towards these wild canids. The study also considers how the colonial-era belief that dingoes posed a danger to humans arose, and why this narrative was abandoned prior to the 1980s. Both questions are relevant for our understanding of the wild dingo world and the relationship between humans and dingoes today.

The research is focused on the history of non-Indigenous Australians, but another important issue to consider is whether there is any evidence for wild dingoes attacking Aboriginal people prior to the British irruption in 1788, or in the post-contact period—questions that were also raised during the Chamberlain trial and subsequent proceedings [6]. This is beyond the remit of the current study. Historical accounts suggest that during the period of early European settlement Indigenous peoples in Australia had a complex relationship with dingoes that was based on taking pre-weaned pups from wild dens and hand-rearing them for temporary adoption as ‘pets’ [5,20,21]. A separate study is required to examine this human-canid relationship in detail, such that any evidence for dingoes attacking Aboriginal people can be fully contextualised.

## 2. Materials and Methods

The study consists of two parts. First, a list is compiled of historical accounts of dingo attacks in Australia prior to the early 1980s. The information in these reports is then assessed to determine whether it is plausible or not. Second, the study investigates cultural attitudes towards dingoes and the issue of human safety over the span of the same time period (i.e., pre-1980, post-1788). The outcomes of these two strands of research are presented separately in Results.

### 2.1. Compiling a List of Historical Accounts of Dingo Attacks

The aim was to identify early written accounts of unprovoked attacks by wild dingoes against humans, resulting in human–dingo contact, injury or death. A search was also conducted for reports of incidents in which wild-living dingoes were suspected by authorities or other contemporary observers of being involved in the deaths or disappearances of people. Certain types of dingo attacks described in the historical literature were excluded. For example, the British marine officer Watkin Tench (1758–1833) claimed that Aboriginal people in the vicinity of the early Sydney colony would sometimes ‘command’ their dingoes to harass or attack colonists if they encountered them alone outside the settlement [22]. As this claim apparently involved human-socialised dingoes (i.e., companion animals) it was excluded from the study. Cases in which people were attacked by captive dingoes or those kept as pets were also not included. Finally, the study omitted reports of dingo bounty hunters (‘doggers’) or shooters being injured by trapped or wounded dingoes (e.g., [23]), which would fall within the realm of provoked/defensive attacks. For the same reason the account of the Chamberlain trial juror’s encounter with a wild dingo was excluded (see Appendix A).

To look for accounts of past dingo attacks, a review was conducted of the published literature and a search was undertaken of Trove, a publicly-accessible online database of digitised Australian print media (for similar approaches, see [24,25,26,27]). The primary Trove search terms used were: ‘dingo’, ‘native dog’, ‘wild dog’, ‘dingo/native dog/wild dog attack’, ‘dingo/native dog/wild dog child/infant’, and ‘dingo/native dog/wild dog devours’. Other common words for the dingo used in the past (e.g., warrigal) were also employed. The database search involved manually trawling through a large amount of irrelevant material (e.g., ‘native dog devours’ yields ~9000 total results). No written code was used, including to filter out the OCR (optical character recognition) errors common to Trove.

The Trove search introduced inherent limitations to the study. Many major and provincial newspapers and gazettes published in Australia up until the late-1950s are available through Trove, along with other print media (e.g., magazines and newsletters), though not all. There is also a temporal gap in the Trove database from the late 1950s onwards (the *Canberra Times* is the only major newspaper available in digitised form after 1957). Moreover, the earliest period of colonial settlement (1788 until the turn of the century) is not well represented because the colony’s first newspaper, the *Sydney Gazette*, was only established in March 1803 [28]. This was the only newspaper produced in mainland Australia up until the mid-1820s, when other Sydney papers, and the first provincial newspapers, were founded [28,29,30]. Trove coverage of the *Sydney Gazette* is complete (although publication of the paper was suspended between August 1807 and May 1808 [28]). It should also be noted that until the mid-19th century the mass reading public received much of its news from the cheaply printed leaflets known as broadsides (or broadsheets), few of which have survived [29,31]. This is not a concern, however, as the broadside printers borrowed heavily from the content of contemporary newspapers [31], including those represented in Trove (e.g., *Sydney Gazette* and others).

The study endeavoured to bridge these temporal gaps through a literature search of texts published during the missing time periods, although the years 1788 to 1803 remain a blank spot. Web searches were also conducted and leading specialists in dingo behaviour consulted. Where possible, newspaper articles were cross-checked by examining archived records (e.g., police reports) and through liaising with local historical societies. A more comprehensive study would involve a search for reports of dingo attacks in early memoirs, unpublished diaries, letters and other archival materials in all mainland states and territories, but this was beyond the scope of the paper. It is contended that the Trove database provides sufficient insight into accounts of dingo attacks available in the early Australian print media, although it is unlikely this study would have identified them all (e.g., owing to OCR errors).

### 2.2. Investigating Earlier Cultural Attitudes towards Dingoes

The objective of this part of the study was to glean insight into what earlier generations of people living in this country have expressed in the written record about the propensity of dingoes to attack humans. Prior to 1980 had Australians always regarded dingoes as posing no direct danger to people? Or did colonial settlers, for example, those who lived in Australia between 1788 and the federation of the colonies in 1901, believe that it was possible for a human to become the prey of dingoes? If so, why, and at what point in time did attitudes towards these canids change, and for what reason? To address these questions, the paper examined early print media for discussions and commonly held perceptions about dingoes and their interactions with humans. The focus again was on Trove searches. The use of Trove faced the same limitations identified above, but it is contended that this approach was adequate for gaining an understanding of cultural attitudes towards dingoes over a long period of time in Australia. The results have not been tabulated, instead being presented within a critical narrative framework.

## 3. Results: Historical Accounts of Dingo Attacks

**Figure 2:**
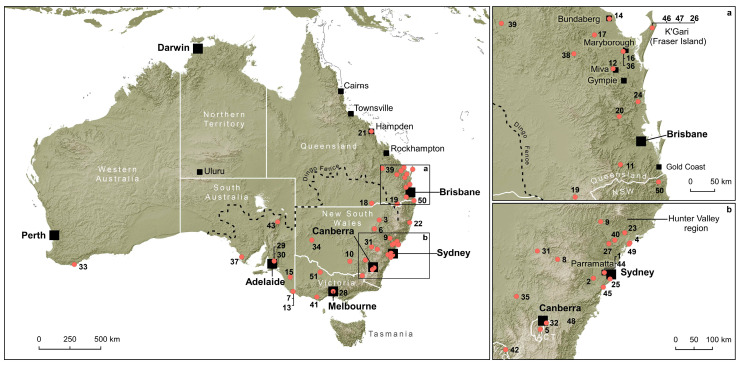
Map showing the distribution of historical accounts of wild dingo attacks. The locations of the historical dingo attacks are represented by the enumerated red dots. The broken line denotes the dingo barrier fence (‘Dingo Fence’), established in the mid-20th century to keep dingoes out of the rich sheep country of south-eastern Australia. Key: 1: Prospect; 2: Nepean district; 3: Namoi River; 4: Hunter River district; 5: Mount Tennant, Tharwa; 6: Coonabarabran; 7: Mount Gambier; 8: Bathurst district; 9: Muswellbrook; 10: Thompson’s Swamp; 11: Fassifern; 12: Munna Creek; 13: Mount Gambier; 14: Bundaberg area; 15: Carew; 16: Maryborough; 17: Booyal; 18: Daymar; 19: Pikedale; 20: Kilcoy; 21: Hampden; 22: North coast district; 23: Paterson; 24: Nambour; 25: Botany Bay; 26: K’gari; 27: Wollombi; 28: Melbourne; 29–30: Para River; 31: Molong Creek; 32: Queanbeyan; 33: Oyster Harbour; 34: Menindee; 35: Murrumburrah; 36: Maryborough; 37: Mount Drummond; 38: Gayndah; 39: Dawson River; 40: Saddler’s Creek; 41: Carpendeit; 42: Upper Murray district; 43: Gammon Ranges; 44: Parramatta; 45: Clifton; 46–47: K’gari; 48: Braidwood; 49: Merewether; 50: Murwillumbah; 51: Budgerum. The location of one historical account of a dingo attack, from ~1889 (see Table A1), is not represented, as the only geographical information available was ‘Queensland’. It should be noted that there have never been dingoes in Tasmania, a continental island that was sundered from mainland Australia by post-glacial sea level rises. Map credit: Kimberlee Newman.

**Figure 3:**
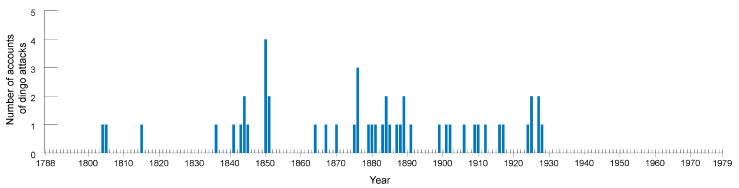
Distribution of historical accounts of dingo attacks (*n* = 52) in mainland Australia by year. There is a temporal gap in the coverage between 1788 and 1802, as Australia’s first newspaper (the *Sydney Gazette*) was not established until March 1803. The *Sydney Gazette* was the only newspaper published in Australia until the mid-1820s, although it was not published between August 1807 and May 1808. No accounts of dingo attacks were identified in the 50 years between 1929 and 1979. Azaria Chamberlain was killed by a dingo at Uluru, central Australia, in August 1980.

The search yielded a total of 52 historical accounts of dingo attacks in mainland Australia (Table A1; Figure 2). The earliest account is from 1804 and the most recent dates to 1928 (Figure 3). No historical reports of dingo attacks were found from the period between 1929 and 1979. Similarly, a recent study [32] of media reports of dingo attacks against humans between 1940 and 2019 did not uncover any evidence for such incidents taking place (or at least being reported in digitised newspapers available through Trove) between 1950 and 1979. Only four accounts from the period between 1940 and 1949 were identified in that prior work [32], but these were all excluded from the present study for the reasons outlined above (i.e., in each instance the dingoes seem to have been provoked or goaded in some way).

The majority of the accounts (58.3%) found in this study are from the period between 1841 and 1889 (*n* = 48; dates are not available for four) (Table A1). Most are from New South Wales (*n* = 21) and Queensland (*n* = 17) (Figure 2). There were also seven accounts from South Australia, four from Victoria, two from the Australian Capital Territory, and one from Western Australia. Most claims of dingo attacks in New South Wales are from the period prior to 1876 and relate to incidents that occurred in Greater Sydney and the Hunter regions, the former the most densely populated and closely settled part of the state. All of the Queensland accounts, with one exception, date to after 1876 and are from the south-eastern corner of the state, where European settlement was historically concentrated. The northernmost account is from Hampden, about 20 km west of Mackay in Queensland’s central coast. No accounts were found from the arid zones of central and western Queensland or in the northern tropics. Australia-wide, attacks were almost evenly split between children (<18 years of age) (*n* = 26) and adults (*n* = 25). One account of an attack involved both (Table A1).

All of these early claims of dingo attacks need to be treated with caution. The data are fragmentary, of variable quality, anecdotal in nature, and, in some cases, as described in Appendix D, contradictory. In most instances a brief newspaper report with very little detail is all that is available for analysis. These are clearly not precise records of events (any more than modern media stories about dingo attacks are [32]). Moreover, the nature of the historical accounts is such that they cannot presently be verified by independent lines of inquiry, despite the effort to do so (see Table A1). There is a possibility that contemporary reports of dingo attacks were exaggerated by the persons involved or embellished by the anonymous newspaper reporters, a not uncommon practice at the time [29] (and one that continues in some modern reportage of dingo attacks [32]). From the fragmentary evidence available it is also not possible to be certain whether the incidents described involved unprovoked acts of dingo predation or if the animals had been harassed or goaded by humans, whether knowingly or not. Concerning the latter, for example, Thomas Davis (1828–1904), father of Australian author Steele Rudd, described in his unpublished memoir a confrontation with dingoes that he had unwittingly provoked while camping with his Aboriginal companion on the Condamine River in the 1850s [33]. The pair had been terrorised all through the night by a large group of yowling dingoes that circled menacingly around their camp [33]. In the morning they discovered that this predicament was due to them having inadvertently established their campfire beside a hollow log containing a litter of newborn dingo pups [33].

The supposed fatalities are especially problematic. As described in Table A1, and Appendix D, such reports commonly involved a lost child or lone adult entering parts of ‘the Bush’ (an Australian colloquial term broadly meaning any tract of scrub, forest, or uncultivated land situated outside urban areas—i.e., the wilderness) that were said to have been ‘infested’ with dingoes, and never being seen alive again. Sometimes the partial skeleton or mangled body parts of the missing persons were found deep inside dingo territory, bearing signs of having been partly eaten by dingoes. In both cases it was inferred or implied that the deceased was killed by dingoes (although not all contemporary observers agreed, and alternative theories were sometimes considered). It would seem, however, that there were never any witnesses (but see the 1891 Budgerum case (Table A1, Appendix D)). It is therefore unclear if dingoes were responsible for the deaths of these people or if they died of another cause and dingoes simply scavenged their remains (in some instances this may be the most likely explanation). Another confounding factor is that, in the case of each supposed fatality, there seems to have been no autopsy or formal investigation (inquest) conducted at the time to establish the manner of death. This should hardly be a surprise, in light of the fact that major questions can be raised about the completeness and accuracy of recording of cause of death throughout much of the colonial history of Australia [34].

The veracity of the historical accounts of fatal dingo attacks against children is particularly difficult to assess owing to the lack of detail. Most appear only as short notes in provincial newspapers. For example, a report of a dingo seizing and carrying off a child near Bundaberg in 1885 merited just 34 words in the local newspaper [35] (Table A1). The brevity of these accounts is striking, given that an outstanding characteristic of the colonial print media was the excessive length of stories about issues that seem droll or irrelevant by today’s standards [28,29].

Another factor to consider is the genetic ancestry of the dingoes in the historical accounts. There is a longstanding belief in Australia that interbreeding between dingoes and domestic dogs results in hybrids that manifest some morphological features and behavioural traits that are not found in genetically ‘pure’ dingoes [1] (see also Appendix E.2). It is impossible to know whether the historical accounts of dingo attacks identified in this study involved ‘pure’ dingoes, dingoes with some domestic dog ancestry, or both. In one sense it is a moot point, however, given that almost any dingo encountered after European settlement (except in the most remote areas) could potentially have derived from a lineage that had undergone genetic admixture with European domestic dogs [36]. Even the K’gari dingoes—supposedly among Australia’s ‘purest’ from a genetic perspective—may have at least some dog DNA [37], as domestic canines were only banned from the island in 1981 [38]. There might also have been introgression between dingoes and dogs in the vicinity of Uluru prior to Azaria Chamberlain’s disappearance (see, e.g., [39]), raising the possibility that the dingo that took her was not a ‘pure’ dingo. It is at least possible to exclude the prospect that the dingoes in question were rabid (Australia is historically free of canine rabies [40]), an issue that complicates efforts to distinguish between cases of predatory and non-predatory wolf (*C. lupus*) attacks in Europe and North America [41].

In sum, there are major limitations in how this historical material can be used. A reasonable case can be made that all of these early accounts of dingo attacks should be dismissed outright, given the questionable context and inconsistencies that are characteristic of some (see Appendix D). On the other hand, it seems evident that the standard of record-keeping during the historical period in question (especially the early years of settlement) was so uneven and of such generally low quality (Appendix D) that we cannot say with certainty that dingo attacks, if they did occur, would have been reliably documented—or reported at all. Moreover, as will now be contended, patterns are evident within these historical accounts of dingo attacks, and there are also a number of noteworthy parallels with the various dingo attacks that have been documented in modern times, suggesting there might be some truth behind the former.

### 3.1. Comparison with Modern Dingo Attacks

It is not easy to make direct one-to-one comparisons between the historical accounts of dingo attacks and those recorded in recent years. This is not just because the former are generally so lacking in detail, as previously noted. Indeed, modern eyewitness accounts of dingo attacks on K’gari might also be unreliable: witnesses, despite their best efforts, may give unclear or conflicting versions of events, and some statements could also contain questionable information—possibly to conceal the fact that victims had been hand-feeding or otherwise interacting closely with the dingo prior to the attack, which are prohibited activities (R. Appleby, pers. comm. 2022). It is also the case that specialists in human–dingo conflict on K’gari still do not fully understand what had motivated individual dingoes to bite, harass, or otherwise harm people in many instances, so it is difficult enough to make meaningful comparisons across even these relatively well-documented modern cases [17]. At best, broad comparisons can be made between the historical accounts and the modern incidents in order to identify apparent similarities and points of difference.

To begin with, historical accounts of lone adults being attacked in the Bush by wild dingoes while intoxicated or otherwise incapacitated (Table A1) are strikingly similar to an incident that took place recently on K’gari. In July 2012, a 23-year-old male backpacker (identified only as ‘Justin’) wandered off from a K’gari campsite at around 2:30 am, reportedly while inebriated, got lost in the Bush, and then fell asleep or blacked-out [42]. According to one account:

Some time later he was startled awake by dingoes sinking their teeth into his limbs. He tried to fight them off, hitting out at them as they bit him. However, if he scared one off, another ran in, snapping, biting and taking turns to clamp down on his arms and legs

[43] (p. 98)

The man claimed in a subsequent media interview that he managed to run away and climb up a tree, but the branch he hoisted himself onto broke and he fell to the ground, whereupon he picked up a stick to defend himself [43]. At some point the dingoes ceased their attack, and he was able to make his way back to the campsite at dawn [43]. A newspaper report stated that he ‘suffered extensive injuries to his arms, legs, and head’ [43]. It is also evident from this recent case of a dingo attack on K’gari that the historical accounts of people being ‘treed’ by dingoes (described in Appendix D and Appendix E and Table A1) should not necessarily be dismissed as exaggerations or fabrications.

Other modern incidents indicate that some dingoes will opportunistically attack adults when they have the advantage of numbers—a recurring feature of the historical accounts (Appendix D and Table A1; and see below). For example, in August 2014 a K’gari resort chef, 25-year-old Dane Allan, was set upon by four dingoes when he went outside the resort fencing late at night and walked to the beach alone [44]. According to the attending ambulance officer:

He received bites and skin tears and the dogs tore at his shorts. He ended up on the ground and they continued to bite him on the legs and the head, but he tucked himself up into a ball with his knees into chest to try and protect himself and he managed to protect his throat and his stomach and groin

[44]

There have been various other recent dingo attacks of this nature [12,17,18,43]. Even in the late 1990s, K’gari residents were claiming that ‘many adults have been attacked while walking alone on the Fraser Island beach’ [45] (p. 6).

These modern attacks on adults are broadly consistent with some of the historical accounts of attacks involving adults, but these resemblances are not limited to adults. Historical accounts in which children have supposedly been seized and borne away by dingoes (e.g., Willie Gesch (Appendix C)) are also reminiscent of modern incidents. For example, during the 1987 royal commission into the Chamberlain convictions evidence was given that on 23 June 1980 a three-year-old girl who was visiting Uluru National Park with her family had been ‘seized around the head and neck by a dingo and, apparently, dragged out of a car and some little distance along the ground before her father confronted the dingo’ [46] (p. 281). There have also been several recent cases of free-ranging dingoes seizing and dragging away toddlers and small children at camping grounds and other tourist areas on K’gari (see Appendix B for details). In each case a parent or other adult managed to rescue the youngster. Given the facts of these and other recent cases, it is not unfeasible to infer—though impossible to prove—that at least some accounts of small children becoming victims of dingo predation in the earlier history of Australia (Table A1) could have been genuine.

### 3.2. Seasonality of Attacks

It is also important to consider what time of year the historical dingo attacks were supposed to have taken place. Dingoes have one annual oestrus cycle, with the breeding season typically commencing around March and finishing in May [1,47,48,49,50,51,52]. The dingo whelping season, when litters are born, is in winter (June–August), pups are weaned in the spring (September–November), and juvenile dingoes are trained to hunt and survive in the summer (December–February) [50]. Subadult male dingoes typically become more aggressive during the breeding season, and during the whelping season dingo parents and alloparental carers are under continual stress to feed litters of newborn pups [18].

Importantly, a recent analysis of negative human–dingo interactions on K’gari between 2001 and 2015 (*n* = 160), ranging from stalking and chasing to serious attacks, found ‘a consistent pattern of incidents peaking in March/April and also July, corresponding with dingo breeding and whelping seasons (respectively)’ [18] (p. 146). In fact, nearly half (44%) of the 160 records occurred in March (13%), April (18%) and July (13%). Notably, Clinton Gage was killed in the breeding season (Appendix B). Furthermore, the 1997 attack against Andrew Bartram took place during this same time of year (March), as did the 1998 attack against Kasey Rowles (April), the 2019 case involving Hunter Allister (April), and two of the 2021 Orchid Beach incidents (April–May) (Appendix B). (Azaria Chamberlain was killed in the whelping season [August]). Some of these peaks coincide with school holiday periods when many families visit K’gari, typically for recreational camping [18]. It is generally accepted, however, that dingo attacks against humans (and other forms of negative human–dingo interactions and threatening behaviour) exhibit a strong seasonal trend that is most likely linked to natural changes in dingo behaviour at these particular times of year [18].

**Figure 4:**
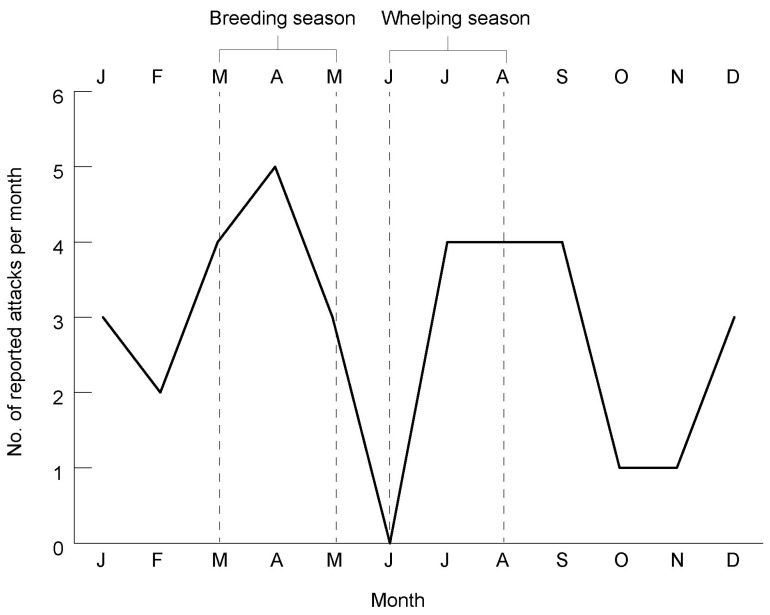
Seasonality of dingo attacks in historical accounts (*n* = 34) for which the month of the incident is known. Data source: Table A1. Where a date range for an historical account was given (e.g., April to May, late April/early May), the first month was used (i.e., April).

It is therefore noteworthy that, of the 34 historical accounts identified here involving reputed attacks against humans (adults and children) in which the month of the incident is known (Table A1), over one-third (35.3%) occurred during the March–May breeding season (Figure 4). Moreover, well over half (61.8%) took place in March–April (26.5%) and July–September (35.3%), corresponding to the breeding season and the whelping season, respectively (the whelping season may also extend into September [1]). The seasonal patterning evident in these data helps to build confidence in the notion that there is some veracity behind the historical accounts of dingo attacks. It is also worth noting that in the case of the alleged attack against 10-year-old George Emery in July 1901 the dingo was tracked to a denning site with young pups present (Table A1). Moreover, in the case of the disappearance of four-year-old Harold Halliday in August 1912 the Aboriginal tracker encountered batches of young dingo pups in the search area (Table A1). 

### 3.3. Habituation and Food-Conditioning as a ‘Modern’ Phenomenon

The reports of dingo attacks in historical times seem to differ from modern attacks in one notable respect: most of the incidents from recent decades took place at or near popular family holiday campsites or other busy tourist spots [18]. The dingoes ‘responsible’ had also typically been deliberately or inadvertently fed by visitors, and thus had apparently become habituated to the presence of humans (but see [17,18]). For example, a K’gari ranger described the pair of 22-month-old male and female dingoes that killed Clinton Gage as ‘“very, very habituated, camp dogs”’ [43] (p. 135). In these cases, authorities surmise that the dingoes had lost their natural fear of humans, and consequently had become assertive and predatory towards them [53]. This type of potentially dangerous wild dingo is often held by specialists to differ markedly in behaviour from the wild dingoes that live in areas where they have limited or no contact with humans (including remote parts of K’gari). The latter dingoes, it is argued, are extremely wary of humans, fleeing at the sight or scent of them; indeed, they are rarely ever observed by people, except at a distance [50]. Hence, a common twofold assumption is that: (i) the attacks that caused the deaths or injuries of people do not constitute typical wild dingo behaviour; and (ii) conflict directly involving people is a new phenomenon that has arisen over recent decades as a consequence of the unprecedented growth in tourism in wild dingo habitat [17,53].

This viewpoint, if correct, undermines the veracity of the historical accounts of dingo attacks, which clearly belong to a period of time before the advent of anything resembling the modern tourism industry in Australia. Some historical accounts also describe wild-living dingoes attacking people in what would seem from available descriptions to be wilderness areas located far from human settlements (although clearly not totally removed from anthropogenic activity) (Table A1). Were these dingoes that had never seen humans before? If so, the early written accounts of attacks would seem to be inconsistent with the current notion that purely wild dingoes are afraid of humans and would not willingly approach a person, let alone attack one without provocation.

**Figure 5:**
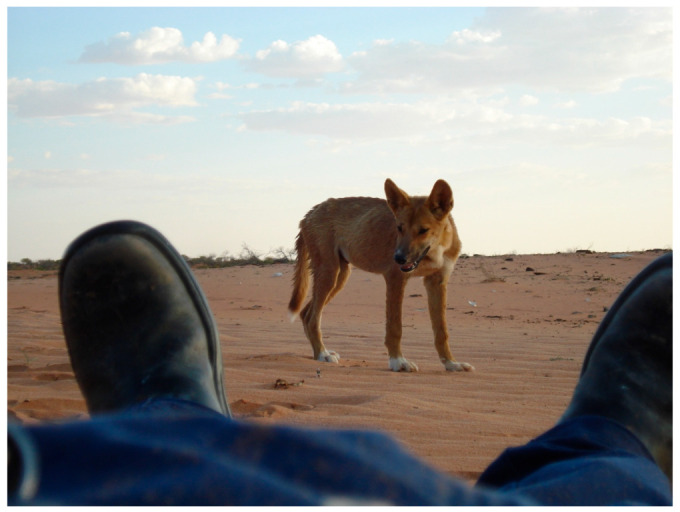
A wild dingo approaching a human seemingly out of curiosity. This behaviour is observed in isolated populations of dingoes that have had no prior interaction with humans. This juvenile dingo was encountered in December 2009 on Quinyambie Station (north-east South Australia) in the Strzelecki Desert. Credit: Benjamin Allen.

Concerning the latter issue, it is important to note that the notion that solely wild-living dingoes are naturally afraid of humans is not strictly accurate. In fact, it is quite common for dingoes that have had no prior interaction with humans to be inquisitive and/or bold around them [17,48,54] (Figure 5). For instance, some dingoes encountered in remote areas will approach people and vehicles out of curiosity and enter isolated campsites at night, usually staying just outside of the firelight, and also sniff at people sleeping in their swag (open canvas bed) (B. Allen, pers. comm. 2022). Early rural dwellers left a number of credible descriptions of this behaviour (e.g., one [55] (p. 160) described a dingo licking his face while he slept and tugging at the saddle he used as a pillow; see also [56]). The key factor is whether or not these dingoes have been persecuted before (e.g., shot at) or even frightened by the presence of humans, such as startled by a fast car, with those that have quickly becoming wary of people (B. Allen, pers. comm. 2022).

There seems to be only two reported cases of this type of unhabituated wild dingo attacking a human in modern times. Both took place in remote parts of the Northern Territory. In May 1997, a man known only as Brian, and his pet dog, were attacked by a group of three or four dingoes around 100 km south of Alice Springs [57]. The man, a Red Cross volunteer, had been walking alone along the side of the highway from Alice Springs to Port Augusta to raise money for charity. It would appear that at the time of the attack he had been sleeping in the open by the side of the road. The dingoes killed his terrier cross puppy, and then turned on him. Using a stick, he managed to fend them off for an hour before a passing truck-driver rescued him. The matter was never reported to police [57]. In another incident that took place on 21 April 2009, Alice Springs nurse Michelle Robson was attacked by a dingo after she was severely injured in a car crash on the Stuart Highway near the Devils Marbles [58]. The driver had left her by a tree while he went for help, and during this time the woman was reportedly attacked and bitten on the hand by a wild dingo [58]. She apparently managed to fight off the animal with a stick [58]. Only sparse details of this case are available (see [59]). Based on these incidents the possibility cannot be discounted that historical reports alluding to similar dingo attacks in the past are valid. These are wild predatory canids: an unhabituated dingo might approach a human out of curiosity (i.e., to investigate something novel in its environment), but if the animal’s hunting motor patterns are triggered for some reason (e.g., the person runs away, or it senses a debility [1]) it could initiate an attack.

Returning to the first issue, it may be unwarranted to assume that the emergence of isolated groups of ‘problem’ dingoes that represent a threat to humans is a recent phenomenon confined to the last few decades. Specifically, it seems reasonable to argue that if habituated and/or food-conditioned dingoes are potentially dangerous today then habituated and/or food-conditioned dingoes would have been potentially dangerous in the early period of our history. It may not be the existence of this behaviour *per se* that is new: rather, the probability of episodes of negative conflict occurring between humans and dangerous dingoes has been heightened owing to the effects of the tourism boom.

This is evident from various reports of dingo attacks on K’gari long before the island became a popular tourist destination (Appendix B). There are also contemporary accounts of the depredations of bold dingoes in and around the early Sydney colony. Indeed, dingoes frequently raided the infant settlement both at night and during the day, killing and eating poultry and other farm animals [60,61]. They also preyed on domestic dogs. For example, the *Sydney Gazette* reported that a dingo brazenly entered a Parramatta house in 1804, located the owner’s pampered lap-dog, which had been put to bed in the oven to keep warm, and ‘with unparalelled [sic] voracity literaliy [sic] *demolished* the puny favorite’ [62] (p. 2 [emphasis in original]). In another episode in 1808 a terrier was carried off by a dingo that sprang from hiding while the dog’s owner was ‘within a few paces of him’ [63] (p. 1). The manner in which these events were described in the reports seems to imply that such incidents were commonplace in the Sydney colony at this time. In addition, there was a long-standing problem in the early 19th century with dingoes raiding settlers’ dwellings and killing their poultry and domestic dogs along the old Liverpool Road (now Woodville Road) on Sydney’s outskirts [64] (p. 2). This connecting road between Parramatta and Granville was at one stage named the Dog-Trap Road, because the ‘whole countryside was badly infested with [dingoes]’ [65] (p. 9). One source has claimed that at this time dingoes ‘simply swarmed round Sydney … In winter, all animals then had to be stabled at night, or else they would be eaten alive’ [66] (p. 10).

It seems very unlikely that dingoes in the early Sydney colony were ever habituated in the sense that dingoes in parts of modern K’gari are (or were) habituated; that is, with people actively encouraging dingoes to visit townships, dingoes wandering freely in and out of campsites in search of unsecured food and edible waste, tourists enticing the animals with titbits so they can get ‘selfies’ with them, and other forms of interspecies ‘fraternisation’ [43]. The colonists did sometimes take newborn pups from dingo dens on the edges of the Sydney settlement [67], and it is well known that wild-born juveniles were kept as pets, including by the first governor of the colony of New South Wales [68] (pp. 174–175). Generally, however, it seems safe to assume that colonists did not admire wild-living dingoes to the extent that they made trips to observe them in their natural habitat, as modern tourists do in K’gari—they were far more likely to have shot these maligned pest animals on sight [48,67]. Nevertheless, through habitual livestock predation in settled areas, hunting and eating domestic dogs, and stealthily feeding on discarded refuse or scavenging from rubbish tips, dingoes may have come to associate the presence of European colonists with food. Along with habituation, food-conditioning is regarded by wildlife specialists as a motivating factor behind predatory attacks by other canine predators, such as wolves [69,70] and coyotes (*C. latrans*) [26] in North America.

**Figure 6:**
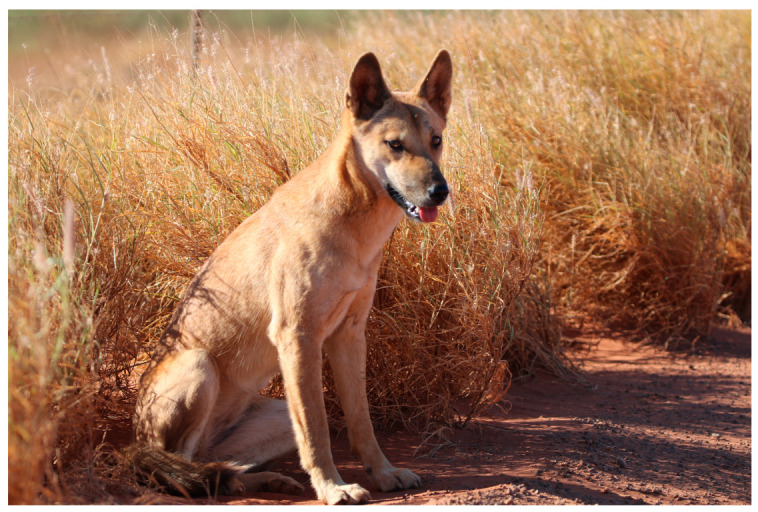
A wild dingo living on a remote mine site in the Tanami Desert. Dingoes are attracted to the rich food waste at rubbish tips and other scavenging opportunities generated by mining operations. Some of the dingoes can easily tolerate being in proximity to humans, in this case allowing the photographer to come close enough to it to take this shot. Credit: Thomas Newsome.

In sum, it is probable that the availability of anthropogenic foods from earliest settlement might have immediately begun to alter the behaviour of wild-living dingoes in the vicinity of the Sydney colony and other white settler communities. The appearance of these supplementary food resources would have brought dingoes into close contact with settlers, thereby raising the possibility of human–dingo conflict occurring, including physical attacks by dingoes on colonists for a variety of reasons (ranging from defensive to predatory attacks). Importantly, this phenomenon is evident today when commercial mining operations are established in remote desert locations where the free-roaming dingo populations previously had little if any contact with humans [71,72]. The mines produce abundant food waste that attracts numerous scavenging dingoes (Figure 6); indeed, some long-established operations harbour genetically distinct populations of commensal dingoes [71,72] that den and whelp within the mine precinct [73]. Negative human–dingo interactions have been documented. For instance, in 2018 a female contractor at a Pilbara mine was badly injured when she was attacked by three young dingoes that approached her while she was eating lunch in an outdoor barbeque area [74]. Much like these mines, colonial settlements generated novel food resources that could have given rise to localised populations of commensal dingoes. Notably, however, the mines are operated by an adult workforce on a ‘fly-in fly-out’ roster; there are no families with children present on a continual basis at these remote industrial facilities. Hence, the risk of dingo attacks in the early colonial settlements, especially predation of the most vulnerable members of the settler community, could have been more acute (see below).

In sum, there are historical examples of dingoes displaying the sort of bold behaviour around humans that is nowadays attributed to the ‘problem’ dingoes of modern K’gari, and which is assumed to be of recent origin. This suggests that the processes that result in habituation and indirect feeding of dingoes could have started early in the history of colonial settlement in Australia. If so, the behaviour of these animals was affected long before the advent of modern tourism in wilderness areas. It follows that a number of the historical accounts of dingo attacks could well be valid.

## 4. Results: Earlier Cultural Attitudes towards Dingoes

The second part of the study is concerned with understanding what past Australians believed about the propensity of wild dingoes to attack humans. Throughout Australian history dingoes have been roundly condemned by pastoralists as relentless killers of domestic livestock, especially sheep [48]. From the earliest beginnings of the sheep production industry dingoes were the ‘scourge of flockowners’ [75] (p. 62), and indeed within a few years of settlement colonists were calling for the ‘total extermination of the ruinous brood’ [76] (p. 2). Early colonial representations of dingoes drew upon European cultural images of wolves, including a rapacious and sinister character and perceived predilection for deception [77,78]. Much as was the case with wolves, dingoes were seen as sly, despicable, cunning, and bloodthirsty curs with ‘excessive and frenzied’ stock-killing habits [77] (pp. 88–89), although the more dog-like physical appearance of dingoes was a noteworthy characteristic of the species (i.e., the canids were commonly referred to by settlers as ‘native dogs’) [78]. Dingoes were also widely regarded as being cruel and pitiless ‘brutes’. This characterisation was probably based partly on observations of dingoes eating sick or bogged cattle while they were still alive (e.g., [79]), as well as the belief that these wild canids killed and mutilated sheep ‘for fun’ [80]. However, above all, the supposedly cowardly nature of the dingo was emphasised: indeed, in Australian colloquialism and ‘slanguage’ the word dingo has long been synonymous with human cowardice (e.g., cowardly people were derisively called dingoes) [77]. As discussed in Appendix E.1, however, it is possible to detect a historical shift in attitudes with regards to the implications of this particular conception of dingo behaviour.

Up until the early decades of the 20th century, Australians commonly regarded dingoes as representing a direct threat not only to sheep and calves and other domestic livestock, but to their own lives as well (Appendix E.1). It is evident from a range of contemporary published sources that there was a longstanding and widely held belief that the cowardly nature of dingoes led them to opportunistically attack, kill, and eat vulnerable humans they encountered in their territory (Appendix E.1). Children who were lost in the Bush were thought to be particularly at risk of falling prey to predatory dingoes, but so too were drunks and vagrants and other lone adult rural dwellers if the dingoes roamed in packs. In fact, in much of eastern Australia the conception that dingoes, on occasion, preferentially targeted these kinds of people in order to kill them and consume them as food was widespread, if not common knowledge, throughout the 19th century and during Edwardian times. Dingo attacks were seen as being uncommon—as one source noted, ‘It is rarely that they attack a man’ [81] (p. 10). Nevertheless, it was widely believed that attacks did happen, and that when in the Bush one had to be on guard against them (e.g., it was common bushcraft to camp with a large fire to keep dingoes away (Appendix E.1). Here, this popular conception is referred to the ‘deadly dingo’ trope.

By contrast, a major shift in thinking about the cowardice of the dingo was at the heart of an opposing view of human–dingo relations that took root in Australian society around a century ago (Appendix E.2). Beginning in the 1920s, and extending into the following decade, a host of dingo-related articles, opinion pieces, and letters appeared in the Australian press that departed markedly in tone from the image of these canids held by prior generations (Appendix E.2). In line with the views of the past century, both rural and urban people of this time despised dingoes as cowardly stock-killers, the so-called ‘red curse’ [82] (p. 9). However, by the interwar period voices that appear to reflect the keenest observers of Bush life were firmly espousing the view that dingoes were incapable of killing or even harming people. These timid canines were so afraid of humans, it was opined, they would flee from the smallest child (Appendix E.2). In fact, by the 1920s and 1930s the various older accounts and oral stories of dingoes attacking lone rural folk, chasing people up trees, and so on, were now viewed in a much more sceptical light, and indeed in some quarters they had become the subject of public ridicule (Appendix E.2). It thus appears that in the decades between the wars it was much less common for Australians to regard dingoes as a direct threat to human safety. Rather, a belief had taken hold that dingoes lacked the temerity to attack a human, and moreover they had *never* been known to do so (Appendix E.2).

As noted, the study’s coverage of the print media from around the late 1950s up until the disappearance of Azaria Chamberlain was not as extensive as it was prior to the late 1950s (see also [32]). The end of this period also coincided with the introduction of television to Australia (1956), an event that profoundly changed how Australians consumed the news [15]. Nothing was found in the wider literature search, however, that would suggest that Australians of the 1950s, 1960s, and 1970s thought of dingoes in a manner that departed dramatically from the beliefs that emerged in the interwar period (see, e.g., the detailed descriptions of dingoes in [83,84,85,86], none of which referred or alluded to wild dingoes making unprovoked predatory attacks against humans; but cf. [87] (p. 94)). A plethora of papers on human–dingo interactions and dingo behaviour generally also appeared in the anthropological literature in the 1970s (for summaries, see [5,21]). There was no explicit mention in any these studies of dingoes attacking humans.

It would therefore appear to be the case that the widespread belief many Australians held in the early 1980s that dingoes do not attack humans can be traced to the 1920s and 1930s—it was thus a relatively new conception in the history of Australian society and culture. It is inferred that the rise and acceptance of this popular narrative in the interwar period created a cultural legacy that: (1) was still in force at the time of the disappearance of Azaria Chamberlain; and (2) influenced public opinion about the plausibility of the so-called ‘dingo baby’ theory in the 1980s.

## 5. Discussion

It is evident that the replacement of the ‘deadly dingo’ trope coincided with the last written records of dingo attacks in the late-1920s (Table A1), but it is not yet apparent why this would be so. Here, in an effort to reconcile these separate parts of the study, two questions are posed: (i) how did the formerly widespread ‘deadly dingo’ trope develop? (ii) Why was it replaced during the early 20th century by a dramatically different cultural attitude towards dingoes? Resolving these problems may help to explain the lack of reports of dingo attacks between 1928 and 1979.

### 5.1. What Caused This Transformation?

An obvious possibility is that prior to the 1920s Australians simply had a flawed view of dingoes as would-be devourers of humans. Notably, there is cause to suggest that belief in the magic and mystical beings of European folklore had a lingering presence in early settler culture, such as witchcraft [88] and Irish Banshee mythology [89]. It is thus possible that irrational thinking distorted colonists’ perceptions of Australia’s fauna. For example, sightings of ‘Bunyips’ (Aboriginal water spirits) were not exceptional (e.g., [90]; see also [91]). The seemingly questionable notion that wedge-tailed eagles (*Aquila audax*) carried off and ate children was also common: for instance, in 1907 the *A. audax* predation theory was used to rationalise the inexplicable disappearance of a lost child’s tracks at Coolgardie ([92]; see also [93] and Appendix C). Stories of eagles snatching babies occur in European folklore [94,95] and Aboriginal mythology [96] (p. 93), with the former suite of myths, at least, having no known basis in empirical fact [94,95]. 

As noted, the first settlers’ hostility towards the dingo partly drew upon pre-existing wolf lore [78]. It may be the case, therefore, that colonists simply projected the ‘big bad wolf’ mythology onto the dingo; combined with the Victorian-era obsession with morality tales and the evil of unrestrained nature, and the censorious nature of the colonial press [29] (e.g., the fate of drunks or errant children was to be devoured by dingoes), this resulted in the dingo being unfairly labelled a murderous villain. Subsequently, by the interwar period Australians had acquired a more enlightened understanding of the dingo and were able to dispel the antiquated beliefs of earlier times.

This scenario is unconvincing. There is no doubt that prior to the early 20th century Australians had very little scientific knowledge of the behaviour of wild-living dingoes. This situation had hardly improved, however, by the 1920s and 1930s—if anything, it had probably become worse, as the rise of the tenacious Alsatian-crossed ‘super dingo’ myth attests (Appendix E.2). The interwar years were a time of scientific innovation [97], but the handful of scholars who studied the dingo were primarily concerned with resolving the issue of the evolutionary history of this canid and its journey to Australia, e.g., [98]—they had little interest in dingo behaviour. It was not until the 1950s that a pioneering scientist (N. Macintosh) began to conduct the first sporadic investigations of dingo biology [83,99], while the first systematic field research into dingo behavioural ecology and social organisation took place in the 1970s [100,101,102].

It is conceivable that early settlers’ conceptions of dingoes as a menace to human life drew upon elements of European folk images of wolves. (A key question is whether the colonial mindset incorporated Aboriginal beliefs about ‘deadly dingoes’, but it is beyond the scope of the study to address this). However, while the dingo has some wolf-like traits it is most definitely not a wolf: it is a distinct canid species that possibly represents an offshoot of early domestic dogs [1,3,4,5]. It is more likely colonists would have come to form their own views of this taxon based on their personal experiences of dingo behaviour, rather than simply transplanting old wolf tales into Antipodean Bush imagery. It seems noteworthy, for example, that by the time of British settlement in 1788 wolves had been extinct in England for nearly three centuries [103]—although only 18 years in the case of Ireland [103] (p. 8). Moreover, most folkloric traditions associated with European wolf mythology (e.g., wolf-reared children, lone wolves lasciviously pursuing young women, werewolves laying siege to villages [103]) were apparently never applied to the dingo. Even the English colonists who settled much earlier in time at Plymouth (1621) did not bring these tropes of Europe’s wolf lore with them to the New World, although they strongly despised its wolves and did all they could to annihilate them [103] (p. 38). Convicts and settlers from other backgrounds (e.g., Germans) might have introduced their own wolf lore-related myths to Australia. It should not be automatically assumed, however, that the power of these beliefs overrode these people’s first-hand comprehension of dingo behaviour and their interactions with this wild canid.

It is clear from the modern incidents at Uluru and on K’gari that wild dingoes *do* fatally attack humans, albeit very rarely, so the 19th century characterisation of this aspect of dingo behaviour was correct in essence (although the circularity of this argument is acknowledged, given that Eurasian wolves also supposedly preyed on people, especially child shepherds [104]). But is that simply a coincidence, or was the development of the ‘deadly dingo’ trope grounded in a factual understanding of dingo behaviour? It also follows that the opposing perception that prevailed in the early 20th century, that dingoes never attack people, was wrong. As will now be argued, however, both interpretations could have been valid judgements at the time: that is, the ‘deadly dingo’ trope may have been based on empirical events, while the narrative that replaced it could *also* have had a foundation in contemporary reality.

### 5.2. Development of the ‘Deadly Dingo’ Trope

It is challenging to separate the empirical events from the cultural milieu in which the historical accounts of dingo attacks were reported. For example, it is not hard to detect the intense hatred of dingoes in the emotive accounts from colonial times of lost children being pursued and eaten by these wild animals (Appendix E.1). This level of animosity would seem to reflect the experiences of a non-Indigenous pastoral community that was traumatised by the depredations of the dingo on flock and herd [48]. However, just because livestock owners detested the dingo it does not necessarily follow that historical accounts of dingoes attacking humans are so biased as to be completely unreliable. It is also possible that newspaper articles on dingo attacks had an influence on shaping the views of the public about the danger represented by these canids (see, e.g., [27]). On the other hand, newspaper reportage of dingo attacks was never very prolific: the published articles are so few and spread out over such a long period that it seems unlikely the colonial press was driving a popular belief that dingoes were deadly—if they were, media reports of dingo attacks surely would have been more common. Finally, there was possibly an element of exaggeration and myth-making that means it is difficult to get to the truth in the historical accounts of dingo attacks, with the danger represented by these canids probably being embroidered to some extent in the Bush yarns and other storytelling traditions of colonial culture (Appendix E.1).

It seems possible, however, that the belief that prevailed in the 19th century that dingoes sometimes preyed on humans was ultimately based on the empirical understanding—the first-hand experience and observation—of rural settlers. Of note is the casual manner in which Australia’s first newspaper, the *Sydney Gazette*, in one of its earliest print editions, attributed a toddler’s death at Prospect in 1804 to dingo predation (Appendix D, Table A1). This implies that it was already common knowledge by this time that dingoes had the propensity to subject humans, or at least small children, to predatory attacks. On this basis, it seems possible to speculate that one or more unreported dingo attacks did, in fact, take place in the fledgling Sydney colony in the pre-newspaper era (1788–1803). Perhaps a child was opportunistically taken by a dingo when these canids first began to infiltrate the settlement to hunt livestock (and dogs). This possibility cannot be ruled out, given the accounts that suggest some dingoes were bold enough to enter houses, and in light of the fact that in many cases we simply do not know how children died in the early penal settlement at Sydney; indeed, according to one source: ‘No evidence from a medically qualified source exists as to the cause of death of any one of the 77 children who died between 1788 and 1792’ [105] (p. 13). Dingo attacks could possibly also have occurred in the early period of settlement in more outlying districts where ex-convicts and free settlers lived on rough Bush farms and isolated grazing runs established in dingo territory. 

In keeping with the current understanding of human–dingo interactions [17,18] any such attacks are likely to have been very uncommon events. However, serious incidents could have been preserved in oral traditions that were transmitted intergenerationally within close-knit rural communities. (In many isolated districts the experiences and recollections and stories of whole generations of colonists were preserved largely in the oral history of the community, and typically were never written down, e.g., [106]). The Bush yarns that survived over time may have formed the basis of the ‘deadly dingo’ trope that first becomes evident in the textual record in the latter half of the 19th century.

If a reasonable argument can be made that at least some people were subjected to unprovoked dingo attacks in early Australian history, then there is a discrepancy in the number of reported incidents that requires discussion. The data imply that dingo attacks caused 28 fatalities over a 121-year period beginning in 1804, including the deaths of 19 children, most recently in 1925 (Table A1). Even one-third of this total would seem to be incongruously high, given there have been only two deaths from dingo attacks in modern Australia (1980 and 2001), both of which occurred in tourist areas where it is widely contended that the high volume of recreational campers had altered the behaviour of local dingo populations [17,18]. There was obviously no equivalent in colonial times of a place like K’gari, where modern transport systems and a lucrative tourism industry bring throngs of visitors into close contact with dingoes in their natural habitat [17,18].

On the other hand, it is conceivable that there were dingo predation risk factors associated with historical cultural practices and land settlement patterns. For instance, beginning in the 1860s the free-selection movement led to the proliferation of small-scale agricultural production in Australia [107] (p. 88). The colonies’ Selection (or Land) Acts broke up large landholdings owned by the pastoral elite (‘squatters’) into smaller lots for lease to farmers, known as selectors [107]. The broad objective of this government-sponsored initiative was to populate the countryside, replacing the small, predominately all-male rural labour forces of pastoralism with a ‘farming yeomanry’ that was based around the small-acreage family farm [107]. Those taking up selections often consisted of families with several small children (completed families of 10–12 children were the norm at this time [108] (p. 304)). The demands of heavy work and care of babies often meant that young ones (<9 years of age) were poorly supervised (for recollections of life on a 19th century selection, see [109,110]. (Owing to the age structure of frontier society there were few grandmothers and older women to help with childrearing [108] (p. 308)). Children were also expected to contribute to farm labour, taking them away from the home on their own at a tender age [109,110]. The holdings available to selectors were often ‘unimproved’ blocks comprising dense scrub that they were contractually obliged to clear and farm [107]. Dingoes would have been common in such habitats, along with animals such as wallabies and rabbits which were their main prey [1].

Such circumstances could have elevated the likelihood of dingo predation against Bush children in particular. (It is certainly thought that the number of lost-child episodes increased considerably during the era of the Selection Acts [111]). As indicated, several historical accounts of predatory dingo attacks involved selectors’ children (e.g., the Willie Gesch case (Appendix C)). The idea of the young child of a selector straying into the Bush and falling prey to dingoes was also a familiar element of storytelling traditions at this time (Appendix E.1). The risk of dingo predation could have been highest during the initial phases of selection in a given area, before selectors had cleared the countryside of the vegetation that afforded game, and hiding places, for dingoes. On the other hand, once the local dingoes’ natural food resources had been diminished through habitat destruction and other land management practices (e.g., pest control) the risk of predatory attacks against children might have increased, especially during the whelping season when dingoes were under particular food stress. As noted, areas under selection often formerly consisted of vast pastoral stations or estates operated by a handful of men [107]. Moreover, after the end of the free-selector/closer settlement era (and the soldier settlement schemes that followed both wars [107]) districts that had been cleared and farmed by selectors mostly returned to their former status as large-scale sheep runs or cattle stations inhabited by very few people or became the site of modern urban sprawl [107]. Hence, there have been few other times in Australia’s European history when large numbers of people who were vulnerable to dingo predation were resident in the Bush.

In addition to selectors, there were shepherding families with small children living in huts on remote sheep runs where dingoes preyed on their stock, as well as young child shepherds who managed flocks on their own [112] (p. 70). In the second half of the 19th century there were also countless ‘swaggies’ (itinerant Bush labourers) and other homeless rovers tramping on foot throughout dingo territory [107,113]. Indeed, one historical account of a dingo attack involved the death of a child shepherd, and several others attributed the loss of shepherds’ children to dingoes (e.g., after they wandered away from huts), while there are also accounts of swaggies being attacked (Table A1). 

In short, the lack of a colonial equivalent of a tourism industry based on recreational camping in dingo habitat might not have prevented the close contact between people and dingoes that is a recurring factor in the modern attacks [17,18]. In fact, the risks associated with dingo predation in rural areas of mainland Australia might have been higher prior to the early 20th century than at any time since.

On this point, the ratio of non-fatal dingo attacks (*n* = 24) to fatal attacks (*n* = 28) in the historical accounts also requires discussion. It is implausibly low. Indeed, on modern-day K’gari there has been just one fatality (Clinton Gage in 2001), and hundreds of non-fatal dingo attacks or negative interactions. Some of the latter have been quite serious (see Appendix B); however, most comprised minor incidents that resulted in no harm to people or only very slight injury, such as biting, nipping, lunging, threat displays, and so on [17,18]. It follows that if the figure of 28 fatalities in the historical period can be taken at face value, then many more ‘lesser’ incidents are likely to have gone unreported over the 121 years until 1925. Based on modern occurrences on K’gari [17,18], such incidents could have varied from people in historical times being bitten by a dingo to being growled at by one, harried by a pack, chased up a tree, and so forth. However, even near-catastrophic attacks might never have entered the written record, such as a dingo seizing and dragging off a small child before being chased away, leaving the youngster with minor wounds. As noted, this is precisely what happened at Uluru National Park in June 1980 [46] (p. 281), but the story might never have been brought to light were it not for the disappearance of Azaria Chamberlain not long afterwards and the media maelstrom that ensued [14,15].

### 5.3. Rise of the Popular Belief That Dingoes Do Not Prey on People

Why was there a shift in the cultural image of the dingo during the interwar period? Social factors are likely to have had a role. The 1920s in particular was a time of change and modernisation in Australia, including a decline in rural life in concert with increased urbanisation and a growing rural-urban divide [97]. The idea of the Bush in the myths of a modernising society invariably transformed as the drift to the cities and suburbia gathered pace [114]. Influenced by a desire for ‘peacefulness’, for example, Australians of the interwar period increasingly neglected the colonial-era image of the Bush as a fierce wilderness that needed to be tamed by the pioneers, and began to view it through the lens of a rural idyllic past [115]. Country town life in Australia became likened in popular culture to English small village imagery [115], while literary representations of the Bush from this time were inclined to portray its native fauna as charming and defenceless and lacking dangerous predatory creatures (e.g., [109]). Perhaps, in this nostalgic reimagining of the Bush that flourished between the wars [115], the old image of the ‘deadly dingo’ was more evocative of a threatening jungle than a tranquil countryside, and was duly sanitised (see also [116]). However, even if so, it is likely that the shift in attitudes was ultimately grounded in proximate causes that had more to do with changes in human–dingo relations than purely ideological considerations.

A key reason that Australians began to think differently about wild-living dingoes in the early 20th century may have been simply because the frequency with which they interacted closely with these canids had declined considerably by this time. This was especially the case in the more densely settled districts of south-eastern Australia, areas located within the ‘Dingo Fence’ [117,118] (Figure 2), and where most of the human population was concentrated. The introduction of strychnine in the 1840s led to the near extermination of dingoes in the well-settled districts of Victoria by the early 1850s [112] (p. 146). By the 1930s the dingo population had also largely been eliminated from most of New South Wales [119]. Analysis of historical records shows that between 1883 and 1930 over 280,000 dingo scalp bounties were paid in New South Wales [119]. At the beginning of this period, dingoes seem to have been distributed in high densities throughout most parts of the state. Based on these data, it has been contended that the distribution and abundance of dingoes in New South Wales peaked at the end of the 19th century [119]. By 1920, however, ‘the distribution of the dingo appears to have been greatly reduced’ [119] (p. 435). At this point in time some districts had ceased to offer bounties for dingo scalps owing to a lack of demand, while others reported no scalp returns. It is inferred that by 1930 dingoes had been eliminated or were scarce in all but the north-eastern corner of New South Wales [119]. The most rapid decline occurred in the southern and central regions, where the highest numbers of sheep were grazed [119]. The combination of fencing and heavy strychnine baiting nearly wiped-out dingoes in this region. Beginning in the 1870s, stock-owners were rapidly building fences in eastern Australia, and by the mid-1880s >95% of sheep in New South Wales were in paddocks [112] (p. 143). Open camping of sheep in fenced paddocks replaced shepherding and was only possible when dingo numbers were brought under control [112]. The modern sheep industry in mainland Australia is largely confined to areas that are located within dingo exclusion fences [120].

In sum, the rarity of dingoes in much of south-eastern Australia by the 1920s and 1930s suggests that people in this densely settled region encountered dingoes much less frequently in the wild at this time, reducing the likelihood of claims arising of people being attacked by them. This would help to explain why the dingo was being represented in new ways in the Bush stories and images that remained powerful forces in Australian nationalism and culture during the interwar period—but were undergoing change [107]. It may be the case that as the dingo gradually disappeared from the interwar rural landscape the cultural image of this species became more susceptible to romanticisation, until the old-style ‘deadly dingo’ stories were winnowed from the corpus of foundational Bush myths. Other iconic figures of colonial-era rural Australia underwent similar transformations during this time. For example, the popular image of the rough but honest 19th century frontier dweller became ever more valorised and simplistic [121]. Similarly, shepherds and swaggies and other itinerant Bush workers of colonial times had long been romantic characters, but as living memories of these rural types faded, they were the subjects of a vigorous strain of sentimentalisation [121,122].

On the other hand, it is evident that even in areas where wild dingoes were intensively persecuted rural dwellers still had at least some contact with the living animals—they did not disappear completely [120,123]. Moreover, it is unlikely that the cultural shift in attitudes towards dingoes can be attributed to a severance in the lineal transmission of knowledge of past dingo attacks in the form of oral stories, family histories, and published newspaper articles. As noted, most of the historical accounts of dingo attacks are from the latter half of the 19th century (Table A1). A person who was in their 20s in the 1890s was in their 50s in the 1920s—a few even had living memory of convict times in the 1830s [97] (p. 17). It therefore seems unlikely there was a simple loss of collective memory of the ‘deadly dingo’ trope.

The attitudes towards dingoes and the issue of human safety in the early 20th century differ so conspicuously from those of the 19th century (Appendix E.1) it is as though the observers were describing entirely different animals. It therefore seems possible to argue that decades of intensive human persecution, especially in closely settled areas, had not only greatly reduced the dingo population but also conferred an adaptive advantage on those individuals that were much more wary of humans [17,124]. Notably, anecdotal reports suggest that dingoes that persisted in pastoral districts where they had been extensively baited, shot at, and pursued with traps for decades were highly elusive [125]. Some ‘outlaw’ dingoes killed countless sheep over a period of years, evading the attentions of even the most skilled trappers and hunters [86,126]. The survival of these animals seemed to depend on their overriding propensity to avoid coming into contact with humans, whether directly or indirectly. As one authority remarked: ‘Let the dingo once get the smell of a human being on anything, and he will then slink away into the forest, satisfied to leave well alone’ [126] (p. 7). Indeed, as one authority contended: ‘Before dingoes became aware that they would be poisoned, shot, trapped or run down, they did not have the same tendency to avoid people as they do now’ [48] (p. 86). Another noted ‘dingologist’ has endorsed this view:

In certain locations on the mainland, almost two centuries of artificial selection pressure from lethal control practices has undoubtedly led to a skew in some resident populations in relation to responses to human stimuli, away from boldness and towards something suggestive of a fear response

[17] (p. 141)

Other historical changes in dingo behaviour in response to human persecution might also have been relevant. For example, the ‘deadly dingo’ trope features the recurring narrative that dingoes, being cowardly animals, would normally only attempt to attack rural dwellers if they outnumbered them significantly (Appendix E.1). This belief is supported by the historical accounts in Table A1 (see also Appendix D). The accounts most frequently described people being attacked by multiple dingoes, and much less commonly by a solitary dingo—in contrast, 78% of recent attacks on K’gari involved a lone dingo [18]. In two cases, the historical accounts reported 20 or more dingoes being involved in attacks (Table A1). Present dingo group size is typically around 3–12 individuals [51] (p. 27); modern dingoes are occasionally observed congregating in larger numbers (e.g., up to 17), but mostly only in association with rich human-created food patches, such as mine site waste facilities [127]. It therefore could be the case that at certain times in the 19th century dingo group sizes in some areas were larger than today, and that this heightened the risk of attacks on humans. Indeed, according to one pastoralist: ‘There were some who asserted that men had been attacked by dingoes in the days when the dogs used to hunt in big mobs of 50 or 80’ [128] (p. 16). While this seems like an implausibly high number, it is worth emphasising that the dingo population is thought to have exploded across south-eastern Australia during initial settlement, as novel sources of prey (livestock and feral game) and water (dams and artesian bores) first became available [1] (p. 136). Hence, dingoes of the interwar period may not be directly comparable with their historical counterparts in terms of group size and related social dynamics and behaviour.

To summarise, it seems warranted to infer that the reason for the shift in thinking during the interwar period about the propensity of dingoes to attack humans is owing to: (i) a numerical cause (i.e., people and dingoes interacted less frequently); and (ii) a behavioural cause, as owing to the dingo mass kill-off the average rural dweller’s experience of dingo behaviour by the 1920s and 1930s probably did differ from that of prior generations—the error was in assuming that wild dingoes had always behaved in that way. These two scenarios are not mutually exclusive, and both causes were probably operating in some areas. Major transformations that have occurred in Bush life and culture since early in the 20th century (e.g., end of the free selector era) may have also lessened the risk of dingo predation.

The situation is more complex in Queensland and other regions where dingo populations have remained high despite a long history of persecution. For example, a 2014 survey of rural Queensland landholders reported that 93% of respondents had a dingo ‘problem’ on their property [129]. If dingoes are so plentiful in mainland Queensland, why have there apparently been no reports of attacks in the decades between the early 20th century and the recent well-known cases from K’gari? The simplest answer is that many years of systematic lethal dingo control campaigns have altered the phenotypic behaviours of dingoes, favouring the survival of those that were much less likely to interact with humans than their historical counterparts. Historical changes in rural life might also have reduced the exposure of vulnerable persons to dingo predation, as previously noted. 

Furthermore, there may be a long history of underreporting of dingo attacks. For instance, if dingo attacks did occur in rural districts between 1928 and 1979 victims may not have reported them, as it was no longer *comme il faut* to make such claims within the social milieu of Bush culture. Rare modern cases of dingoes attacking humans in peri-urban areas of mainland Queensland may also often have been erroneously attributed by the public (and authorities) to feral domestic dogs or hybrids with negligible dingo ancestry (Appendix F). As previously indicated (Appendix E.2), the popular cultural belief that ‘pure’ dingoes are afraid of humans and that only dingo-dog hybrids are potentially dangerous is part of the same dingoes-are-harmless complex that appears to have arisen between the wars. It seems that this component of the complex has persisted into the present day, whereas the allied notion that dingoes do not attack humans under any circumstances has largely been phased-out in recent decades in the wake of the Chamberlain-Gage fatalities.

It seems worthwhile noting that a strikingly similar pattern is evident in the current perception of human-wolf interactions in North America. The present consensus is that there were no unprovoked attacks by healthy, non-rabid wolves in the United States and Canada between 1900 and 1969 [69], while attacks in the last half-century are extremely uncommon [70]. By contrast, a number of accounts of wolves killing and consuming frontier dwellers, stalking lost travellers, ‘treeing’ people and so on, were documented in the 19th century (and earlier than this [130]). Much as was the case with the ‘deadly dingo’ trope in Australia, at that time it was commonly believed that the North American wolf most certainly represented a physical danger to humans [41,103].

However, authorities in wolf behaviour have long questioned the reliability of these accounts [130]. Early stories of wolf attacks are held to be implausible and heavily influenced by Old World folklore, if not outright perfidies [104,131,132,133]. The perception that there is no credible historical evidence for unprovoked predatory wolf attacks, combined with the absence of verified incidents over most of the 20th century—augmented by a tradition of literary environmentalists (e.g., Farley Mowat) idealising the wolf [134]—has led to the rise of the popular modern narrative that North American wolves simply do not prey on people [69,104,133]. Similarly, the handful of wild wolf attacks that have occurred in modern times have been interpreted as anomalies because these wolves had supposedly been habituated to the presence of humans, and therefore did not behave like ‘typical’ free-roaming wolves [69,70,133].

But if the dingo model proposed here is valid, an alternative interpretation is possible: that the hiatus in wolf attacks in the 20th century was largely owing to the precipitous decline in wolf populations through systematic human persecution (i.e., by the early 1900s wolves were practically extinct in southern Canada and the lower 48 states [70] (p. 832)), reducing the frequency of human-wolf interactions, and selecting for a heightened wariness towards humans in the remnant wolf populations [69,130,131,132,133]. There is now a growing (although still miniscule) body of evidence for unprovoked predatory attacks by healthy wolves that were not habituated or food-conditioned [104]. Most notably, in 2010 a young woman was killed and eaten by wolves near Chignik Lake in Alaska, as clearly indicated by snow track evidence and DNA analysis of bite marks, among other findings [135]. It follows that the dismissal of all historical accounts of wolf attacks in North America as falsehoods or dubious folk legends may be unwarranted.

## 6. Conclusions

This paper examined the origin of a popular belief that was prevalent in Australia at the time Azaria Chamberlain was attacked and killed by a dingo in August 1980: that dingoes do not attack and kill humans. To address this problem, a search was conducted for published accounts of dingo attacks in earlier Australian print media and other historical sources. In total, 52 accounts of attacks spanning the period between 1804 and 1928 were identified, including 28 supposed fatalities (Table A1). The paper then delved deeper into the cultural milieu in which these attacks were reported. It is evident that up until the early decades of the 20th century it was a commonly held belief in Australia that dingoes were formidable predators that were known to actively hunt, kill, and eat humans, albeit rarely (Appendix E.1). This past narrative is referred to here as the ‘deadly dingo’ trope. Further work showed that this once popular conception had largely been replaced by the 1920s and 1930s with the equally prevalent view that the dingo was a benign animal that did not attack people unless it had been provoked (Appendix E.2). 

It is difficult to separate myth from reality when it comes to anecdotal accounts of wild animals attacking humans in the early period of Australia’s history. The colonial-era narrative has the ‘feel’ of a folk belief inherited from European wolf lore; it cannot be definitively called a myth, however, because it has elements that conform to what we know about the pattern of modern dingo attacks. Indeed, when compared with recently documented dingo attacks on K’gari (1990s to present [17,18]) some of the historical accounts do appear to be tenable. Importantly, the historical accounts also show the same strong seasonal trends as modern attacks (i.e., they commonly occurred during dingo breeding and whelping seasons [18]). Owing to the deficiencies of the historical accounts it is not possible to verify whether up to 28 people really did die from dingo predation in the pre-1930s era, but it does seem unwarranted to conclude that no one was *ever* killed by a dingo in these times. It would be worthwhile expanding this discussion to include other predatory Australian taxa (e.g., goannas, crocodiles, snakes, thylacines).

These findings add a new perspective to the Australian public’s complex response to Azaria’s disappearance, and its condemnation of her mother Lindy [6,7,8,9,10,11,12,13,14,15,16]. Australians’ collective disbelief about the dingo theory was based both on contemporary perceptions of dingo behaviour and on the accepted understanding of human–dingo interactions over the longer course of Australian history (Appendix A). However, the ‘deadly dingo’ trope seems to have all but faded from living memory by the outbreak of World War II (Appendix E.1). During the period of the Chamberlain trial some elderly people may have recalled media coverage related to the most recent dingo attacks in Table A1 (e.g., turn of the century to late-1920s). There appears to have been no such reports, however, in the half-century prior to the disappearance of Azaria. It is therefore little wonder that in the early 1980s so few members of the Australian community had ever heard of a person being attacked by a wild dingo [6,9]—this would have included Baby Boomers and anyone who was then aged less than about 50–60 years old (that is, 86.1% of the 1981 population [136]). By that time stories of dingo attacks had simply ceased to be a part of mainstream Australian culture.

It can be concluded from this that the interwar cultural shift in the image of the dingo marked the onset of the Chamberlain-era attitude towards these canids. As noted, most Australians in the 1980s earnestly believed that Lindy’s claim that a dingo killed her child was, at best, questionable, and at worst a preposterous lie (Appendix A). In reaching this conclusion some may have felt they were drawing upon empirical knowledge of dingo behaviour that stretched back to the time of the country’s first rural pioneers. On the contrary, the perception that dingoes do not attack humans was a legacy of the recent past of a modern urbanising society, apparently dating to no earlier than the 1920s. This disconnect between the views of Australians who were separated by only a relatively short span of time perhaps reflects the degree to which 20th century culture had drifted away from the real narrative of the nation’s colonial heritage (see, e.g., [107]).

It would be an overreach to suggest that the new attitude towards dingoes that took root in the interwar period was responsible for the false conviction of Lindy Chamberlain, but it surely had a key role. In effect, it imposed a limit on what was then considered to be within the bounds of reasonable possibility when it came to the actions of a given wild dingo (to paraphrase the legalistic jargon used in the 1987 royal commission [46]), and thus it lay at the heart of the incredulity with which Lindy’s ‘dingo theory’ was greeted [6,10,116,137,138]. Indeed, it is fascinating to speculate what might have happened if Azaria had vanished from a rural dwelling in, say, Queensland in 1880, instead of a century later in the Northern Territory. The colonial-era Lindy, having claimed to have witnessed a dingo carrying off her child, most likely never would have been charged with murder. In all probability she would have been treated with sympathy by the police and public alike. Most importantly, she would have been believed.

How did the ‘deadly dingo’ trope of the 19th century emerge, and why did Australians of the interwar period come to harbour such a different understanding of the propensity of dingoes to attack humans? These are complex problems to resolve. Concerning the former, it is suggested that colonial-era agricultural practices and land settlement patterns may have raised the risk of human–dingo conflict in rural areas to a level that we have only seen resurface over recent decades (albeit greatly amplified) on K’gari in particular, where habituated dingoes regularly come into contact with tourists. With regards to the latter, it is proposed that the change in attitudes towards dingoes that became apparent in the 1920s and 1930s may be owing to the effects of decades of shooting, trapping and poisoning almost eradicating the dingo population in eastern Australia by this time. This reduced the frequency of human interactions with this canid and may have selected for increased wariness around humans in dingoes that survived.

The intent of this paper is not to contribute further to the demonisation of dingoes, but to instead help form a more accurate picture of the behaviour of this iconic wildlife species. Managing and conserving a species requires understanding it more fully. Based on the fragmentary evidence available it seems likely that dingoes did not attack people on a regular basis at any period of Australian history. Similarly, modern dingoes do not represent an irreconcilable danger to human life. Even in K’gari, where negative interactions between humans and dingoes occur with some regularity, serious attacks have always been exceptionally rare events [17,18]. Indeed, given the abundant opportunities these canids have to attack people in this popular family holiday spot, it is germane to ask why they do not do so more often [17]. Nevertheless, the historical accounts of dingo attacks examined here do suggest that current conservation and management strategies may be perpetuating an inaccurate view of this apex predator. Presently, it is widely contended that dingoes have the propensity to attack and kill people (especially small children) only in popular tourist locales or other contexts (e.g., remote mines) where dingoes have become habituated to humans and/or food-conditioned, and not in rural or wilderness places in general, or for that matter in peri-urban areas where wild dingoes have a ubiquitous but little-known presence [139,140] (Appendix F). There is also a pronounced tendency to regard the conditions under which dingoes become habituated and/or food-conditioned (and thereby dangerous) as innately modern phenomena that have only existed in the last few decades. Should we accept these views given the historical accounts of attacks described here? Or should we acknowledge that a wild dingo in any area *may* regard a human as prey under certain circumstances and/or if the opportunity arises, and that ‘problem’ dingoes may not be a historically recent phenomenon? If we accept the latter view, then we must move past the entrenched cultural image of the dingo as ‘just a dog’ (Appendix A), and accord this wild canid the healthy respect it is due as a top-order predator. We must also look at the historical accounts of dingo attacks with less scepticism.

## Figures and Tables

**Figure A1:**
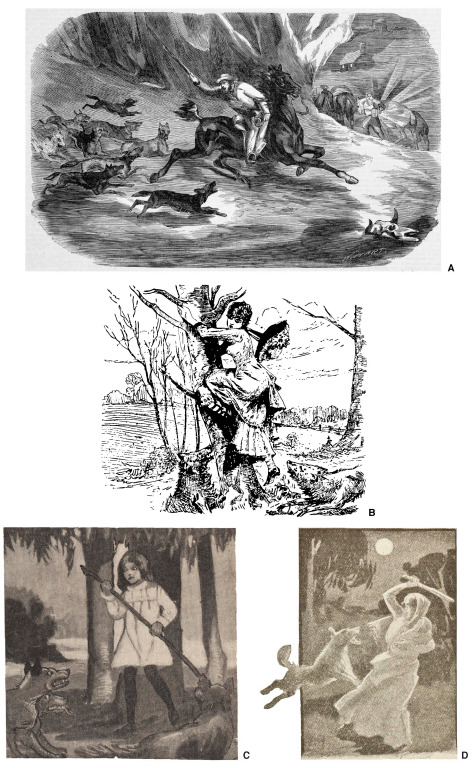
Early representations of dingoes attacking humans. Some images relate to historical accounts of dingo attacks (**A**,**B**), and thus purport to be factual, while others are from fictional stories (**C**,**D**). (**A**) Dingoes attacking a lone rider. This incident reportedly took place in the Para River region in the mid-19th century [81] (Table A1); (**B**) A pair of dingoes ‘treeing’ a woman. Published in the *Bulletin* in 1884 (redrawn from the original using the Adobe Illustrator Live Trace tool (setting: Default)), this image was based on a contemporary account (1884) of an incident that occurred in the Dawson River area: a young female cattle drover was supposedly chased up a tree by a pair of dingoes when she intervened in their attempt to kill some cattle [210] (Table A1). In fitting with the gender stereotyping of the late Victorian era the cattlewoman has been improbably represented as a coquettish ingénue in an elegant dress; (**C**) Dingoes cornering a lost child. This is an illustrated scene from the fictional story of ‘Warrigal’ the wild dingo [211] (p. 48). Here, the young daughter of a selector, who had strayed into the wilderness, is surrounded by hungry wild dingoes. In the climax of this narrative the story’s antihero, Warrigal, scares away the predatory dingoes and keeps the child safe until she is rescued; (**D**) A dingo attacking a settler woman and baby. This image by an unknown artist accompanied a 1920 fictional story set in colonial New South Wales [212]. All images are out of copyright. Sources: (**A**) ‘The Dingo Or Native Dog’ (artist: Walter Hart (27 July 1866)). Reproduced with the permission of the State Library Victoria; (**B**) Title: ‘Tree’d by dingoes: a pretty girl on the Dawson River, Queensland, takes refuge from the attentions of native dogs of too much taste’ [210]. (**B***–***D**) Reproduced with the permission of the National Library of Australia.

**Figure A2:**
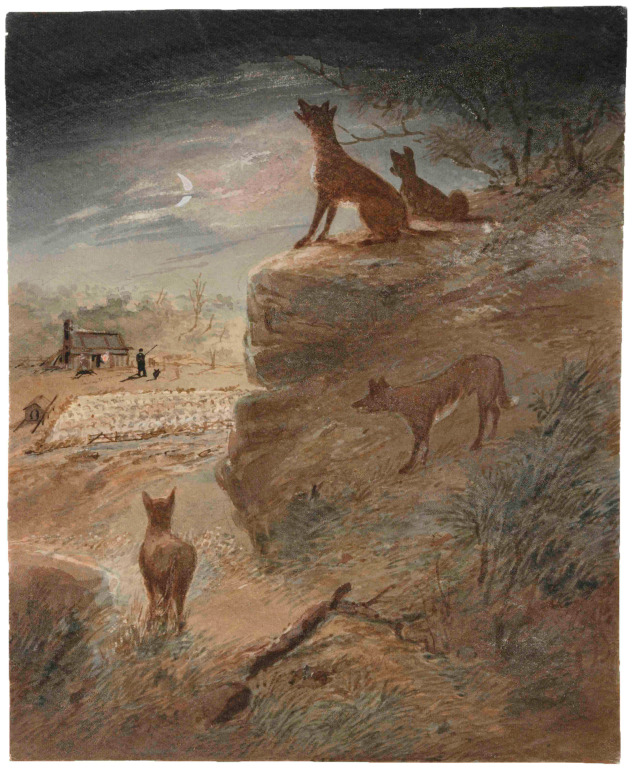
A depiction of dingoes howling near a shepherd’s hut. Title: ‘Prospecting at an out station. Dingoes, or wild dogs of the Bush prowling round the sheep fold; Dr Doyle’s sketch book, ca. 1862–1863/John Thomas Doyle & Samuel Thomas Gill’ (PXA 1983). Reproduced with permission of the State Library of New South Wales.

**Figure A3:**
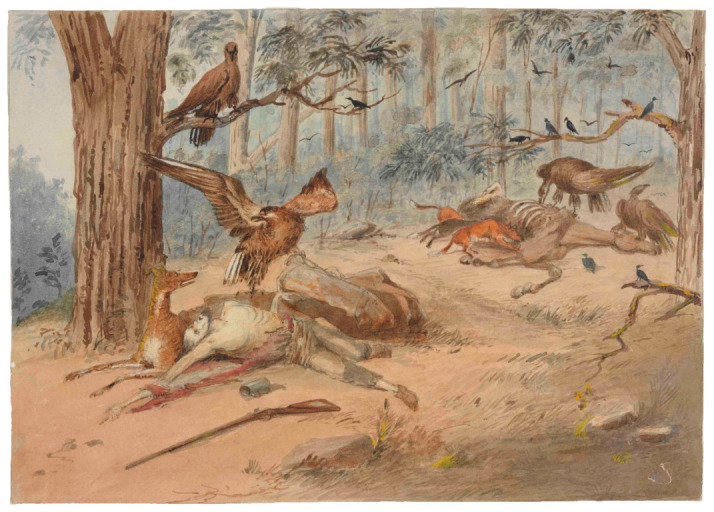
A depiction of dingoes scavenging the body of a man who died while lost in the Bush. Title: ‘“Lost in the Bush” close to the dividing Ranges between N.S. Wales. A sketch taken on the spot by J. F. Doyle’; Dr Doyle’s sketch book, ca. 1862–1863/John Thomas Doyle & Samuel Thomas Gill (PXA 1983). Reproduced with permission of the State Library of New South Wales.

**Figure A4:**
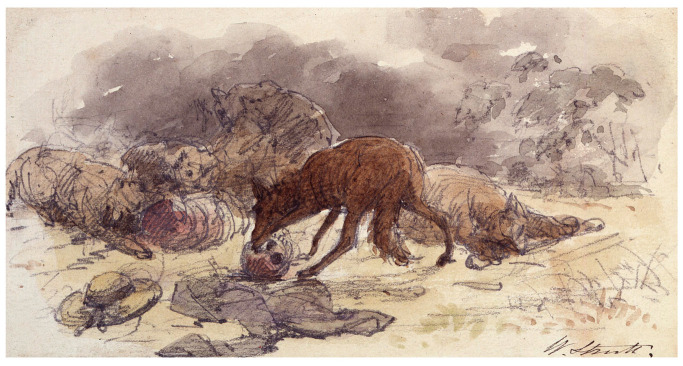
A depiction of dingoes scavenging the remains of explorer William Wills. Title: ‘Dingo’s [*sic*] devouring Wills’ remains’ (1879?). Artist: William Strutt. Image courtesy Parliament of Victoria.

**Figure A5:**
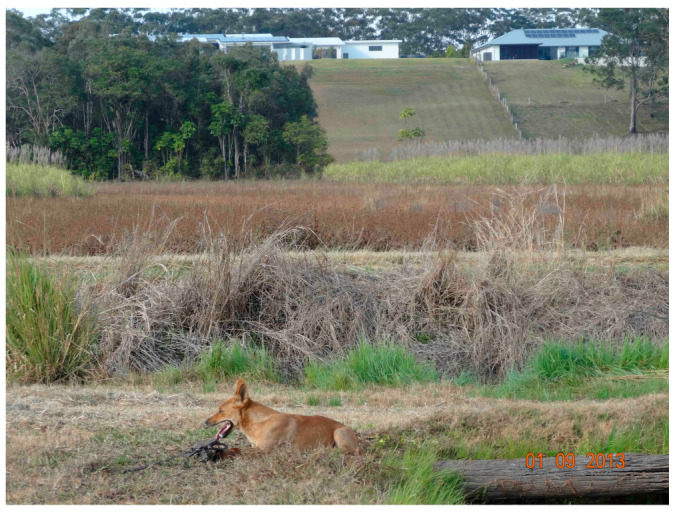
An urban wild dingo. This photograph was taken in September 2013 in a sugarcane field at Bli Bli on the Sunshine Coast, south-eastern Queensland. Note the proximity of residential housing. The dingo is caught in a soft-catch foothold trap, which prevents injury. This individual was fitted with a GPS collar and released unharmed as part of an urban wild dingo tracking study. Credit: Benjamin Allen.

## Data Availability

Not applicable.

## Appendix A. The Chamberlain Case

On the evening of 17 August 1980 an event took place that thrust the nature of the human–dingo relationship into the collective consciousness of all Australians. At around 8 pm that night a nine-week-old baby, Azaria Chamberlain, disappeared from her family’s tent at a holiday campsite at Uluru (‘Ayers Rock’) in central Australia [6,7,8,9,10,11,12,13]. Azaria’s mother, an Australian-New Zealand woman named Alice Lynne ‘Lindy’ Chamberlain (*b*. 1948), had just put the baby to sleep in the tent and was metres away with her husband, small son, and two other campers, when Azaria cried out. As Lindy went to check on the infant, she saw a dingo emerge from the open doorway of the tent with something in its mouth [6,9]. It quickly ran off, and when Lindy inspected the tent seconds later, she found her baby missing. She chased after the animal, crying ‘A dingo’s got my baby’ (or words to that effect), but it vanished into the darkness [6,9]. A search revealed blood in the tent. Moreover, expert trackers from the local Pitjantjatjara community, the traditional custodians of Uluru—and fonts of knowledge on dingo behaviour (although it was mostly ignored by authorities and dismissed by the public prosecutor (the ‘Crown’) [6,9])—observed dingo tracks in the vicinity of the tent and identified where Azaria’s body had been placed on a sandhill while the animal rested. A week later her torn and bloodied jumpsuit was found several kilometres away near a dingo den, but her remains were never recovered [6,9].

In February 1981 a coronial inquest found that a dingo had seized the infant from the tent and carried her off and eaten her. Following further investigation, however, the Northern Territory police charged Lindy with Azaria’s murder. During the 1982 trial the Crown predicated that a homicidal Lindy had taken Azaria from the tent, cut her throat in the family car, and then disposed of her body and planted the clothing at the den to conceal her crime [6,9]. Lindy’s claim to have witnessed a dingo in the tent was dismissed as ‘“preposterous … an affront to your intelligence … [and] a transparent lie”’ [141] (p. 179). The jury accepted the version of events submitted by the Crown, and in October 1982 Lindy was convicted and sentenced to life in prison (her then-husband Michael received a suspended sentence). After three-and-a-half years of incarceration, and having exhausted all avenues of appeal [142], Lindy’s conviction was overturned in 1986 following the chance discovery of Azaria’s matinee jacket [16]. (It was found when rangers were investigating the death of a tourist who fell from ‘The Rock’ and was partly eaten by dingoes). The Crown had rejected the dingo theory partly on the basis that there was no dingo saliva on Azaria’s jumpsuit, a fact which Lindy explained as being due to her having put the matinee jacket over the jumpsuit [16,141]. The discovery of this missing item of clothing proved she had been telling the truth [6,9,16].

The Chamberlain affair was a watershed event in the story of modern Australia [14,15]. Azaria’s disappearance captivated the 1980s public in a way no missing children stories had before [143]—indeed, it is believed that as many Australians watched the live television announcement of the first coroner’s report (two million) as watched the moon landing in 1969 [136] (p. 23). The level of interest in the story was unprecedented for the time, and some scholars now refer to it as the nation’s first true ‘media event’ [15]. Numerous books and academic papers have been published about the case and its ongoing cultural legacy, while the story has been dramatised in several television mini-series [15]. It is best known to international audiences through the 1988 Fred Schepisi film *Evil Angels* (*A Cry in the Dark*), with Meryl Streep playing Lindy. The phrase ‘A dingo’s got my baby’ has become a global pop culture meme [15].

The factors underpinning Lindy’s wrongful conviction have been analysed many times [6,7,8,9,10,11,12,13,14,15,16]. The testimony of the scientific ‘experts’ was a key issue: the Crown’s case relied heavily on flawed forensic evidence (e.g., ‘foetal blood’ in the family car—later revealed to be sound-deadening paint) that made a dingo attack seem inconceivable [6,9,16]. The weight of public opinion was also swayed by media-driven hysteria, with the affair now widely regarded as a case study in trial by media or tabloid justice [14,15]. Lurid claims were aired about the Chamberlain’s ‘cult-like’ religious beliefs (they were Adventists), including the insinuation Azaria—whose name some media outlets falsely reported meant ‘sacrifice in the wilderness’—was murdered in a pseudo-satanic rite [137]. Lindy’s emotional state in TV interviews and court appearances was also held to be inconsistent with that of a grieving mother [6,9]; indeed, the couple’s ‘stoic piety in the face of tragedy ran so counter to Australians’ cynicism and fear of spirituality that it sparked off a virtual witch-hunt’ [144]. At the time of her conviction Lindy was the most hated woman in the country (70% of Australians accepted the judiciary’s view that Lindy was guilty of infanticide)—many refused to accept her innocence even after the verdict and sentence were quashed [15]. There are indications that the Northern Territory police and judicial authority were biased towards Lindy and that this enmity denied her a fair trial [6,9].

The intricacies of the Chamberlain case suggest there was no one reason why so many Australians wrongly believed Lindy had murdered her daughter. Notably, however, there is a longstanding trend among the various social commentators, cultural theorists and other members of the wider intellectual community who have studied the case (‘Chamberlain-ologists’ [15]) to conclude that the jury was simply bamboozled by the barrage of unsound forensic evidence presented by the Crown, and/or prejudiced by the scurrilous media portrayals of Lindy’s character [15,145]. While this is true to some extent, it is clearly not the full story—it overlooks the fact that the vast majority of the Australian community simply did not believe that a dingo would kill a human [6,9]. Indeed, one of the jurors for the Chamberlain trial later commented in a press interview that, while the forensic evidence was a factor, the decision to acquit or convict came down to ‘“whether you believed it was a dingo (that took the child) and therefore believed Mrs Chamberlain’s story or whether you didn’t”’ [146] (p. 2). Why was the premise of this wild predatory canid attacking a human being and consuming it as food greeted with such widespread incredulity in 1980s Australian society?

At that period of time scientific knowledge of the dingo was at an early stage of development [101,102], and the public’s understanding of this animal was limited [1]. Around 86% of Australians then lived in urban centres [147]. Most had probably never seen a dingo before [6,148]. For the first time in the country’s modern history the dingo was a topic of national conversation [15,116,138,149]. One authority observed that ‘The two inquests were almost solely concerned with the capacities of dingoes to kill infants’, and noted that in popular belief it was the dingo that was on trial, not Lindy [7] (p. 135). It was also pointed out that ‘the controversy that raged over the dingo story … revolved around the believability of whether a dingo could, or would, take a baby’ [116] (p. 61). Concerning the ‘could’, there was much debate about technical matters, such as whether a dingo was physically capable of performing the deed on the grounds that these canids lacked the strength to carry a 4.3 kg baby (they do not); their jaws could not open wide enough to encompass an infant’s cranium (they can); or they could not remove a baby’s clothing without tearing it to shreds (they can) [6,10]. With regards to the ‘would’, the implicit assumption in the public arena—judging from the gossip and black humour in oral circulation at the time [138]—was that dingoes ‘simply do not act that way towards humans’ [116] (p. 63). As one of the foremost commentators on the case, John Bryson, put it: ‘Remember the rumours? The first, and most decisive for the rest of the story, was “Dingoes don’t behave like that”’ [10] (p. 277). Where did this popular belief about dingo behaviour come from?

The most commonly held cultural image of the dingo at this time was of a cunning pest animal, an unruly marauder that hunted domestic sheep and calves with great avidity [77,78]. Yet, despite the dingo’s reputation for killing livestock—often with what was regarded to be wanton cruelty [80]—it was also perceived by the public to be, in essence, ‘just a dog’; an untamed one, but nevertheless a sort of native breed of domestic canine (*C. familiaris*) that was closer in disposition to a dog than a wild wolf [43]. This belief was no doubt influenced by the external physical features of dingoes, which are indeed very dog-like (at 15–16 kg dingoes are about the same size as a medium-bodied dog breed), and also possibly by the longstanding practice of keeping dingoes as pets [48,150]. Another factor might have been the notion that two of Australia’s iconic herding dog breeds have dingo ancestry, the kelpie and the blue heeler [48] (in the case of the kelpie this may be a myth [151]). However, foremost was the commonly held perspective that dingoes were timid and elusive canids that were extremely shy of humans and had never before been known to regard us as potential prey [48].

This ambivalence towards dingoes was deeply entrenched in European-Australian cultural mythology in the 1980s [14,137,138]. It was even claimed that one of the first police investigators assigned to the Chamberlain case rejected the ‘dingo baby’ theory out-of-hand on this basis: ‘“Not a chance … Never happened before [a dingo attacking a human]. There’s a fact you can’t beat. Never ever happened”’, he allegedly pronounced in an off-the-record conversation [6] (p. 79). Lindy Chamberlain, of course, had much the same view of dingoes right up until the moment of Azaria’s disappearance:

As soon as I reached the front of the tent, I could see the blankets scattered. Instinct told me that she wasn’t there, the dog had her, but my head told me it wasn’t possible. Dingoes don’t do such things, and this was, you know, just beyond the realms of reason [6] (p. 155)

In the early 1980s stories about dingoes attacking or menacing people did exist within the Australian community. The Chamberlains received letters from the public about such experiences ‘in earlier settlement days’, including stories of schoolchildren being tailed daily by dingoes, and a couple ‘mustering cattle and camping overnight … waking up and finding a dingo by the … cot that their two-year-old slept in’ [9] (p. 91). One elderly rural dweller wrote that as a child she had ‘always been put to sleep in “a wagonette” away from the dingoes’ [152] (p. 231). Such anecdotal accounts were evidently so little known to the public, however, that it was possible for the Crown to submit that it was ‘general knowledge’ and ‘common sense’ that dingoes had never attacked a human in Australia’s history [9] (p. 267). (Notably, it later emerged that several years before the trial one juror had a close call with a wild dingo: it apparently entered her backyard on the outskirts of Alice Springs and tried to snatch some food her toddler was eating, grazing his cheek and neck; the dingo then aggressively resisted her efforts to ‘shoo’ it away, biting and ripping her jeans [153]).

Subsequently, the way most Australians think about dingoes has changed dramatically [11,154]. The well-documented attacks on K’gari (see Appendix B) instigated a transformation in modern cultural attitudes towards these canids [17,18]. In light of the tragic death of Clinton Gage, and other high-profile attacks since then, a 2012 coronial inquiry concluded that a dingo took Azaria, fully exonerating the Chamberlains [16,155]. It is now recognised that dingoes do represent a legitimate danger to humans, especially children, under certain circumstances [11,155].

## Appendix B. Human–Dingo Conflict on K’gari

There have been recent incidents involving wild dingoes engaging in what seem to be unprovoked, predatory attacks against humans on K’gari [17,18]. A large sand island (1655 km^2^) lying off the coast of south-eastern Queensland, K’gari is a World Heritage Area and a popular family holiday destination that attracts hundreds of thousands of visitors each year. Tourists typically bring 4WDs and fish and camp outdoors and visit the pristine beaches, forests, lakes, and other wilderness areas. The island is also host to a population of free-ranging dingoes (~160–260 individuals) that is commonly held to be genetically ‘pure’ [37]. Many tourists are drawn to K’gari to observe these dingoes ‘in the wild’ [154]. Some are blasé about laws prohibiting humans from feeding or interacting with dingoes, endangering themselves and the dingoes, which are often euthanised by rangers if they harm or threaten people [37].

Stories about a rise in dingo attacks had been circulating among K’gari residents in the early 1990s [43,154]. However, dingo-human conflict was not the focus of national news until March 1997, when a pair of dingoes attacked five-year-old Andrew Bartram while he was playing hide-and-seek at a barbecue area in the Eurong Beach Resort [156]. The dingoes pounced on the child and began dragging him into the scrub, before his mother, alerted by his screams, scared them away. The boy suffered multiple wounds to his legs and groin [156]. A more widely publicised incident took place the following year in early April, with a dingo seizing 14-month-old Kasey Rowles by the shoulder and dragging her 1–2 m away from her family’s Waddy Point campsite, before her father intervened [157]. Then, on the morning of 30 April 2001, a nine-year-old boy, Clinton Gage, who had been camping with his family near the same locality, was killed in a dingo attack [17,18]. At the time of the incident Clinton was walking along a track with a boy of similar age, when two dingoes began to follow them [17]. Investigators concluded that Clinton probably panicked and started to run ahead, but tripped and fell, while his friend kept walking. When the other boy looked back, he saw the dingoes standing over Clinton—the last time he was seen alive. About 20–30 min later, Clinton’s father and six-year-old brother, who had been searching for Clinton, found his body. He had received severe injuries to his head and neck, upper body, abdomen, thighs and groin, including a severed femoral artery [43] (p. 14). The pathologist who examined the boy’s body formed the impression the dingo attack had a predatory motivation [43]. While Clinton’s father retrieved his remains the two dingoes determinedly attacked his brother [17]. According to one source: ‘The boys’ father “shooed” the most aggressive dingo away. As they made their way back to the campground the dingoes kept harassing the younger boy, especially if he moved too far in front of his father’ [17] (p. 136).

Multiple dingo attacks and negative ‘incidents’ have been documented on K’gari since 2001 [17,18]. A notable event took place in April 2019, with a dingo apparently dragging a sleeping 14-month-old boy, Hunter Allister, from his family’s camper trailer [158]. This case was widely reported in the media, often in a sensationalised manner [32]. According to the parents’ testimony, a dingo entered their camper trailer late at night by jumping up on to it and popping open a clip on the canvas access flap [158]. It then seized the child by the back of the head, pulled him out of the camper trailer onto the ground, and dragged him several metres into the scrub [158]. His father, alerted by the child’s screams, ran after the dingo shouting, causing it to drop the boy, who was left with a fractured skull and deep lacerations to his head [158]. More recently, young children were attacked by dingoes at Orchid Beach in February, April and May of 2021 [159,160,161]. One child, a two-year-old boy, wandered off alone from his family’s holiday house at around 7 am and was bitten on the neck, shoulders, buttocks, thigh, and head by a solitary dingo before bystanders chased it away [159]. The paramedic who treated the child said he was ‘“very lucky to still be alive”’ [159].

The accepted thinking among scientists and some members of the K’gari community (but cf. [17]) is that elements within the island’s dingo population only became potentially dangerous in the early 1990s owing to the deleterious effects of the tourist boom [162]. In support of this argument, it is pointed out that only small numbers of white settlers (mostly loggers) had inhabited the island in colonial times and throughout most of the 20th century [163], and it is inferred that this tiny European community lived harmoniously with the dingoes [162] (but see below). Moreover, in 1970 there were still only around 5000 people visiting K’gari per year [164]. By 1986/1987, however, visitor numbers had increased to 164,000 per year, and at the turn of the century the island was drawing 312,500 tourists annually [165] (p. 14). The prevailing view is that this major influx of visitors has profoundly altered dingo behaviour, leading to the habituation of dingoes that frequent tourist areas and prompting the recent spate of predatory attacks [162,164]. Some K’gari residents also claim that changes introduced by the national park authority since the 1990s may be indirectly responsible for the attacks, by placing the wild dingoes under increased food stress; in particular, culling the island’s feral horse population, on which the free-ranging canids had long preyed, and fencing-off the rubbish dumps at which dingoes had regularly scavenged food waste [43,53,162].

On the contrary, there seem to have been ‘problem’ dingoes on K’gari long before the 1990s. Indeed, one of the earliest known possible dingo attacks in Australia occurred nearly two centuries ago on this island (Table A1). In 1836 a small party of British castaways was marooned on K’gari (then completely uninhabited by Europeans), and one of them, the second officer John Baxter, aged around 23 at the time, later reported that: ‘He was once attacked and bitten by a wild [dingo]’ [166] (p. 66). Furthermore, an ‘old timer’ who began work as a bullock-driver on the island in 1910 told of a local hunter who was attacked by dingoes [162] (p. 163) (see Table A1 for more details). Two other timber-getters recalled that in the early 1900s dingoes regularly hunted and ate their domestic dogs, brazenly approaching their camps to attack or lure the animals away [162] (pp. 164–165). In June 1924, newspapers also reported that large packs of fierce dingoes were roaming the island [167,168,169], and that one group of dingoes had attacked a K’gari lighthouse keeper (Table A1). Consequently, one article stated that ‘Boating parties have been advised not to land on unfrequented portions of the island unless armed with rifles and plenty of ammunition’ [169] (p. 4). A decade later a visitor described dingoes that were seemingly unafraid of people: ‘Dingoes, which are very plentiful, seem cheekier (i.e., bolder, less fearful) than the mainland dingo, and came quite close to the camp in day-time’ [170] (p. 7). It was also reported at this time that ‘Dingoes infest the island and pry close to the camps in search of scraps’ [171] (p. 6). Yet another early sightseer commented that the K’gari dingoes were reputed locally to be dangerous: ‘We heard stories of … dingoes which drove a timber-getter into the sea with his bicycle’ [172] (p. 29). In 1950, the occasional visitor to the island still reported that ‘Dingoes … often come round camps at night time’ [173] (p. 3). These historical accounts seem to imply that negative interactions between humans and K’gari dingoes are not a recent phenomenon.

## Appendix C. The Disappearance of Willie Gesch

On 14 December 1880 a two-year-old boy, William Samuel ‘Willie’ Gesch, went missing from his family’s home on a selection at Munna Creek, near Miva, in the Gympie district of southeast Queensland (Table A1). It is worth examining this 122-year-old ‘cold case’ in detail. Up until now very little information has been published on this account of the loss of a child that was attributed at the time to dingo predation. It is also of historical value in of itself, given it was discussed during the first coronial inquest into the disappearance of Azaria Chamberlain [6], as noted above.

In the first report of this incident, a brief note published in a local newspaper, the *Maryborough Chronicle*, on 21 December 1880, it was stated that Willie’s mother Isabella had left him unsupervised on the verandah for about 10 min while she drove some cattle out of a paddock [174]. When Isabella returned Willie was gone, and a four-day search of the area by a Constable Dawson, and neighbouring selectors, revealed no trace of him [174]. The article suggested that Willie’s disappearance took place in ‘broad daylight’ [174] (p. 2). A short note published the following day in another provincial newspaper, the *Gympie Times*, reproduced this same story almost verbatim, although it added a new element to the narrative: ‘When [Willie’s mother] had got to the scrub, near the selection, she heard a scream coming from the direction of the house, and returning at once missed the child’ [175] (p. 3). Whether this was an embellishment is unknown. The article did allude to information provided by a local informant, however [175].

Both articles noted that Willie was wearing light clothing at the time, had no shoes on, and had a sore foot [174,175]. Willie’s mother and father therefore believed, according to the media reports, that he could not have strayed from the house, and consequently, ‘the bereaved parents were compelled to resign themselves to the terrible belief that the child had been stolen off the veranda by dingoes, and eaten’ [174] (p. 2). In support of the dingo predation theory, the journalist for the *Maryborough Chronicle* wrote that, two days before Willie’s disappearance, ‘unusually bold and ravenous’ dingoes had seized pigs and even a large goat on neighbouring farms, ‘in every instance bearing their prey away at a rapid rate’ [174] (p. 2). Moreover, according to the *Gympie Times*, ‘Our informant tells us that [local dingoes] have also been known to carry off young calves’ [175] (p. 3). The *Maryborough Chronicle* also stated that ‘On the day on which the child was lost, two dingoes were prowling round Gesch’s selection’ [174] (p. 2).

Willie’s father, William Gesch, registered his son’s death with the District Registrar of Gympie on 6 March 1881 (Registration details: 1881/C/916). The cause of death box was left blank and ‘Body not recovered’ was written in the burial registry section. The correspondence records of the Gympie district police magistrate (July 1871 to December 1882; Queensland State Archives (QSA), ID PR2342713) contain no report into Willie’s disappearance, or any references at all to the child. It would also appear from a thorough search of the QSA Inquest Index that a formal inquest was never held to determine how Willie had died (J. Seccombe, pers. comm. 2022).

Isabella Gesch (*b.* 1855), an Irish immigrant, died at Miva in 1932. Her obituary notice referred to Willie’s disappearance, but there was no mention of dingoes [176]. Willie’s father (*b.* 1855), who had emigrated from Germany as a small boy, died in 1938 [177]. The child’s disappearance was still discussed among older residents of the Miva district in the late 1950s [178] (p. 11). Details of the incident were (and are) also preserved in Gesch family lore. Regarding the latter, new information on Willie’s disappearance was provided in a letter from Willie’s cousin, G.E. Gesch of Urangan, published in the *Maryborough Chronicle* in 1954 [179]. This was evidently Willie’s first cousin, George Edward Gesch (1881–1970), whose father, August Gesch, William’s older brother, settled in Miva with his family at around the time of Willie’s disappearance [178]. The letter stated that no traces were ever found of the child, ‘not so much as a button’ [179] (p. 3). It also affirmed that ‘The boy had a very bad foot which prevented him walking any distance’ [179] (p. 3). Referring to the day Willie vanished, however, G.E. Gesch included a crucial new detail: ‘There was also a young baby left in the house and when the mother returned from bringing the cows she found the cot in which she left it sleeping overturned on top of the baby, and it very nearly suffocated’ [179] (p. 3). In any case, G.E. Gesch claimed that Willie’s parents never thought a dingo had taken him—on the grounds that there was a high paling fence around the dwelling, ‘the gate was securely fastened’, and a dingo would be too wary of traps to go inside a house or fence [179] (p. 3). The letter mentioned that some believed that an ‘eaglehawk’ (wedge-tailed eagle, *A. audax*) could have snatched Willie from the verandah—an idea G.E. Gesch found preposterous. Indeed, according to this letter: ‘The parents thought Johnny Campbell (the notorious aboriginal bushranger), might have taken him’ [179] (p. 3).

Further insight into the case came from Willie’s last surviving sibling. In February 1981, amid the media furore surrounding the first inquest into the disappearance of Azaria Chamberlain, a *Gympie Times* journalist interviewed Willie’s sister, Mrs. Eliza Ellen ‘Nell’ O’Keefe of Brisbane, about the possibility a dingo had taken her brother in 1880 [180]. Nell O’Keefe was born 23 years after Willie’s death, but in the article, it was implied that as a child she had been told ‘“he was not taken by a dingo”’ [180] (p. 1). The published details she provided to the reporter were broadly consistent with the content of the 1954 letter from G.E. Gesch [179], including the claim that ‘a cradle beside where her brother had been left at the house was knocked over and there was [a] fence around the house making it unlikely that a dingo could have carried a child away’ [180] (p. 1). She also mentioned the ‘eaglehawk’ theory, and further noted that some thought her brother might have wandered into the nearby swamp (there was no reference to his sore foot or feet, however). The article further alluded to a local story that Aboriginal people had kidnapped Willie [180].

Two direct descendants of William and Isabella Gesch, the granddaughters of their son Percy Gesch (*b*. 1896), and great-nieces of Nell O’Keefe, have provided new details about Willie’s disappearance based on their recollections of family oral history. According to one, Nell O’Keefe, who did not have children, was the ‘family historian’ and had an excellent memory well into her 90s (T. Levanes, pers. comm. 2022). (Many of the old Gesch family photographs and documents Nell O’Keefe had collected were destroyed in the 1974 Brisbane flood (T. Levanes, pers. comm. 2022)). She had been told by her great-aunt that on the day Willie disappeared his father William (known within the family by some members as Wilhelm) was cutting timber at Mount Urah, a major source of hoop pine about 7 km northwest of Munna Creek [178]. Furthermore, when Isabella left Willie to attend to the cattle, she was supposedly adamant she had closed the gate behind her—in any case, the family said Willie was too short to reach the latch. When Isabella returned to the house Willie was gone and the cradle in which she had left her youngest child (Willie’s baby brother Charlie Carl/Karl, *b*. 1 November 1880 [181]) was knocked over, with the baby trapped underneath. Nell O’Keefe also reportedly claimed that Willie did indeed have a sore foot and hence could not have wandered away from the house (a statement seemingly in conflict with the 1981 *Gympie Times* article). The family thought that Aboriginal people abducted Willie. William’s belief, however, was that the local Indigenous community would not have taken his son because he had a good relationship with them, and thus that ‘nomadic’ Aboriginal people—strangers to the district—were responsible (T. Levanes, pers. comm. 2022). There was also a family story that at some later stage an ‘Old Lady Krafft’ (presumably the Gesch’s Munna Creek neighbour, Mrs. Krafft, the wife of Willie’s father’s brother-in-law, Christian [178]) discovered some bones in the fork of a tree. As local Aboriginal people disposed of their children’s remains in this way, some believed these to be Willie’s bones (T. Levanes, pers. comm. 2022). Another of the great-nieces of Nell O’Keefe said that she had always been told that when Isabella returned to the home, she found that Willie was missing, his baby brother was trapped under his overturned crib, flour had been spilled all over the floor of the house, and that Aboriginal people were responsible for taking Willie (A. Sullivan, pers. comm. 2022).

It is not possible to verify the various claims by Willie’s cousin and sister about the presence of a fence around the house, and the additional family story that Isabella was sure she had closed the latch of the gate behind her. The contemporary newspaper coverage of Willie’s disappearance made no mention of a fence. Indeed, no details were provided about the structural details of the house itself, apart from the fact it had a verandah. The first homes on Munna Creek selections were typically slab huts with shingle roofs [178] (p. 13). According to family lore, Willie’s mother could no longer bear to live in the house after he disappeared, so the family relinquished their selection and moved to the Miva township (A. Sullivan, pers. comm. 2022). One of Percy Gesch’s granddaughters revisited the old Gesch selection in 1995 and at that time all that remained of the original dwelling were some posts that her father said had been a part of the goat yards (A. Sullivan, pers. comm. 2022). If there was a fence at the time of Willie’s disappearance, the gate could have been left open accidentally. In any case, even a tall fence might not have deterred a dingo: these canids are notorious escape artists and adept climbers that can easily scale >2 m-high fences [182]. Dingoes can also learn how to open gates and manipulate locks [183] (p. 221). On K’gari, some wild dingoes are ‘“quick and smart enough to learn how to open zips, locks and straps on campervans and camping equipment”’ [158].

The possibility that Willie drowned in the nearby swamp seems unlikely. This was presumably the small swamp still visible to the west of the site of the old Gesch house, and now called Tommy’s Swamp (L. Kunst, pers. comm. 2022), though it was formerly known to Munna Creek locals as Gesch’s Swamp (A. Sullivan, pers. comm. 2022). As already mentioned, the contemporary newspaper articles [174,175], G.E. Gesch’s 1954 letter [179], and the Gesch oral histories, consistently noted that the toddler had a sore foot and hence was unlikely to have wandered off. It can also be definitively concluded that the Aboriginal (Kabi) bushranger and alleged sex offender Johnny Campbell, whose Kabi name was Kagariu [184], could not have abducted Willie. Kagariu’s *modus operandi* was raiding isolated farmhouses when the ‘woman of the house’ was alone [184] (p. 144). This widely feared bushranger certainly did not kidnap Willie, however, as he was hanged for rape in Brisbane Gaol on 16 August 1880 [184], four months before the child disappeared. It is worth noting that Willie’s father William had been personally acquainted with Kagariu. In 1871 or 1872, Kagariu went into hiding with Aboriginal people on Boonara station [184], where members of the Gesch family were then employed as shepherds [185]. Betrayed to the station owners [184], Kagariu was held captive at Boonara for a week, chained to an anvil [185]. The 16-year-old shepherd William Gesch ‘had the job of feeding [Kagariu] and watching that he did not escape’ [185] (p. 3). Perhaps, long after Willie’s disappearance, this connection between Willie’s father and Kagariu was transmuted in Gesch oral history into a kidnapping-as-revenge theory (see below), despite the alleged kidnapper being dead at the time the child vanished from the Munna Creek selection.

The theory that an unknown group of Aboriginal people kidnapped Willie also seems unlikely. It was not uncommon at this early time for suspicion to fall on ‘the blacks’ when a white child inexplicably disappeared in the Bush [111,186] (see, e.g., [187]). Indeed, in 1859 two fair skinned Butchulla children, probably albinos, were forcibly removed from K’gari because they were erroneously believed to be white captives [188]. There is also a local story about Aboriginal people kidnapping a white baby from Munna Creek in the late 19th century (L. Kunst, pers. comm. 2022). These beliefs tended to revolve around the notion that whites who had harmed or offended local Aboriginal people ran the risk of their children being ‘stolen by the blacks’ as revenge [189] (p. 7), but for the most part there appears to be little credence to them [111] (p. 48). Interestingly, however, in some areas this aspect of settler folklore contained the belief that the children lost to vengeful Indigenous abductors did not meet a fate worse than death. In fact, according to one early chronicler, ‘a white child taken by the blacks was treated with “savage kindness” and never subjected to cruelty at the hands of those it had fallen into’ [189] (p. 7). Some settlers believed the youngsters were taken to a remote place far from European eyes, where they lived peacefully as an adopted member of the Aboriginal community [189]. It may be the case, therefore, that one or both of Willie’s grieving parents did genuinely come to believe at some later stage that he had been abducted by Aboriginal people—instead of the horrifying notion he was dragged off and eaten by a wild dingo—allowing them to retain the hope that their firstborn child was still alive somewhere out in the Bush.

In sum, the information available on the disappearance of Willie Gesch is inconclusive. It is tempting to draw comparisons between this incident at Munna Creek in 1880 and the ‘lifting’ of baby Azaria Chamberlain from a tent a century later, and perhaps even the 2019 case on K’gari where a dingo entered a camper trailer and dragged away a sleeping 14-month-old child [158]. It is unfortunate, however, that such limited detail is available about the nature of the Munna Creek ‘crime scene’ in the contemporary newspaper reportage and other sources. If a dingo had seized Willie on the verandah, then it seems likely the child would have been bitten and severely injured in the process, suggesting there should have been at least some visible traces of blood at the scene (although the dingo’s ‘bite and shake’ method of killing often results in minimal external injuries; death comes from internal haemorrhaging [190]). An Aboriginal tracker also presumably would have been able to identify the signs of a dingo dragging away a child, but there is no mention of a tracker being involved in the search party. It is impossible to know if such evidence ever existed, and, if it did, whether it was observed and documented at the time. Despite this, the various Gesch oral histories about the baby in the upturned cradle and the flour spilt on the floor seem important. If these stories are valid, and if a wild dingo did indeed take Willie from the verandah, then it seems possible that the animal may have first entered the house after Isabella had left and torn open a flour bag to get at the contents, as well as knocking over the cradle in an attempt to snatch the baby. Alternatively, there may have been two dingo intruders (as implied by the contemporary newspaper reportage [175,176]), one causing the disturbances inside the house, the other taking Willie.

## Appendix D. Historical Accounts of Dingo Attacks

The study identified a total of 52 historical accounts of dingo attacks spanning the period between 1804 and 1928. These are summarised in Table A1 and described briefly in this section. There are several historical accounts in which humans were bitten, ‘set upon’ or otherwise attacked by a dingo or dingoes (Table A1). In accounts of dingo attacks that did not involve physical injury, the usual story is that of lone individuals being attacked or menaced in the Bush or on a farm by one or more wild dingoes and managing to narrowly escape from them, typically by climbing a tree (this was known as being ‘treed’). Most adults involved in these incidents were males, perhaps reflecting the gender imbalance of colonial times [108], but there are some accounts of dingoes attacking adult females (Table A1).

As recorded in Table A1, in almost half of cases (48.1%) the number of dingoes involved in the attack was not reported. However, in nearly one in ten cases (9.6%) a solitary dingo made the attack, while in 5.8% of cases the attack involved a pair of dingoes. In almost a quarter of cases (23.1%) the accounts described multiple dingoes (a pack or mob) attacking people, and in a further 7.7% of cases a group of 3–6 dingoes was specified as being involved. There was also one account in which a dozen or so dingoes were believed to have constituted the attacking party, and a further two accounts in which approximately 20 dingoes were involved. By contrast, analysis of human–dingo incident reports from K’gari [18] shows that, of all attacks/incidents between 2001 and 2015 (*n* = 160), most involved a solitary dingo (78%), 11% involved two dingoes, 5% involved three, and the rest (6%) involved four or five dingoes. The highest number recorded for a single incident was a group of 12 dingoes [18] (p. 151). Thus, whereas almost nine out of ten cases in modern-day K’gari involved a solitary dingo or a pair, most historical accounts described attacks by multiple dingoes, including, in two cases, very large groups (>20 individual dingoes). Further work is needed to determine if this discordance is owing to present wild dingo populations in K’gari having a different social structure to their historical counterparts in mainland Australia, or if other factors may be at play (e.g., changes in dingo social organisation and pack structure that have occurred in mainland eastern Australia since the early 20th century).

Just over half of the historical accounts (*n* = 28) are of fatal dingo attacks, the first in 1804 and the last in 1925 (Table A1). A few of these supposed fatalities reported on cases in which alcohol or drug consumption rendered adult men vulnerable to dingo attack. In total, 19 of these accounts reported the demise of children. These are of note given that the only two documented dingo-related fatalities in modern times both involved children (Azaria Chamberlain in 1980 and Clinton Gage in 2001), as previously discussed. These historical accounts typically described small children (average age ~4 years) going missing in the Bush, usually from shepherds’ huts or selectors’ homes on small land holdings (see, e.g., the case of Willie Gesch (Appendix C)). They were either found later partly consumed, or no trace of them was ever identified. The earliest such report was published in 1804 [191] (Table A1). The *Sydney Gazette* claimed that a two- or three-year old boy wandered away from a farm at Prospect (Parramatta area) and was lost overnight in the Bush, where he fell prey to a dingo and was ‘more than half devoured’ [191] (p. 2). The details of this episode are unclear, however, as a few weeks later it was briefly reported in the same paper that the cause of death was, in (apparent) fact, drowning [192]. No further information about this Prospect case has been uncovered.

Another contradictory account of a fatal dingo attack against a child comes from northern Victoria. On 10 July 1891, local newspaper *The Boort Standard* published a story under the sensational headline ‘Eaten alive by dingoes’ [193]. According to this article, on 7 July that year the young child of an unnamed married couple living on a selection in the Budgerum district was seized and carried off by a pair of dingoes. At the time of the event the father was away, and the mother was ‘engaged in domestic duties’ inside the Bush home [193] (p. 1). The child, ‘a little toddling thing’, was playing by the door and seems to have momentarily strayed outside without the mother noticing [193] (p. 1). Alerted by a ‘sharp scream from the edge of the scrub’, she ran out of the house just in time to witness ‘her child (being) dragged away by two large dogs’ [193] (p. 1). She immediately fainted from shock. After regaining consciousness, the panic-stricken mother ran into the dense mallee scrub after the dingoes. She was able to follow their tracks as the ground was soft from recent rains, with ‘occasional splashes of blood telling the frenzied woman all too forcibly that she was on the right track’ [193] (p. 1). The woman was unable to catch up with the dingoes, however, and when night came ‘she fell in a dead faint in the midst of the scrub’ [193] (p. 1). She found her way back to her home at dawn. When her husband returned to the house and found out what happened he went out to search for the child, but while he was able to follow his wife’s tracks, the rains had apparently erased the dingoes’. He then organised a more thorough search involving neighbouring farmers, but the child’s remains were reportedly never found [193].

This account of a predatory dingo attack is the only report identified in which it was claimed that someone actually witnessed a child being seized and carried away by a dingo. Intriguingly, however, in the 31 July edition of *The Boort Standard* a correspondent cast doubt on the validity of the event [194]. In a brief letter entitled ‘A disclaimer’, they wrote that ‘I reside in the centre of Budgerum. I know everyone in it, and such an occurrence has not happened in the district’ [194] (p. 1). Unfortunately, this person provided no further explanation that would help to shed light on this matter, and it has not been possible to find any more information about this Budgerum account. It is conceivable that this complicated story of a fatal dingo attack was simply concocted by *The Boort Standard*, a not uncommon practice at the time when reporters or editors were short on copy (for treatises on the standards of early newspaper reportage in Australia, see [29,30])—although this would seem to be a particularly egregious example. However, this study did identify one other early case of a false claim of a fatal dingo attack. In 1878 a Queensland paper reported that a lost child had been killed and eaten by a dingo [195]. It later emerged, however, that the dingo predation theory had been based on a local informant’s account that was quite obviously wrong—the child died, but a dingo was not involved [196]. In any case, it seems evident from this report that the notion that young children could be subject to dingo predation was considered plausible at this time.

It is difficult to evaluate the veracity of these historical accounts of dingo attacks owing to the nature of cause-of-death investigations at this time. When the body or scattered bones of the perished child or adult were discovered in the colonial period the most reliable method of ‘crime scene’ investigation available was the deductions of Aboriginal trackers. The common absence of formal investigation was owing to the lack of medically-trained coroners [197] (p. 55) and the widespread practice of burying bodies before they could be examined [34]. It should also be noted that civil registration of deaths was not established across all the Australian colonies until the mid-1850s [34] (p. 16). These problems were most pronounced in the more outlying districts—where the majority of the historical accounts of dingo attacks originated. It was a similar situation on goldfields, where large numbers of people congregated in remote and rural areas, deaths often went unreported, and official record-keeping was chaotic [34] (p. 62). Death records and registrations are also likely to have been ‘questionable’ for the tens of thousands of Chinese people and Pacific Islanders who inhabited the colonies at any given time in the latter half of the 19th century [34] (p. 66). As evident in Table A1, at least some of the historical accounts of dingo attacks seem to have involved Chinese Australian people.

Such an attitude is perhaps not unexpected, given that Australians of this time had a vastly different social attitude towards death, and especially towards childhood mortality. Indeed, until the last decades of the Victorian period around one-in-ten infants in Australia died before their first birthday [34] (pp. 83–84), most commonly of infectious diseases [198] and gastrointestinal problems [34,108]. It was not until the mid-1880s that the high rate of under-5 mortality began to decline [34,108]. Children also regularly suffered and died from an array of tragic accidents [111] (p. 13), most often drownings and burns/scalds (e.g., falling into fireplaces or vats of boiling water), but including snake bites, being thrown from (or kicked by) horses, drinking spirits, and other shocking incidents [199]. Colonial readers relished poring over the gruesome details of a ‘popular’ murder [31]. In a world where news of child deaths was almost routine, however, a report of dingoes killing a youngster apparently did not incite much controversy or debate.

## Appendix E. Cultural Attitudes towards Dingoes in Australian History

Here, the results of the second part of the study—an investigation of past cultural attitudes towards dingoes and human safety over time in Australia (See Materials and Methods)—are reported in detail. It will be argued that up until the early decades of the 20th century it was a commonly held belief that dingoes were formidable and dangerous predators that were known to attack, kill, and eat people they encountered alone in the Bush. It is further apparent that by the 1920s and 1930s this notion that dingoes were potentially ‘deadly’ had largely been replaced by the equally prevalent view that the dingo was a benign animal that would never attack a human, unless it had been provoked.

### Appendix E.1. Pre-Early 20th Century Perceptions of Dingoes and Human–Dingo Interaction

As evident from Table A1, readers of colonial newspapers in New South Wales and Queensland not uncommonly encountered dramatic stories of small children straying into the Bush and being killed and eaten by dangerous predatory dingoes. The impending horror in the image of a dingo stalking a lost child is also found in the rare stories of ‘extraordinary preservation’, where a youngster who was missing or injured in the Bush confounded expectations by surviving their ordeal. For example, when, in 1860, the small son of a shepherd who had strayed into the Bush at Murringgendon in the Darling Downs (probably Meringandan, near Toowoomba) was found alive, *The Darling Downs Gazette* pronounced that it is ‘somewhat miraculous how [the toddler] escaped the native dogs, which are very numerous on the station’ [200] (p. 3). Similarly, when the two-year-old son of a station manager in the Lachlan district wandered off into the Bush in 1865 and was found alive a few kilometres away, a newspaper reporter commented that ‘As the locality is infested by native dogs, which hunt and howl thereabouts in packs, the chances are that, if undiscovered before night, he would have been devoured’ [201] (p. 2). In the same year, an eight-year-old boy went out on horseback in the Canberra district to muster cattle, fell in with a shepherd, then fell from his horse and broke his leg: ‘But for the accidental glance of the shepherd after his youthful companion—so solitary and far from human habitation—it is clear the little fellow must have … been devoured by native dogs before morning’ [202] (p. 4). Similarly, in 1870 the *Maitland Mercury* reported on a case in which a woman was lost in the scrub with her two young children, and spent the nights ‘comforting her babe and speaking words of encouragement to her weeping boy, and fearing the while lest native dogs, attracted by the cries of her children, would come and devour them’ [203] (p. 2).

The iconic image of the vulnerable white child becoming lost in the wilderness and meeting a sad and melancholy end is a long-studied theme in the canon of Australian literature and cultural history [111,143,186]. Although it is an ‘enduring cultural myth that Europeans found the Australian environment hostile, alien [and] oppressive’ [204] (p. 41), it is certainly the case that the Bush was often represented in settler folklore as a quasi-sentient landscape that periodically swallowed up the innocent and unwary without leaving a trace, ‘almost as if they had never existed’ [186] (p. 58). The term ‘bushed’ (i.e., to be lost in the Bush) was common. According to Pierce, who has analysed the lost child narrative, and examined accounts of such cases from the latter half of the 19th century, it was the Bush itself that was held by colonists to have caused the deaths of the lost children, not any of the animals that inhabited it: ‘With rare exceptions … [i.e., snakes] … the child lost in the Australian bush is in no danger from its wild life’ [143] (p. 48).

On the contrary, it seems clear that dingo predation and the lost child motif often went hand in hand in colonial understanding. Indeed, the figure of the malevolent child-eating dingo was a pervasive feature of the lost child stories from New South Wales and Queensland. In fact, when settlers in these particular colonies were lost in the Bush it seems to have been almost unquestionably accepted that they were in grave danger of being ‘devoured by the [native] dogs’—‘a horrible end which has been the fate of so many’ [205] (p. 9). (It seems pertinent to note that the lost child cases examined by Pierce [143] mostly all derived from the latter half of the 19th century in the colony of Victoria, where dingoes had largely been exterminated by the early 1850s [112] (p. 146); moreover, the rest of his colonial accounts of missing children were from Tasmania, where dingoes have never been a part of the island fauna [1]).

It was not only children lost in the Bush who were held to be in jeopardy of ‘death by dingo’. There are also accounts of lone Bush workers or rural folk being attacked or harassed by these animals (Table A1). Such incidents were often said to occur while people were camping alone at night or moving through the Bush on foot with ‘rations’ (sides of corned beef, mutton, flour and so on) (Table A1; [206]). One writer, for example, described the time he camped with a Bush character named Jack the Native:

He said that one night when he was out by himself the [native] dogs would pull at his hair and then travel round him and try to pull off his boots. I began to be a bit afraid, and felt scared, as I had been told about them devouring men and worrying them, sometimes eating part of them and then leaving them, at a place named Frederick’s Valley, near Orange [207] (p. 10)

Another rural dweller, writing in 1881, conveyed the fear and pathos he felt when camped alone one night near Mount Drummond (South Australia), ‘with sufficient [dingoes] around me to devour me without leaving a trace … tearing the flesh from my bones’ [208] (p. 2). In another personal account, a ‘swaggie’ (itinerant Bush labourer) claimed that, while tramping from station to station in rural Queensland, ‘The wild dingoes were a terror, and gave me much trouble; many times I thought I would be devoured’ [209] (p. 26). Some settlers believed that swaggies and other itinerants or wandering vagrants that were then common fixtures in the Bush often fell prey to dingoes. As one Canberra colonist commented: ‘Many a poor tramp perished in this manner in the early days’ [106] (p. 123).

Accounts suggest rural dwellers habitually adopted customary defences against dingo attacks when alone at night in the Bush. For example, it was widely thought at this time that camping with a large fire would keep the wild dingoes away, as ‘the native dog is afraid of fire as a rule, and will seldom venture within its light’ [208] (p. 2). Indeed, as a 19th century Canberra pioneer remarked, ‘the [standard anti-dingo] practice was when in the wild bush country to keep a good fire burning all night’ [106] (p. 123). Steele Rudd’s father, the ex-convict Thomas Davis, did just this when he was encircled by dingoes at his Bush camp on the Condamine in the 1850s (as noted, he had unintentionally made his campfire beside the dingoes’ den, which contained a litter of week-old pups), making a roaring fire and continually pelting the animals with firesticks to keep them at bay [33]. (This custom of making a large fire to ward off dingoes presumably developed after the end of frontier warfare, as during the period of conflict with Aboriginal people settlers usually camped without fires to avoid detection [122] (p. 94)). Furthermore, the received wisdom was that when travelling in the Bush people might be vulnerable to dingo attacks if they were alone and on foot, and if the dingoes roamed in packs, but those on horseback were thought to be safe from harm (although dingoes were known to follow riders) [81]. An incident that was said to have taken place in South Australia before 1850 was thus deemed noteworthy, as it appeared at the time to be the first report of dingoes attacking a mounted rider [81] (Figure A1).

Stories about people being ‘treed’ or otherwise narrowly escaping dingo attacks seem to have been common enough in some areas that they entered the oral histories of rural families. For example, in 1926 a correspondent to the *Maryborough Chronicle* recounted a story his grandfather told about being ‘treed’ by wild dingoes when he was a young lad [213]. He claimed he had been lost in the Bush and when darkness fell, he was attacked at his fireside by numerous dingoes. He did what many rural dwellers at this time had evidently been taught to do in the course of their bushcraft education (i.e., shouted at the dingoes and threw burning sticks at them), but this did not deter the animals, so he quickly climbed a tree. According to his grandson’s testimony, ‘The dingoes stayed there snapping at the branches, and making an awful row till day broke’ [213] (p. 3). Another Maryborough correspondent told a similar story about a frightening incident her grandmother claimed she had experienced as a small child [214]. She had supposedly been walking home alone on a Bush track when she was confronted by a group of wild dingoes:

What was she to do? She could not fight them, single handed, and neither could she turn away from them, for they would soon overtake her and then attack her. My intrepid granny ran to the nearest tall tree and climbed to the highest branch. The enraged dingoes were by now at the foot of the tree, howling and springing upwards; but granny was just out of their reach [214] (p. 6)

Dingo attacks against humans also seem to have been a source of narrative fiction and other early Australian literature. For example, a story published in an illustrated newspaper in 1870 told a farfetched tale of a lone hunter being attacked by a large pack of dingoes and then engaging in a desperate hand-to-hand battle with an Aboriginal warrior [215]. This racist adventure fantasy was surely a work of fiction, but it contained recognisable themes associated with contemporary accounts of dingo attacks: a lone rural dweller, encircled by dingoes at a Bush camp, makes a large fire to keep away the encroaching animals, then takes refuge in a tree with dingoes slavering below [215].

Another tale portrayed contemporary ideas about the predatory nature of wild dingoes, and their alleged propensity to devour lost Bush children, in an innovative way [211]. Published in 1914, this whimsical piece was told from the perspective of Warrigal, a young male dingo who had been reared by settlers on a New England station before being lured back to the Bush by a wild female [211]. The wayward Warrigal soon became an ‘outlaw’ with a bounty on his head, but life with humans had instilled a measure of dog-like compassion (e.g., he killed the settlers’ sheep; but, unlike a truly wild dingo, never ‘for fun’). The story ends with the erstwhile human pet finding a selector’s child who was lost in the scrub and cornered by a pack of hungry wild dingoes (Figure A1). Despite being relentlessly pursued by vengeful sheep farmers, Warrigal drove away his ‘savage kindred’, saving the little girl from imminent death [211] (p. 49).

Dingo attacks were also the subject of Bush poetry: for example, in this offering about a Bushman’s past travails:

He told me of many ups and downs On this dreary track at night, When the native dogs like packs of hounds, Had attacked him left and right [216] (p. 3)

Another Bush poet, writing in 1883, composed a plaintive lament about the demise of James ‘Jimmy’ Bole, a toddler who had vanished that year in the Bush in the Mt Gambier district and was presumed eaten by dingoes (Table A1):

In fancy I hear his last terrified cry, As he wakens to catch the wild dingoe’s [*sic*] eyes. Sometimes death’s a blessing and so it was here, For it ended all sufferings, dispelled all fear… [217] (p. 4)

In a similar vein was a story published in 1920 about a dingo attacking a settler woman and baby [212] (Figure A1). The piece appeared in a literary magazine for school children and was set long ago in a remote part of New South Wales. It told of a woman who was taking the baby of a neighbouring selector back to her home late one night, alone and on foot. Earlier, the infant’s father had given the woman a waddy (Indigenous fighting club) as protection from the local wild dingoes. She brushed off his concerns, though she prudently took the weapon. She was soon glad that she had, as out on the Bush track the baby’s cries attracted a group of wild dingoes. One large dingo determinedly stalked the woman and lunged at her repeatedly, trying to seize the infant from her arms, but each time she fought it off with blows from the waddy [212] (Figure A1). Though (presumably) fictional, this story of a heroic Bushwoman shows that the notion of small children falling prey to dingoes was still a familiar trope at this time.

Among the deepest fears of rural colonists was the idea that their child might stray into the trackless Bush and disappear forever [111,143,186]. However, scholars might have overlooked the possibility that a dread of dingoes also loomed large in the worlds of rural-dwelling parents, especially those at the farthest boundaries of settlement. For people living an isolated existence in the slab houses or bark shacks of small-farming areas, in shepherd’s huts and so on, the fear of dingoes taking a child might have been prevalent. It is certainly the case that the sound of dingoes howling in the darkness was an unsettling experience for many rural settlers [77] (Figure A2). One observer described the dingo’s howl as ‘inexpressibly weird’ [218] (p. 18). Another wrote: ‘It is a wail like a lost spirit in torment—a long guttural “*O—oh!*”—the worst cry in the bush’ [66] (p. 10). It is interesting to speculate whether some Bush people may also have perceived a salient warning in this vocalisation—a reminder to keep a close eye on small children.

Conversely, there also appears to have been a belief among at least some settlers that wild dingoes could be lured from the Bush by the cries of a baby or small child. In a widely publicised incident that occurred in country Victoria in 1865, Elizabeth Young murdered her 11-month-old grandson [219]. According to a contemporary newspaper report: ‘When disarmed … the unfortunate woman assigned as a reason for the barbarous act, that if she had not killed the child the dogs in the bush would have devoured it’ [219] (p. 2). Elizabeth Young obviously suffered from a serious psychiatric disorder: indeed, she had previously tried to murder her adult son, and she believed people were engaged in a complicated plot to poison her. It can be speculated, however, that the infant had been crying excessively prior to the murder, and that the killing was partly motivated by a contemporary folk belief that the sound would attract the wild dingoes. The above reports indicate that such an excuse was at least conceivable at the time. (This must also open the door to the possibility that ‘death by dingo’ was a cover story for child murder in some cases, notwithstanding the uncomfortable implications this raises in relation to the Chamberlain case and Lindy’s false conviction [6,9]).

Up until the early decades of the 20th century Australians also not uncommonly read and heard stories of dingoes consuming the bodies of people who had died of thirst or exposure in the Bush (Figure A3), including most famously the ill-fated explorers Robert O’Hara Burke and William Wills in 1861 (Figure A4) [220] (p. 3). In a similar vein, many colonists evidently had a morbid fear of dingoes violating the sanctity of the grave by digging up and feeding on improperly buried corpses (e.g., [221,222]). For example, a correspondent to a Northern Territory newspaper implored local authorities to fence off a rural cemetery in order to ‘keep out the wolfish native dogs, who are always sniffing about waiting for whom they may devour’ [223] (p. 2). A Canberra settler even arranged to have his coffin encased with stones to prevent dingoes from defiling his remains [224]. This notion of ‘grave-robbing’ dingoes is also referenced in the chorus of the popular Australian folk song, ‘The Dying Stockman’, as recorded in this version by the renowned Bush poet A.B. ‘Banjo’ Paterson:

Wrap me up with my stockwhip and blanket, And bury me deep down below, Where the dingoes and crows can’t molest me, In the shade where the coolibahs grow [225] (p. 66)

The fourth verse of this traditional Bush ballad also alluded to the deathly figure of the wild dingo, howling and lurking nearby:

Hark! there’s the wail of a dingo, Watchful and weird—I must go, For it tolls the death-knell of the stockman From the gloom of the scrub down below [225] (p. 67)

These notions of the connection between death, dingoes and the afterlife had a long pedigree in Australian colonial folklore. For instance, the old ex-convict Joseph ‘Smasher’ Smith claimed that in the late 18th century he had been consigned to the public farm at Toongabbie [75], a place of secondary punishment [226] (p. 95) where the corpses of convicts who were worked or flogged to death, according to ‘Smasher’, were dumped in an open pit, and ‘“The native dogs used to come down at night and fight and howl in packs, gnawing the poor dead bodies”’ [75] (p. 42).

Finally, there are a number of references to fatal dingo attacks that had supposedly occurred, but that contained no information on the date of the incident, name of the victim, specific geographic location or other identifying details (see, e.g., [207]). For instance, in one dramatic narrative a writer recalled his thoughts when he was menaced by dingoes while camped alone in the Bush: ‘I particularly recollected the case of a ration carrier somewhere in New England, who had been attacked and devoured by the dingoes, rations and all’ [227] (p. 233). In another story of this ilk, a correspondent to the *Sydney Morning Herald*, ‘the authoritative newspaper of NSW’ [28] (p. 15), claimed that ‘many years ago’ during a Gippsland winter a party of seven miners traveling in mountainous country was stalked by dingoes and then attacked at their overnight camp [228] (p. 11). Five miners, though badly mauled, escaped by climbing trees, while all that remained of the other two after the dingoes had left were ‘a few scraps of clothing and some of the larger bones”’ [228] (p. 11). These stories have the flavour of campfire yarns (i.e., tall tales), but they offer hints at the extent to which the ‘deadly dingo’ trope pervaded early rural folklore and belief in parts of Australia.

It bears pointing out that the notion that dingoes sometimes attack humans, while commonplace in the cultural world of 19th century colonists, was not a unanimously accepted fact. For example, a diarist who established an overland route from Adelaide to the Victorian goldfields in the early 1850s made the following entry during this journey, after being kept awake all night by dingoes howling and prowling around the camp: ‘“These animals are perfectly harmless, and have never been known to attack anyone”’ [75] (p. 379). In 1891 a local Victorian paper also remarked that the dingo ‘has up till now been thought averse to attacking human beings’ [193] (p. 1) (for a similar editorial from Queensland, see [229]). In the early 20th century, some commentators on rural life also cast doubt on the idea that wild dingoes included humans among their prey species. For example, writing on the eve of World War I, a correspondent to the *Sydney Mail* (the weekly of the *Sydney Morning Herald*) pronounced that a Bush dingo would not attack a human ‘as long as one is able to sit up and make some kind of a noise’ [56] (p. 10). The caveat in this statement nevertheless clearly shows that it was still believed that when sleeping (or unconscious) one was vulnerable to dingo predation.

It is not contended that settlers lived in mortal fear of being killed and consumed by dingoes—although some might have, and anxiety about the loss of a child through dingo predation may have been commonplace in rural areas. A dingo attack seems to have been just one of the perceived dangers thrown up by the vicissitudes of the Bush; indeed, it apparently paled in comparison with the dread of flood, drought and bushfire, and being speared by Aboriginal warriors during the frontier wars [204]. Nevertheless, written sources from the late 19th century in particular suggest that the notion of dingo predation gripped the public imagination. This may have been because the dingo was the only wild Bush animal (apart from crocodiles in the tropics) that settlers believed could subject a person to ‘a worse fate than Jezebel—namely, to be eaten *alive*’ [227] (p. 233; emphasis in original). In fact, the historical sources can be read to suggest that death by dingo was regarded at this time as the Antipodean equivalent of white colonists being killed by lions in Africa or tigers in Asia. It had none of the powerful exoticism, however, of being done-in by such awe-inspiring big cats, ‘royal’ beasts around which a special mystique had developed in colonial expatriate culture [230]. By contrast, there was little romance in the quintessentially Australian image of a lost child or lone Bush traveller being torn apart and devoured by wild *dogs*—creatures reviled by settlers as sly and verminous thieves [78].

### Appendix E.2. A Shift in Thinking in the Early 20th Century

A pronounced change in how Australians viewed dingoes and the issue of human safety first becomes plainly evident in the written record in the 1920s and 1930s. According to an article in the *Sydney Mail*, for example, by the late 1920s the question of whether dingoes would attack humans was already ‘a fertile source of argument amongst bushmen’ [231] (p. 18). As previously mentioned, stories were still circulating at this time of colonists being attacked, killed and eaten by packs of dingoes or of having to climb trees to evade them (see also [206,232]). However, these accounts were now more commonly discounted as tall tales or regarded with wry amusement, and, in some cases, ill-disguised contempt. For example, in 1928 a correspondent to *The Bulletin* (hereafter: the *Bulletin*)—an influential Sydney-based periodical then known as the Bushman’s Bible because of its popularity among rural readers from country towns to the far ‘Outback’ [122]—claimed that ‘If a sober man was treed all night by wild dogs … it is safe to say that the dogs were not dingoes’ [233] (p. 23). Another Bush lore authority, writing under the pseudonym ‘Drover’, declared to the *Sydney Mail* that ‘There is no authentic case where dingoes have attacked a human being’ [234] (p. 38). Referring to the (evidently then-bygone) stories of rural folk being ‘treed’ by dingoes, this writer irreverently suggested that ‘much anxiety would have been saved had they only known that the dingoes were much more afraid than was the tree-climber’ [234] (p. 38). ‘Drover’ also expressed scepticism about the older notion that dingoes would attack humans if they outnumbered them, opining that ‘when running in packs dingoes do not become any gamer as far as their natural fear of human beings is concerned’ [234] (p. 38). In sum, there is no doubt that this correspondent emphatically rejected the ‘deadly dingo’ trope: ‘The idea of dingoes attacking human beings is a fallacy and one that bushmen grin at’ [234] (p. 38).

It is evident that such views were not confined to the popular southern mastheads and magazines. For example, in 1928 a correspondent to a local Rockhampton newspaper, *The Capricornian*, which reflected a more rural northern readership, claimed that ‘In no case have I known a bushman attacked by the dingo’ [235] (p. 9). Similarly, in a 1933 article entitled ‘Not man-killer: Australian Dingo’, a journalist for Queensland’s major newspaper, Brisbane-based *The Courier Mail*, declared that:

Mr. Norman Bourke, president of the United Graziers’ Association, said he knew the habits of dingoes very well, but he had never heard of a case of human beings being attacked by the dogs. It was a million to one chance against it [128] (p. 16)

The *Sunday Mail* in Brisbane also pronounced that ‘The loneliest bushman doesn’t fear the dingo. He may be camped out where a score of wild dogs are howling about the camp all night, but he knows there is no fear of attack’ [236] (p. 11). It was speculated by some contemporary authorities on rural life that dingoes *might* attack a defenceless person who was near to death, but the general consensus at this time was there was no record of this ever happening [128].

By the late 1920s the sundry reports of dingo attacks from the previous century were not just receding into history, they were being excised from it. For example, in 1928 a correspondent to Canberra-based newspaper the *Great Southern Leader* declared in an acerbic letter that dingoes were once referred to with dread as formidable human-killers, from which ‘[m]any hair breadth escapes … have been recorded from time to time’, but *only* in ‘old school books of England and America’ [237] (p. 8). This writer forcefully asserted that contemporary Australians had always rejected this ‘incorrect and exaggerated description of the dingo’ [237] (p. 8). Clearly, whoever wrote this piece had been unaware of, disregarded, or downplayed the frequency with which stories of dingo attacks circulated in colonial Australia (including in the early print media), and the solemnity with which these stories were then greeted.

Most tellingly, by the 1920s and 1930s people in Australia were apparently also much less prone to imagine that children were vulnerable to dingo predation, even when lost deep in the habitat of the ‘native dogs’. For example, a London-based Australian wrote derisively of the ‘comic ignorance’ of prospective English migrants, who asked ‘“would the dingoes attack children?”’ [238] (p. 8). Writing in 1936, a correspondent to *The Queenslander* also claimed that ‘Scores of children, many mere toddlers of two or three years of age, have been lost in the bush, wandering for days and sleeping at night in dingo haunts, but none has been molested’ [239] (p. 2). One writer asserted, in stark contrast to colonial images, that: ‘A lone dingo would run away from a child’ [234] (p. 38). Correspondents to a northern New South Wales newspaper even debated whether a wild dingo would have the audacity to attack a domestic dog [240].

In the late 1920s it was still possible to encounter occasional stories in the print media about dingo attacks (Table A1). A notable example is this letter to the editor of Sydney tabloid *The World’s News*, which stated that: ‘Another case I heard of was that of a child left sleeping under a tree, while the mother was helping her husband burn off. The dingo was dragging the child off when its cries attracted the parents’ [241] (p. 8). Moreover, a correspondent to the *Bulletin*, writing under the pseudonym ‘Gouger’, and commenting on the 1928 case of George Cox (see Table A1), remarked that ‘[Dingo attack victim Cox] was lucky, for there have been lone wanderers in Cape York Peninsula whose gnawed bones have been the only witnesses of a successful dingo attack’ [242] (p. 23). ‘Gouger’ was probably the popular Australian author and Bush aficionado Ion ‘Jack’ Idriess, who published in the *Bulletin* using that pen name [243].

By this time, however, rare claims of dingo attacks were, by convention, attributed to dingoes that had crossed with domestic dogs, supposedly instilling in these hybrids the genetic predisposition to attack people that was lacking in the ‘pure’ dingo (although this was not a new concept, e.g., [244]). In particular, it was widely thought that dingoes that had crossed with Alsatians—a then-novel breed believed at the time to be a wolf-dog hybrid [245]—spawned a powerful new wild canid variant [246,247]. Pastoralists feared these ‘super dingoes’ would decimate livestock and represent a serious menace to human life [248]. This matter was debated in parliament and extensively reported in major newspapers, and even inspired *Bulletin* poetry [249]. The concern was such that in 1928 German Shepherds were banned from being imported into Australia [246]. Later authorities dismissed this longstanding notion of the Alsatian-crossed ‘super dingo’ as a myth [85,86]. Events on K’gari should have put paid to the idea that genetically ‘pure’ dingoes do not attack humans [17,18], although it is still relatively common (e.g., [250]).

It was not just city dwellers who thought in this way. In 1933 one in three Australians lived in rural areas [147], and it is evident that even among country folk a distinct view had formed that wild dingoes do not represent a real threat to human life. In one rural clan, for example, the Gesch family of Miva, there is evidence for a change in the perception of dingoes that mirrored what was happening in the broader community. This family of selectors had lost their child Willie to a dingo in 1880—or so the Geschs believed at the time (if contemporary newspaper reports are to be trusted (Appendix C)). Yet, it would appear that by the time Willie Gesch’s parents died in the 1930s this family had abandoned the theory that a dingo had taken the boy, instead devising a host of alternative *post-facto* theories, such as the idea the youngster was carried off by an eagle, a dead bushranger, or a mysterious Aboriginal ‘tribe’ (Appendix C). The poignancy of these beliefs and their elaborate nature would seem to imply more than a change in this one rural family’s attitude towards dingoes, but a deeper transformation in the Australian image of this species.

At any rate, the notion that the ‘pure’ dingo does not represent a threat to humans was still evident during World War II (e.g., [251]) and the immediate post-war years. In 1948, for example, a correspondent to the *Maryborough Chronicle* carefully explained how true dingoes have an innate aversion to aggression towards humans, singling out the early ‘lucky escape’ stories for particular opprobrium: ‘There are lots of romances about men being treed by dingoes, but I was writing facts of history, not romancing’ [252] (p. 3). Elsewhere this person stated that ‘nowhere in Australian history have dingoes molested a human being, not even the smallest child’ [253] (p. 11). The writer even claimed to know of a lost girl who was *saved* by a dingo, which guided the distressed child to water [253] (p. 11). A few vague reports surfaced of doggers and other lone rural dwellers being killed by dingoes in remote areas (e.g., [251]), but these did not seem to be taken very seriously (e.g., [254]). Similarly, a media interview in 1947 with the police officer who led the search for Bartley Roebig (a young boy who went missing in the Bush near Roma, Queensland, in 1916) digressed into a long discussion of wild dingo behaviour and the innate incapacity of these canids to attack even small children who wandered helplessly lost in the scrub [255]. Ironically, however, in this same piece the anonymous newspaper reporter related an anecdotal account of a young child being attacked by a dingo, apparently without any evidence for provocation, in a wheatfield in rural New South Wales (the date of the incident was omitted) [255].

## Appendix F. Urban Wild Dingoes

The Australian public is largely unaware that dingoes have a ubiquitous presence in many peri-urban areas, especially on the fringes of cities and towns in mainland eastern Queensland [139,140,256] (Figure A5). In the latter region, urban wild dingoes commonly occur in areas where residential land adjoins bushland (e.g., national park and state forest), including in pockets of scrub that are completely surrounded by housing and buildings—for instance, dingoes have been observed within 200 m of Townsville’s central business district [256]. These free-roaming canids live permanently in peri-urban areas, denning near houses, crossing roads, traversing built-up areas, and frequenting backyards, school grounds and recreational ovals [139,140,257,258]. Urban-dwelling dingoes typically prey on small wild mammals [259,260], and most (80%) restrict their activities to times when humans are least active [256].

It is usually the case that local residents regard these ‘wild dogs’ as just feral or neglected domestic dogs (strays or unregistered pets) or dingo-dog hybrids with negligible dingo ancestry, but this is a common misconception [139]. Although hybridisation has occurred to some extent within the entire range of dingo populations [39], almost all free-ranging Australian canids (99%) have some dingo ancestry [39]. Even in eastern New South Wales, where it has long been thought that dingoes had been almost completely extirpated [83,119], most wild canids are predominately dingo from a genetic perspective [261]. In Queensland, free-roaming canids in peri-urban areas ‘remain much more genetically similar to dingoes than domestic dogs’ [52] (p. 112) (see also [262]). These canids are often phenotypically indistinguishable from dingoes (at least in the eyes of dingo researchers and managers), they behave like dingoes, eat the same things as dingoes [259,260], and they have the same breeding seasonality as dingoes (i.e., reproduce once annually over the winter) [52]. It follows that peri-urban ‘wild dogs’ are free-ranging descendants of dingoes.

There have been numerous and widespread predatory attacks against livestock and domestic pets attributed by researchers to urban wild dingoes in mainland eastern Queensland [139,140,256,257,258], along with other ‘problem’ behaviour [257,263]. A study of urban wild dingo sightings and impacts reported to seven local governments between 2000 and 2015 (*n* = 2115) showed that attacks were the dominant category (41%) [263]. In the majority of cases (77%) dingoes targeted livestock (mostly chickens [49%]), while family pets comprised around 15% of the animals attacked (predominately dogs (~85%)) and cats (~15%)) [263]. Indeed, there have been recent media reports of dingoes or dingo-like ‘wild dogs’ killing and eating domestic dogs in peri-urban areas of Brisbane [264], Gympie [265], Mackay [266], Cannon Valley [267], Sarina [268], Townsville [269] and Cairns [270]. (These incidents are not confined to Queensland: in 2011 dingoes killed 10 pet dogs over several months in Darwin [271]). Most of these attacks occurred at night, but in one case a man was walking his blue heeler in the morning when a dingo-like canid pounced on the unleashed dog and dragged it into the scrub [265].

Although attacks against humans appear to be rare [263], there have been some nonfatal ‘wild dog’ attacks since the early 2000s in mainland eastern Queensland. For instance, notable cases that took place in the southeast region in 2014–2015 included: a female veterinarian who was ‘treed’ by five ‘wild dogs’ in the Gold Coast hinterland [272]; a 72-year-old woman who was driven into the ocean by three ‘wild dogs’ at Cabarita Beach, just south of the Queensland border [273]; and a trapper who claimed he was subjected to an unprovoked attack from behind by a lone ‘wild dog’ while he was setting a trap in the Somerset district [250]). Documentation of other episodes is mostly contained within the grey literature of confidential local council ‘incident reports’ [263]. Typically, victims of attacks were unsure if the wild canids were dingoes or feral dogs, although available descriptions strongly suggest the former (e.g., [274]).

**Table A1:** Historical accounts of dingo attacks against non-Indigenous people in Australia. The table includes missing person cases involving children in which dingoes were alleged at the time to have been involved or were suspected of having been involved in the child’s disappearance (though in some cases alternative theories were entertained at the time). NSW = New South Wales; Qld = Queensland; Vic = Victoria; ACT = Australian Capital Territory; SA = South Australia; WA = Western Australia; Unkn. = unknown; M/P = multiple/pack. The titles of newspapers or books are italicised.

Year	Month/Day	Location	Age (in Years) of Person Involved	Number of Dingoes Involved	Description of Incident	Reference
1804	~8 July	Prospect, Parramatta district (NSW)	2–3	Unkn.	The *Sydney Gazette* of 22 July 1804 described the recent death of a toddler who had wandered away from the family farm and was not missed by his mother until the following day; ‘when being given to understand the infant had not been seen by any one, she rushed into the Bush attended by several friends and neighbours, and about three miles distant from the house near to a pool of water, found the scattered remains of the boy, whose body had apparently become a prey to native dogs, and was more than half devoured’ [191] (p. 2). In a subsequent report on this case, however, published 5 August, the *Sydney Gazette* recanted the dingo theory: ‘The report that prevailed about Parramatta, and by which we were misled, stating that the unfortunate infant that strayed from Prospect had been partly devoured by native dogs, was unfounded; but we are extremely sorry to learn that the only fallibility of that account consisted in the circumstances of its death: The body of the little creature having been last week found at the verge of a pond in the neighbourhood of Toongabbee [Toongabbie], where it had doubtless perished through fatigue and want’ [192] (p. 2). No further details about this incident have been forthcoming.	[191,192]
1805	February	Botany Bay (NSW)	Adult	2	According to a florid account in the *Sydney Gazette*, an unnamed man claimed he was attacked by a pair of dingoes while walking alone in the Bush near the Botany Bay settlement. He hurriedly climbed a tree to escape. The dingoes, ‘after imposing an embargo of seven or eight hours by their vexatious presence, at daylight forsook their prisoner to a train of unenviable reflections’ [275] (p. 8).	[275]
1815	August	Nepean district (NSW)	2	Unkn.	The two-year-old daughter of John and Ann Andrews (the child’s name was omitted from the contemporary reports) disappeared from the family’s farm on 11 August 1815; no trace of her was found after an initial search of the surrounding bushland, leading to the presumption that she had been ‘carried off by native dogs’ [276] (p. 2). A subsequent report stated that her body was found 17 days later ‘in an almost impenetrable scrub, at the distance of a mile and a half from the spot where she had been last seen. A part of the body had been devoured, apparently by native dogs, by which it was most probable the unfortunate child had been drawn into the scrub, after a death from cold or famine’ [277] (p. 2). The *Sydney Gazette* contained reports of dingoes causing widespread stock destruction in the Nepean settlement in 1814 [79].	[276,277]
1836	Unkn.	K’gari (Qld)	Adult (23 years of age)	1	British castaway John Baxter reportedly claimed that while shipwrecked on the island of K’gari in 1836: ‘He was … attacked and bitten by a wild (dingo)’ [166] (p. 66). No further details are available, and no insight was offered at the time into the dingo’s motivation.	[166]
1841	August or September	Namoi River (NSW)	~3	Unkn.	The three-year-old child (unknown sex) of Patrick Taigh, a stockman or shepherd at the Namoi River Station, disappeared in the Bush. The searchers concluded that the child was ‘devoured by the native dogs’ [278] (p. 3).	[278]
1843	February	Hunter River district (NSW)	~2	Unkn.	A small boy, first name unknown, son of clearing leaser James Gill, went missing from his family’s home in the Bush close to a nearby township. His tracks could be traced for some distance, but his body was never found; ‘There are divers [sic] opinions as to the manner in which the child met its end, some supposing that native dogs have destroyed it’ [279] (p. 2).	[279]
1844	Unkn.	Wollombi area, Hunter Valley (NSW)	Adult	~12	In a colourful first-hand account, a writer claimed that while on a solo hunting trip in remote bushland he or she was attacked one evening by about a dozen dingoes, which surrounded them snarling and snapping. The writer escaped the dingoes by climbing a tree that had fallen at an angle against another. One of the dingoes tried to follow the person up the tree, so they shot it, and then fired again into their midst. At this time the writer’s hunting dog came to their aid, and, after another shot, the dingoes retreated.	[227]
1844	~November	Melbourne area (Vic)	Adult	Unkn.	A man named Guise rode his horse into the Bush while suffering the effects of advanced alcoholism, and disappeared. His body was found later, partly eaten by dingoes. The assumption was that in his incapacitated state he had been killed and partially consumed by dingoes.	[280]
~1845	Unkn.	Mount Tennant, Tharwa (ACT)	~12	Unkn.	A fatal dingo attack was supposed to have taken place in about 1845 near Mount Tennant, Tharwa. Details of the case were preserved for decades in the oral history of the local rural community prior to being published for the first time by Canberra journalist John Gale (1831–1929), an early resident of the Queanbeyan-Canberra area, and pastoralist Samuel Shumack (1850–1940). The written accounts of Gale [281] (p. 112) and Shumack [106] (p. 123) suggested that an unnamed 12-year-old girl, daughter of a family that worked on Cuppacumbalong station, ran away from home following a disagreement. While hiding in the scrub overnight she was reportedly attacked, killed, and partly eaten by dingoes. Her remains were found the next morning. No newspaper articles or other contemporary accounts that referred to this incident have been identified.	[106] (p. 123); [281] (p. 112)
~1850	Unkn.	Para River (SA)	Adult	~20	An unnamed shepherds’ hutkeeper, travelling on foot at night, and carrying a large side of mutton, was surrounded by a group of around 20 dingoes. The dingoes were ‘howling and snapping about him, but still keeping out of reach’ [81] (p. 10). His ‘screams of terror’ brought assistance from three men in the nearby homestead, who rode up with dogs and drove away the dingoes [81] (p. 10).	[81]
~1850	Unkn.	Para River (SA)	Adult	M/P	A mounted rider, Mr. William Grey, was ‘pursued by a large pack of wild native dogs, and had to fly for his life’ [81] (p. 10). See Figure A1 for a contemporary illustration of this event.	[81]
1850	March/April	Coonabarabran (NSW)	~5	Unkn.	An unnamed five-year-old child, daughter of shepherd Peter Standley, lost in the Bush at Marian Park Station, was found dead, with her frock and bonnet nearby, after a period of 10 days. The ‘mutilated bones’ were in a state consistent with the child having been ‘devoured by native dogs’ [282] (p. 2).	[282]
1850	January/February	Molong Creek, Bathurst district (Vic)	Adult	Unkn.	The body of sawyer Thomas Farrell, described as having had a serious drinking problem, was found in the Bush partly eaten by dingoes. It was assumed at the time that the animals had attacked and killed him while he was incapacitated by strong liquor.	[283]
1851	October	Mount Gambier (SA)	~3	Unkn.	According to a report in the *Argus*, entitled ‘Melancholy loss of a child’, the three-year-old son of Mr. Grant ‘strayed from his father’s house, and perished in the bush, he was nearly all devoured by the native dogs’ [284] (p. 2).	[284]
1851	December	Bathurst district (NSW)	~2	Unkn.	A toddler (name and sex not provided), apparently the child of a shepherd, left the family’s hut on a sheep station and disappeared in the Bush; ‘The heart-rending conclusion is that the infant has been devoured by the native dogs’ [285] (p. 3).	[285]
1864	August	Queanbeyan (ACT)	Adult	Unkn.	A shepherd, name unknown, went missing in the Bush without a trace: ‘It is presumed that the unfortunate man, who was given to drink, had in a fit of drunkenness either wandered and lost himself, or in his helpless and unconscious state been attacked and devoured by native dogs’ [286] (p. 2).	[286]
1867	January	Muswellbrook, Upper Hunter region (NSW)	4	Unkn.	The young child (unnamed, sex not given) of Charles Laurence, a shepherd on Ravensworth Station, Sandy Creek, disappeared in the Bush, and ‘it is feared it has been devoured by native dogs’ [287] (p. 24).	[287]
1870	Unkn.	Oyster Harbour (WA)	Adult	Unkn.	According to a brief newspaper report, the skeletal remains of John Doggett were found in the scrub: ‘It is supposed that the native dogs dragged him up in the bush and devoured him’ [288] (p. 2).	[288]
Pre-1872	Unkn.	‘Medindi’ (probably Menindee) district, Darling River (NSW)	Adult	Unkn.	A drover on Pammamera cattle station related a story about an unnamed woman who was reportedly killed and eaten by a dingo (or dingoes) on the property. One of the animals was supposedly found ‘in a choking condition, his mouth and throat full of the luxuriant tresses of their late victim’ [289] (pp. 16–17). No further information has been forthcoming about this account, which, prior to this written record, possibly only existed in the oral history of the station workers and wider rural community.	[289]
1875	13 March	Murrumburrah (NSW)	Adult	3	A cattleman (a Mr. Barnes) was engaged in looking after his stock when ‘he was suddenly attacked by three half-bred dingoes, which tore his trousers, and inflicted a severe wound on one of his legs. He managed to beat them off, and mounting his horse rode away from the savage mongrels, glad to escape without further treatment from their fangs’ [290] (p. 5). Given the early context these canids are unlikely to have been ‘half-bred’ domestic dog/dingo crosses but rather pure dingoes or hybrids with dominant dingo ancestry (see, e.g., [261]).	[290]
1876	17 September	Thompson’s Swamp (NSW)	Children and adults	M/P	Two lads were attacked at night in the Bush by dingoes. They climbed a tree to safety. Inspector Lloyd and police trooper Amies, and various patrons from a nearby pub, came to their assistance and were themselves attacked. According to one, ‘I felt myself bitten in the hand, and turning instantly I saw the brute six feet from me, and before I had time to defend myself in any way, he sprang at me a second time, biting me on the thigh’ [229] (p. 4). The policemen fired on the dingoes, killing one and wounding another. The other dingoes retreated.	[229]
1876	4 May	Maryborough district (Qld)	Adult	1	A settler woman was aggressively confronted by a dingo that had been raiding her farm for poultry.	[291]
1879	April	Fassifern Scrub [present-day Kalbar], (Qld)	5	Unkn.	The young child (Herman, or Hermann, Gerchow) of a German settler was lost in the scrub in the Fassifern area on 15 April 1879. A search was conducted over six days, but he was never found; ‘The general opinion in the neighbourhood is that the child has been devoured by native dogs, which abound in the scrub’ [292] (p. 2).	[292]
1880	14 December	Munna Creek, Miva (Qld)	2	Unkn.	A two-year-old boy, Willie Gesch, disappeared from the verandah of the family’s home on a Munna Creek selection, after being left unsupervised by his mother for about 10 min. No traces of the child were found. According to contemporary newspaper reportage, his parents believed that dingoes seen prowling around the Gesch selection on the day of his disappearance had seized Willie from the verandah and dragged him away into the Bush and eaten him. See Appendix C for a detailed discussion.	[174,175]
~1881	Unkn.	Mount Drummond (SA)	Adult	~20	A writer claimed that while camping alone in remote bushland he or she was surrounded at night by a pack of up to 20 dingoes. They made a large fire and kept it going all night to keep them away. The dingoes made repeated rushes at him/her, causing he/her to jump up shouting and waving firebrands at them.	[208]
1883	April/May	Kanyaka district, Mount Gambier (SA)	2.5	Unkn.	James ‘Jimmy’ Bole, the young son (aged 2.5 years) of a farmer, went missing in the Bush. A search was conducted by Aboriginal trackers and over 50 horsemen. After days of fruitless searching the police concluded that ‘he must have been devoured by the wild dogs, which are very numerous in that part of the country, which is about the roughest in the district, and the small size of the boy … would present no obstacle to his being dragged into one of the holes where the dogs kennel and all trace of him be lost’ [293] (p. 5). Decades later it was reported that long after the search had been abandoned his remains were found in a mountain gully, alongside the cricket bat he had been carrying when he went missing [294].	[293,294]
1884	Unkn.	Arangba, Gayndah district (Qld)	Adult	Unkn.	The partly eaten remains of a Chinese man were found in the Bush, purportedly next to some opium and an opium pipe. The latter items, if they existed, clearly satisfied the prejudiced view of Chinese settlers as depraved opium addicts [107], leading to the conclusion that dingoes had attacked the man while he was under the effects of the drug.	[295]
1884	Unkn.	Dawson River (Qld)	Adult	2	It was claimed, in a ‘Fifty years ago’ section of the *Maryborough Chronicle*, that in 1884 a young cattlewoman tried to rescue some cattle from a dingo attack and was set upon by the animals, climbing a tree to escape [296] (p. 2). See Figure A1.	[296]
1885	July	Bundaberg area (Qld)	3	Unkn.	A young child was lost in the Bush without a trace; ‘The police have gone in search, but it is feared the dingoes have done their work with the child’ [35] (p. 3).	[35]
1887	September	Carew, Tatiara region (SA)	3.5	Unkn.	A toddler, Thomas Carson, wandered away from his home on 2 September 1887 and vanished in the Bush. Contemporary newspaper reports did not mention the possibility that dingoes were involved in his death [297]. However, a letter written to the *Chronicle* on 24 March 1932, apparently from a family member, stated that ‘Being on the edge of the Ninety Mile Desert, which was infested with dingoes, it is the opinion of old bushmen that he was devoured by wild dogs’ [298] (p. 2).	[297,298]
1888	April	Saddler’s Creek (NSW)	Adult	M/P	The caretaker of a station at Saddler’s Creek, a man named Moran, ‘while walking through one of the station paddocks was ferociously attacked by a mob of native dogs’ [299] (p. 13).	[299]
1889	Possibly April	Carpendeit, Heytesbury forest (Vic)	Adult	M/P	A man was skinning a bullock in a paddock when a pack of dingoes approached him in a threatening manner. His sheepdog took on the dingoes and was quickly killed. The man fled.	[300]
~1889	Unkn.	Queensland	Adult	6	A swaggie (itinerant Bush labourer) who made a solitary journey on foot from Melbourne to the far north of Queensland recalled being attacked by dingoes while camped alone in the Bush: ‘One night when I was in the act of preparing a [johnny-]cake, after lighting a large fire for ashes, six dingoes came and took possession of the camp. I was obliged to retire, and leave them to devour all my flour and a piece of salt meat I had in my tucker bag. They showed their teeth freely, and growled rather more than was pleasant. I took a long stick of ironbark, set fire to one end of it, and warded them off by thrusting it against their noses. They followed me over three miles, when they gradually fell behind’ [209] (p. 26).	[209]
1891	July	Budgerum, Boort district (Vic)	~2–3	2	In the 10 July edition of *The Boort Standard* it was reported that three days before the toddler-aged child of a selector couple momentarily strayed outside the family’s house on a remote selection and was seized and carried off into the scrub by two dingoes [193]. The child’s body was never recovered. In the 31 July edition of *The Boort Standard*, however, a correspondent claiming to be from the Budgerum district stated, in a perfunctory letter, that ‘such an occurrence has not happened in the district’ [194] (p. 1). No further information about this affair has been uncovered.	[193,194]
1899	May	Upper Murray (NSW)	Adult	M/P	A woman who was lost in the Bush at one stage encountered a ‘mob’ of dingoes ‘and had to take refuge in a tree’ [301] (p. 2). The dingoes crowded around the tree for several hours, snarling and snapping at her. Eventually the dingoes ran off in pursuit of some wallabies, providing an opportunity for the woman to escape.	[301]
1901	July	Maryborough district (Qld)	~10	1	A young farm boy, George Emery, had been followed by a dingo for three evenings in succession when out milking cows, and ‘On the third evening the dingo made a violent attack, and the little fellow had a very narrow escape, but as it was a very rocky creek, the boy managed to get over to the other side of the creek, and as stones were plentiful he kept the savage animal at bay’ [302] (p. 3). A police search for the dingo responsible for the attack uncovered a nearby denning site with pups and adult dingoes present.	[302]
1902	Late April/early May	Gammon Ranges (SA)	Adult	M/P	An article in *The Port Augusta Dispatch* reported that an elderly male prospector named Harry Hemming, who was ‘somewhat feeble and near sighted’, was missing for several weeks in a remote part of the Gammon Ranges, where ‘The surrounding country is very mountainous and ragged and abounding in native dogs’ [303] (p. 3). Aboriginal trackers eventually led a police search party to Hemming’s body in a valley below a high ridge on which the prospector had been camping. Hemming had been ‘almost totally devoured’ by dingoes [303] (p. 3). The old man had supposedly fallen from an adjacent precipice into the valley below. The article claimed that Hemming’s thigh was broken during the fall, and he sustained other incapacitating injuries. He had survived for long enough, however, to remove his belt and singlet and place them on a rock. The newspaper article also stated that: ‘The trackers express the opinion that the (dingoes) attacked him on top of the hill and in his endeavour to get down the cliff for protection, lost his footing and fell’ [303] (p. 3). The police report on the search for Harry Hemming is held in the State Records of South Australia [304]. Dated 31 May 1902 and written by the constable in charge of the search, R.G. (Robert Gibson) Birt from Beltana Police Station, it confirms the accuracy of some of the information in the newspaper article [304]. Birt’s report comprises a four page hand-written document, almost all of which was devoted to explaining the history and complex logistics of the search for Hemming (including an initial foray that was abandoned owing to the unexpectedly difficult terrain) and the hardships the party faced in the rough Bush country, presumably to justify to his superior the time and expense devoted to the long expedition and to emphasise the remarkable achievement it yielded (an outcome Birt attributed to the superb skills of the Aboriginal trackers, ‘Claypan George’, ‘Bennie’, ‘Bobbie’ and ‘Walter’, which he unstintingly praised). Scant detail (~9 of 132 lines, with 5–7 words per line) was devoted to a description of Hemming’s body and its context, the ‘crime scene’, and supposition as to cause of death [304]. Birt noted simply that it appeared to him the prospector had accidentally fallen to his death at some stage between the 16th and 18th of April, and that dingoes had eaten all of the flesh from his body. (The search party buried Hemming’s body on the spot and placed rocks over the simple Bush grave to protect it from scavenging dingoes [304]). The report does not contain any mention of the theory attributed to the trackers in *The Port Augusta Dispatch* [303] that dingoes had attacked Hemming at his camp, so either this part of the newspaper story was an embellishment or the trackers’ theory was omitted from Birt’s report. The Australian author Ion ‘Jack’ Idriess wrote (possibly with Birt’s involvement) an account of the search for Harry Hemming in his 1946 book *Man Tracks* [305] (pp. 86–114), but the dingo attack theory was not mentioned.	[303,304]
1906	March	Murwillumbah area (NSW)	2	Unkn.	A two-year-old child, surname Hitchens, was lost in the Bush: ‘The child has not been heard of since, and it is thought has made its way into the river and been drowned, or been taken by dingoes’ [306] (p. 2).	[306]
Pre-1907	Unkn.	Granville/Parramatta district (NSW)	Adult	M/P	Referring to ‘The red road which joins the Southern and Western roads at Granville (known as the Dog-trap Road)’, a Sydney resident wrote: ‘It is not so many years since a pack of dingoes attacked a butcher’s cart loaded with sides of beef, on this road; the driver and horse only escaped by the skin of their teeth’ [66] (p. 10).	[66]
1909	~September	Clifton district, Illawarra (NSW)	Adult	M/P	A Clifton miner, John Pallier, searching for wild Bush flowers, was lost in the thick scrub on the Illawarra escarpment. According to the *Singleton Argus*: ‘A pack of dingoes attacked Pallier, forcing him to seek refuge in the branches of a tree, where he sat the whole night’ [307] (p. 6). Eventually the dingoes appeared to disperse. The following day, after Pallier had fallen out of the tree, he kept wandering, but the dingoes continued to stalk him for two days and nights, ‘watching his movements from the shelter of the scrub’ [307] (p. 6). The miner was found alive near Coledale.	[307]
~1910	Unkn.	K’gari (Qld)	Adult	M/P	According to the oral history of Gordon Peters, who was a bullock-driver on K’gari in the early 1900s, fellow K’gari resident Christy Mathison was hunting in the scrub on the island one day when he encountered a group of dingoes comprising two adults, a subadult, and four young pups. Two other adult dingoes were nearby. According to Peters the dingoes became ‘very agitated and then aggressive’ [162] (p. 163). Sensing an attack was imminent, Mathison ran away and hid in the root structure of a fig tree. Peters claimed that: ‘The dingoes came on, starting to attack, so Christy aimed his double-barrel shotgun out between the roots and managed to shoot five of the nine dingoes. The other four escaped into the bush’ [162] (p. 163).	[162]
1912	~18 August	Booyal, Bundaberg district (Qld)	4	Unkn.	A four-year-old boy, Harold Halliday, lost in the scrub at Booyal on 18 August, ‘has been killed and eaten by the wild dogs which infest the locality’, according to police findings [308] (p. 4). *The Brisbane Courier* reported that: ‘Batches of young pups have been seen frequently by the search parties, many of whom share the opinion of the police’ [308] (p. 4). The search, led by an Aboriginal tracker (identified in the newspaper report only as Tommy), lasted for about a fortnight. The child’s body was never found.	[308]
1916	~January	Daymar (Qld)	3	Unkn.	The remains of a three-year-old boy, Edgar Tighe, who had been lost in the Bush, were found several kilometres from his home: ‘One leg was bitten off either by foxes or dingoes. He had got over a six feet high dog proof netting fence, which surrounded the paddock where the home is situated’ [309] (p. 3).	[309]
1917	September	Pikedale (Qld)	9 or 10	Unkn.	A young boy, Thomas Kenny, disappeared while shepherding a flock of sheep on his own in the Bush near Pikedale. An Aboriginal tracker identified the boy’s tracks near his father’s hut, from where he had initially set out, but no further traces of him were found. It was assumed at the time he was ‘eaten by native dogs’ [310] (p. 7).	[310]
1924	Unkn.	K’gari (Qld)	Adult	M/P	The lighthouse-keeper at Inskip Point was reportedly ‘chased from the beach where he had been fishing by a number of fierce (wild dingoes)’ [167] (p. 6).	[167]
Pre-1924	Unkn.	Braidwood district (NSW)	Adult	1	According to one source [231], an unnamed elderly Chinese man was walking alone at night when he noticed an ‘old man’ dingo following him. Frightened, he broke into a run and was immediately set upon and attacked by the animal. The man yelled in fright and some people came to his aid, scaring away the dingo. A correspondent to the *Bulletin* provided an earlier version of this story in 1924 [311]. This writer claimed that the man had noticed the presence of several ‘warrigals’ (dingoes) around his hut in the days leading up to the incident [311] (p. 24). It was said that he was on his way to a creek when he was set upon by three dingoes. One dingo succeeded in seizing him by the throat before he beat them off with a bucket and barricaded himself in his hut [311].	[231,311]
1925	Unkn.	Kilcoy, Mollman’s Scrub (Qld)	10.5	M/P	A young boy, Neil Barnett, out looking for a horse on foot, was ‘confronted by a dingo, which made at [*sic*] attempt to attack him’ [312] (p. 4). A pack then surrounded him. He picked up a stick to defend himself, but then decided to climb a tree for safety. The dingoes then attacked a nearby calf, so the boy ‘jumped from the tree and belted the dingoes off’ [312] (p. 4).	[312]
1925	March	Hampden, Mackay district (Qld)	2.5	Unkn.	On 19 March, the *Maryborough Chronicle* reported that the remains of a toddler, John Henry O’Sullivan, aged 2.5 years, still had not been found after extensive searching. His parents were selectors who owned a small block at Hampden. His mother had been in the Bush alone with him and his younger sibling, mustering cattle, when Henry wandered off unnoticed. The police concluded he had been taken by dingoes or a wedge-tailed eagle.	[93]
Pre-1927	Unkn.	North coast district (NSW)	Child	1	An unnamed ‘farm lad’ was ‘set upon’ by a dingo, which ‘bolted as soon as he screamed with fear’ [231] (p. 18).	[231]
1927	Unkn.	Paterson (lower Hunter region of NSW)	Youth	5	A ‘youth’, R.S. Kidd, was riding in the Bush when he observed a pack of five dingoes attacking a cattle dog. He intervened in an attempt to save the dog, ‘shooing’ away the dingoes, but was attacked and sustained a serious injury to his leg. He managed to ‘beat them off with a stick’ [313] (p. 6). The dog was killed.	[313]
1927	12 December	Nambour district (Qld)	Child	4	A shepherd’s son (surname Lowe) went to look for some sheep that had got out of a fenced dingo-proof enclosure. He found one of the sheep dead in a gully, with four or five dingoes nearby. He tried to frighten the dingoes away by rushing at them, shouting and throwing a stone. One ran away, but the others ‘made a rush for the boy’, prompting him to scream loudly with fright [314] (p. 3). His brothers and a neighbour came to his aid, ‘and there found the dingoes attacking him. The brutes had torn his shirt to ribbons, but fortunately, had not hurt the flesh. It is considered that the lad had a narrow escape’ [314] (p. 3).	[314]
1928	27 May	Merewether (now a suburb of Newcastle) (NSW)	Adult	M/P	A man named George Cox was returning home on foot from a fishing excursion when he was surrounded and ‘attacked by a pack of hungry dingoes’ [315] (p. 4). He struck the nearest dingo with a stick, but the others closed in on him. Cox climbed the nearest tree and remained there until early morning; ‘All through the night the dingoes made wild leaps at his feet and then early in the morning ran off into the bush’ [315] (p. 4). A search party found him in the scrub at 3 a.m.; ‘They reached the tree where he had taken refuge when seven dingoes returned and attacked the defenceless men. There followed a furious fight in which Cox joined and eventually one of the beasts was killed, and the rest made off. Cox reached home in a state of collapse’ [315] (p. 4).	[315]

## References

[1] Corbett L.K. (1995). The Dingo in Australia and Asia.

[2] Balme J., O’Connor S., Fallon S. (2018). New dates on dingo bones from Madura Cave provide oldest firm evidence for arrival of the species in Australia. Sci. Rep..

[3] Smith B.P., Savolainen P., Smith B.P. (2015). The origin and ancestry of the dingo. The Dingo Debate: Origins, Behaviour and Conservation.

[4] Ballard J.W.O., Wilson L.A.B. (2019). The Australian dingo: Untamed or feral?. Front. Zool..

[5] Koungoulos L. (2021). Domestication through dingo eyes: An Australian perspective on human-canid interactions leading to the earliest dogs. Hum. Ecol..

[6] Bryson J. (1985). Evil Angels: The Case of Lindy Chamberlain.

[7] Craik J. (1987). The Azaria Chamberlain case and questions of infanticide. Aust. J. Cult. Stud..

[8] Marcus J. (1989). Prisoner of discourse: The dingo, the dog and the baby. Anthropol. Today.

[9] Chamberlain-Creighton L. (2004). Through My Eyes: The Autobiography of Lindy Chamberlain-Creighton.

[10] Bryson J., Staines D., Arrow M., Biber K. (2009). Against the tactician. The Chamberlain Case: Nation, Law, Memory.

[11] Craik J., Staines D., Arrow M., Biber K. (2009). The Azaria Chamberlain case: Blind spot or black hole in Australian cultural memory. The Chamberlain Case: Nation, Law, Memory.

[12] Chamberlain M. (2012). Heart of Stone: Justice for Azaria.

[13] Bryson J. (2013). The myths of Azaria, so many: Superstition ain’t the way. Griffith Rev..

[14] Middleweek B. (2017). Dingo media? The persistence of the “trial by media” frame in popular, media, and academic evaluations of the Azaria Chamberlain case. Fem. Media Stud..

[15] Middleweek B. (2021). Feral Media? The Chamberlain Case, 40 Years on.

[16] Kirkham A. (2020). Chamberlain: A retrospective. Vic. Bar News.

[17] Appleby R., Smith B.P. (2015). Dingo-human conflict: Attacks on humans. The Dingo Debate: Origins, Behaviour and Conservation.

[18] Appleby R., Mackie J., Smith B., Bernede L., Jones D. (2018). Human-dingo interactions on Fraser Island: An analysis of serious incident reports. Aust. Mammal..

[19] West J.G. (2011). Changing patterns of shark attacks in Australian waters. Mar. Freshw. Res..

[20] Smith B.P., Litchfield C.A. (2009). A review of the relationship between indigenous Australians, dingoes (*Canis dingo*) and domestic dogs (*Canis familiaris*). Anthrozoös.

[21] Brumm A. (2021). Dingoes and domestication. Archaeol. Ocean..

[22] Tench W. (1789). A Narrative of the Expedition to Botany Bay with an Account of New South Wales, Its Productions, Inhabitants, &c. to Which Is Subjoined, a List of the Civil and Military Establishments at Port Jackson.

[23] (1942). Fight with Dingo. The Macleay Chronicle.

[24] Beier P. (1991). Cougar attacks on humans in the United States and Canada. Wildl. Soc. Bull..

[25] Langley R.L. (2005). Alligator attacks on humans in the United States. Wilderness Environ. Med..

[26] White L.A., Gehrt S.D. (2009). Coyote attacks on humans in the United States and Canada. Hum. Dimens. Wildl..

[27] Hong S., Do Y., Kim J.Y., Cowan P., Joo G.-J. (2017). Conservation activities for the Eurasian otter (*Lutra lutra*) in South Korea traced from newspapers during 1962–2010. Biol. Conserv..

[28] Isaacs V., Kirkpatrick R. (2003). Two Hundred Years of Sydney Newspapers: A Short History.

[29] Mayer H. (1968). The Press in Australia.

[30] Walker R.B. (1976). The Newspaper Press in New South Wales, 1803–1920.

[31] Ingleton G.C. (1965). True Patriots All, or News from Early Australia—As Told in a Collection of Broadsides, Garnered & Decorated by Geoffrey Chapman Ingleton.

[32] Flanders O. (2019). When Dingoes ‘Attack’: A Look at Human-Dingo Interactions as Reported by the Media. Unpublished. Bachelor of Psychological Science (Honours) Thesis.

[33] Davis T. ‘Recollections of Thomas Davis’ Collected by Steele Rudd.

[34] de Looper M.W. (2014). Death Registration and Mortality Trends in Australia 1856–1906. Unpublished Ph.D. Thesis.

[35] (1885). Bundaberg. Maryborough Chronicle, Wide Bay and Burnett Advertiser.

[36] Koungoulos L., Fillios M., Bethke B., Burtt A. (2020). Between ethnography and prehistory: The case of the Australian dingo. Dogs: Archaeology Beyond Domestication.

[37] Allen B.L., Higginbottom K., Bracks J.H., Davies N., Baxter G.S. (2015). Balancing dingo conservation with human safety on Fraser Island: The numerical and demographic effects of humane destruction of dingoes. Australas. J. Environ. Manag..

[38] Conroy G.C., Lamont R.W., Bridges L., Stephens D., Wardell, Johnson A., Ogbourne S.M. (2021). Conservation concerns associated with low genetic diversity for K’gari–Fraser Island dingoes. Sci. Rep..

[39] Stephens D., Wilton A.N.P., Fleming J.S., Berry O. (2015). Death by sex in an Australian icon: A continent-wide survey reveals extensive hybridization between dingoes and domestic dogs. Mol. Ecol..

[40] Rupprecht C.E., Freuling C.M., Mani R.S., Palacios C., Sabeta C.T., Ward M., Fooks A.R., Jackson A.C. (2020). A history of rabies—The foundation for global canine rabies elimination. Rabies.

[41] Linnell J.D.C., Andersen R., Andersone Z., Balciauskas L., Blanco J.C., Boitani L., Brainerd S., Beitenmoser U., Kojola I., Liberg O. (2002). The fear of wolves: A review of wolfs attacks on humans. NINA Oppdragsmeld..

[42] Norton C. (2012). Tourist Tells of Dingo Attack. Sunshine Coast Daily.

[43] Lennox R. (2021). Dingo Bold: The Life and Death of K’gari Dingoes.

[44] Stephens K. (2014). Fraser Island Dingoes Not to Blame Says Paramedic. Brisbane Times.

[45] (1997). Dingoes Are Dangerous. MOONBI, 91.

[46] Royal Commission of Inquiry into Chamberlain Convictions (1987). Report of the Commissioner the Hon. Mr. Justice T.R. Morling.

[47] Catling P.C. (1979). Seasonal variation in plasma testosterone and the testis in captive male dingoes, *Canis familiaris dingo*. Aust. J. Zool..

[48] Breckwoldt R. (1988). A Very Elegant Animal the Dingo.

[49] Thomson P.C. (1992). The behavioural ecology of dingoes in north-western Australia: II. Activity patterns, breeding season and pup rearing. Wildl. Res..

[50] Purcell B. (2010). Dingo.

[51] Smith B.P., Smith B.P. (2015). Biology and behaviour of the dingo. The Dingo Debate: Origins, Behaviour and Conservation.

[52] Cursino M., Harriott S.L., Allen B.L., Gentle M., Leung L.K.-P. (2017). Do female dingo–dog hybrids breed like dingoes or dogs?. Aust. J. Zool..

[53] O’Neill A.J., Cairns K.M., Kaplan G., Healy E. (2017). Managing dingoes on Fraser Island: Culling, conflict, and an alternative. Pac. Conserv. Biol..

[54] Allen B. (2010). Skin and bone: Observations of dingo scavenging during a chronic food shortage. Aust. Mammal..

[55] Stevenson J.B. (1880). Seven Years in the Australian Bush.

[56] Tenterfield (1914). The Dingo. Sydney Mail.

[57] (1997). Dingo Pack Attacks Good Samaritan. The Advertiser.

[58] Dillon M. (2013). Car Crash Survivor Attacked by Dingo. NT News.

[59] Robson v Territory Insurance Office (2013). Northern Territory Supreme Court (NTSC) 27.

[60] (1804). Postscript. The Sydney Gazette and New South Wales Advertiser.

[61] (1805). Culture of Hops in Great Britain. The Sydney Gazette and New South Wales Advertiser.

[62] (1804). Sydney. The Sydney Gazette and New South Wales Advertiser.

[63] (1808). Sydney. The Sydney Gazette and New South Wales Advertiser.

[64] (1831). Native Dogs. The Sydney Gazette and New South Wales Advertiser.

[65] (1939). Dingoes Near Sydney. The Sydney Morning Herald.

[66] Kaleski R. (1907). The Noble Dingo: Further Respectfully Considered in His Downsittings, His Uprisings, and His Family Relations. The Bookfellow.

[67] (1805). Sydney. The Sydney Gazette and New South Wales Advertiser.

[68] Phillip A. (1789). The Voyage of Governor Phillip to Botany Bay with an Account of the Establishment of the Colonies of Port Jackson and Norfolk Island, Compiled from Authentic Papers, Which Have Been Obtained from the Several Departments to Which Are Added the Journals of Lieuts. Shortland, Watts, Ball and Capt. Marshall with an Account of Their New Discoveries.

[69] McNay M.E. (2002). Wolf-human interactions in Alaska and Canada: A review of the case history. Wildl. Soc. Bull..

[70] McNay M.E., Mooney P.W. (2005). Attempted predation of a child by a gray wolf, *Canis lupus*, near Icy Bay, Alaska. Ott. Nat..

[71] Newsome T.M., Stephens D., Ballard G.-A., Dickman C.R., Fleming P.J.S. (2013). Genetic profile of dingoes (*Canis lupus dingo*) and free-roaming domestic dogs (*C. l. familiaris*) in the Tanami Desert, Australia. Wildl. Res..

[72] Newsome T.M., Ballard G.-A., Crowther M.S., Fleming P.J.S., Dickman C.R. (2014). Dietary niche overlap of free-roaming dingoes and domestic dogs: The role of human-provided food. J. Mammal..

[73] Smith B.P., Vague A.-L. (2007). The denning behaviour of dingoes (*Canis dingo*) living in a human-modified environment. Aust. Mammal..

[74] Warriner J. (2018). Hefty Fine for Newcrest after Wild Dingoes Maul Woman at Pilbara Mine. ABC News.

[75] Sidney S. (1853). The Three Colonies of Australia: New South Wales, Victoria, South Australia; Their Pastures, Copper Mines, & Gold Fields.

[76] Philanthus (1804). To the Printer of the Sydney Gazette. The Sydney Gazette and New South Wales Advertiser.

[77] Parker M.A. (2006). Bringing the Dingo Home: Discursive Representations of the Dingo by Aboriginal, Colonial and Contemporary Australians. Unpublished Ph.D. thesis.

[78] Parker M. (2007). The cunning dingo. Soc. Anim..

[79] (1814). Sydney. The Sydney Gazette and New South Wales Advertiser.

[80] Appleby R.G., Smith B.P., Carr N., Young J. (2018). Do wild canids kill for fun. Wild Animals and Leisure: Rights and Wellbeing.

[81] (1866). The Dingo. The Australian News for Home Readers.

[82] (1929). Death and Dingoes. Western Star and Roma Advertiser.

[83] Macintosh N.W.G. (1956). Trail of the dingo. The Etruscan: Staff Magazine of the Bank of New South Wales.

[84] Wright S. (1968). The Way of the Dingo.

[85] Cree B. (1969). The Bountyhunters. Mimag (Mount Isa Mines Limited).

[86] Rolls E.C. (1969). They All Ran Wild: The Story of Pests on the Land in Australia.

[87] Macintosh N.W.G., Fox M.W. (1975). The origin of the dingo: An enigma. The Wild Canids: Their Systematics, Behavioral Ecology and Evolution.

[88] Evans I.J. (2010). Touching Magic: Deliberately Concealed Objects in Old Australian Houses and Buildings. Unpublished Ph.D. thesis.

[89] Blee J., Waldron D. (2016). Banshees. Goldfields and the Gothic: A Hidden Heritage and Folklore.

[90] Morgan J. (1852). The Life and Adventures of William Buckley, Thirty-Two Years a Wanderer amongst the Aborigines of the then Unexplored Country Round Port Phillip, Now the Province of Victoria.

[91] Donovan P.M., Waldron D. (2016). Buckley’s bunyip. Goldfields and the Gothic: A Hidden Heritage and Folklore.

[92] (1907). The Missing Child. Norseman Times.

[93] (1925). Lost in the Bush. Bororen Child Still Missing. Maryborough Chronicle, Wide Bay and Burnett Advertiser.

[94] Rogers J. (2012). Baby-Snatching Eagle Hoaxers Tapped into an Ancient Myth. The Globe and Mail.

[95] Rogers J. (2015). Eagle.

[96] Dawson J. (1881). Australian Aborigines: The Languages and Customs of Several Tribes of Aborigines in the Western District of Victoria, Australia.

[97] Murray R. (2020). The Confident Years: Australia in The 1920s.

[98] Wood Jones F. (1921). The status of the dingo. Trans. R. Soc. S. Aust..

[99] Barker B.C.W., Macintosh A. (1979). The dingo—A review. Arch. Phys. Anth. Ocean..

[100] Corbett L.K., Newsome A.E., Fox M.W. (1975). Dingo society and its maintenance: A preliminary analysis. The Wild Canids: Their Systematics, Behavioral Ecology and Evolution.

[101] Fleming P., Corbett L., Harden R., Thomson P. (2001). Managing the Impacts of Dingoes and Other Wild Dogs.

[102] Newsome T.M. (2014). Makings of icons: Alan Newsome, the red kangaroo and the dingo. Hist. Rec. Aust. Sci..

[103] Coleman J.T. (2004). Vicious: Wolves and Men in America.

[104] Linnell J.D.C., Alleau J., Angelici F.M. (2016). Predators that kill humans: Myth, reality, context and the politics of wolf attacks on people. Problematic Wildlife: A Cross-Disciplinary Approach.

[105] Gandevia B., Gandevia S. (1975). Childhood mortality and its social background in the first settlement at Sydney Cove, 1788–1792. Aust. Paediat. J..

[106] Shumack S. (1967). An Autobiography or Tales and Legends of Canberra Pioneers.

[107] Waterhouse R. (2005). The Vision Splendid: A Social and Cultural History of Rural Australia.

[108] McCalman J., Kippen R., Bashford A., Macintyre S. (2013). Population and health. The Cambridge History of Australia. Volume 1: Indigenous and Colonial Australia.

[109] Fullerton M.E. (1928). The Australian Bush.

[110] Fullerton M.E. (1931). Bark House Days.

[111] Torney K. (2005). Babes in the Bush.

[112] Pickard J. (2007). The transition from shepherding to fencing in colonial Australia. Rural Hist..

[113] Gerrard J. (2017). The interconnected histories of labour and homelessness. Labour Hist..

[114] Davidson G., Brodie M., Davidson G., Brodie M. (2005). Introduction. Struggle Country: The Rural Ideal in Twentieth Century Australia.

[115] Brodie M., Davidson G., Brodie M. (2005). The politics of rural nostalgia between the wars. Struggle Country: The Rural Ideal in Twentieth Century Australia.

[116] Reynolds P., Staines D., Arrow M., Biber K. (2009). The Azaria Chamberlain case: Reflections on Australian identity. The Chamberlain Case: Nation, Law, Memory.

[117] Letnic M., Koch F. (2010). Are dingoes a trophic regulator in arid Australia? A comparison of mammal communities on either side of the dingo fence. Austral Ecol..

[118] Yelland L. (2012). Holding the Line: A History of the South Australian Dog Fence Board, 1947 to 2012.

[119] Glen A.S., Short J. (2000). The control of dingoes in New South Wales in the period 1883–1930 and its likely impact on their distribution and abundance. Aust. Zool..

[120] Allen B.L., West P. (2013). The influence of dingoes on sheep distribution in Australia. Aust. Vet. J..

[121] Waterhouse R. (2000). Australian legends: Representations of the bush, 1813–1913. Aust. Hist. Stud..

[122] Waterhouse R. (2002). Rural culture and Australian history: Myths and realities. Arts.

[123] Allen B.L., West P. (2015). Dingoes are a major causal factor for the decline and distribution of sheep in Australia. Aust. Vet. J..

[124] Levy S. (2009). The dingo dilemma. BioScience.

[125] Philip J. (2016). Representing the Dingo: An Examination of Dingo–Human Encounters in Australian Cultural and Environmental Heritage. Unpublished. Ph.D. Thesis.

[126] (1925). The Deadly Dingo. Morning Bulletin.

[127] Newsome T.M., Ballard G.-A., Dickman C.R., Fleming P.J.S., van de Ven R. (2013). Home range, activity and sociality of a top predator, the dingo: A test of the Resource Dispersion Hypothesis. Ecography.

[128] (1933). Not Man-Killer: Australian Dingo. The Courier-Mail.

[129] Binks B., Kancans R., Stenekes N. (2015). Wild Dog Management 2010 to 2014. National Landholder Survey Results.

[130] Young S.P. (1944). The Wolves of North America. Part I: Their History, Life Habits, Economic Status, and Control.

[131] Fritts S.H., Stephenson R.O., Hayes R.D., Boitani L., Mech L.D., Boitani L. (2003). Wolves and humans. Wolves: Behavior, Ecology, and Conservation.

[132] Jenness S.E. (1985). Arctic wolf attacks scientist—A unique Canadian incident. Arctic.

[133] Mech L.D. (1998). “Who’s afraid of the big bad wolf?”: Revisited. Int. Wolf.

[134] Jones K. (2011). Writing the wolf: Canine tales and North American environmental-literary tradition. Environ. Hist..

[135] Butler L., Dale B., Beckmen K., Farley S. (2011). Findings Related to the March 2010 Fatal Wolf Attack Near Chignik Lake, Alaska.

[136] Australian Bureau of Statistics 3105.0.65.001 Australian Historical Population Statistics, 2014: Table 2.1 Population, Age and sex, Australia, 30 June, 1901 Onwards. https://www.abs.gov.au.

[137] Sanders N., Staines D., Arrow M., Biber K. (2009). Azaria Chamberlain and popular culture. The Chamberlain Case: Nation, Law, Memory.

[138] Seal G., Staines D., Arrow M., Biber K. (2009). Dread, delusion and globalisation: From Azaria to Schapelle. The Chamberlain Case: Nation, Law, Memory.

[139] Allen B., Timm R.M., O’Brien J.M. (2006). Urban dingoes (Canis lupus dingo and hybrids) and human hydatid disease (Echinococcus granulosus) in Queensland, Australia. Proceedings of the 22nd Vertebrate Pest Conference.

[140] Allen B.L., Goullet M., Allen L.R., Lisle A., Leung L.K.-P. (2013). Dingoes at the doorstep: Preliminary data on the ecology of dingoes in urban areas. Landsc. Urban Plan..

[141] Young N.H., Staines D., Arrow M., Biber K. (2009). Against the odds: The fight to free Lindy Chamberlain. The Chamberlain Case: Nation, Law, Memory.

[142] Biber K., Staines D., Arrow M., Biber K. (2009). Judicial extracts. The Chamberlain Case: Nation, Law, Memory.

[143] Pierce P. (1999). The Country of Lost Children: An Australian Anxiety.

[144] Ward E. (1987). Who Killed Azaria Chamberlain?. The Washington Post.

[145] Johnson D., Staines D., Arrow M., Biber K. (2009). From fairy to witch: Imagery and myth in the Azaria case. The Chamberlain Case: Nation, Law, Memory.

[146] (1984). Forensic Evidence Played No Part in Verdict—Juror: ‘Lindy Guilty on the Basis of Her Story’. The Courier Mail.

[147] Australian Bureau of Statistics 3105.0.65.001 Australian Historical Population Statistics, 2019: Table 3.3 Population, Urban and Rural Areas, States and Territories, 30 June, 1911 Onwards. https://www.abs.gov.au.

[148] Franklin A. (2012). Dingoes in the Dock. New Scientist.

[149] Wood B., Staines D., Arrow M., Biber K. (2009). The trials of motherhood. The Chamberlain Case: Nation, Law, Memory.

[150] Walters B. (1995). The Company of Dingoes: Two Decades with Our Native Dog.

[151] Chew T., Willet C.E., Haase B., Wade C.M. (2019). Genomic characterization of external morphology traits in Kelpies does not support common ancestry with the Australian dingo. Genes.

[152] Howe A., Staines D., Arrow M., Biber K. (2009). Writing to Lindy—‘May I call you Lindy?’. The Chamberlain Case: Nation, Law, Memory.

[153] Fife-Yeomans J., Toohey P. (2010). Azaria Chamberlain Jury Secrets. Daily Telegraph.

[154] Peace A. (2001). Dingo discourse: Constructions of nature and contradictions of capital in an Australian eco-tourist location. Anthropol. Forum.

[155] Flannery T. (2012). Sorry, Lindy, We Should Have Known Better. The Sydney Morning Herald.

[156] (1997). Family Saves Boy from Attack by Dingoes. The Advertiser.

[157] Barlass T. (1998). Father Tells: The Night a Dingo Took Our Baby. The Advertiser.

[158] Stone L. (2019). ‘A Dingo Had Him by the Back of the Neck’: Family Recalls Fraser Island Attack. The Brisbane Times.

[159] Goetze E., Khan N. (2021). Toddler Bitten by Dingo on Queensland’s Fraser Island, Airlifted to Bundaberg Hospital. ABC News.

[160] Khan N. (2021). Dingo Bites 4yo Boy on Thigh on Fraser Island, in Second Attack in Weeks. ABC News.

[161] Marie J. (2021). Dingoes on Fraser Island-K’gari Losing Their Natural Fear of Humans, Says Local Mayor. ABC News.

[162] Williams F. (2002). A History of Fraser Island: Princess K’gari’s Fraser Island.

[163] Petrie R. (1995). Early Days on Fraser Island: 1913–1922.

[164] Sinclair J. (2015). Dingos of Fraser Island. Fraser Island Defenders Organisation. https://fido.org.au/dingos-of-fraser-island/.

[165] Beckmann E., Savage G. (2003). Evaluation of Dingo Education Strategy and Programs for Fraser Island.

[166] Curtis J. (1838). Shipwreck of the Stirling Castle, Containing a Faithful Narrative of the Dreadful Sufferings of the Crew, and the Cruel Murder of Captain Fraser by the Savages. Also, the Horrible Barbarity of the Cannibals Inflicted upon the Captain’s Widow, Whose Unparalleled Sufferings are Stated by Herself, and Corroborated by the Other Survivors. To Which Is Added, the Narrative of the Wreck of the Charles Eaton, in the Same Latitude.

[167] (1924). The Dingo Pest. Morning Bulletin.

[168] (1924). Fierce Dingoes. Maryborough Chronicle, Wide Bay and Burnett Advertiser.

[169] (1924). Starving Brutes on Fraser Island. The Farmer and Settler.

[170] Wilson J. (1935). Trip to Fraser Island. The Telegraph.

[171] White B.H. (1933). Hiking on Fraser. Variety of Experiences. Nature’s Wonderland. Maryborough Chronicle, Wide Bay and Burnett Advertiser.

[172] Roberts N.L. (1942). Fraser Island (Queensland). Walkabout.

[173] (1950). Fraser Is. Holiday. Maryborough Chronicle.

[174] (1880). Local and General. Maryborough Chronicle, Wide Bay and Burnett Advertiser.

[175] (1880). Local and General News. Gympie Times and Mary River Mining Gazette.

[176] (1932). Miva. Maryborough Chronicle, Wide Bay and Burnett Advertiser.

[177] (1938). Deaths. Maryborough Chronicle, Wide Bay and Burnett Advertiser.

[178] Carlson E.M. (1959). A Century of Settlement in the Miva District.

[179] Gesch G.E. (1954). To the Editor: Gootchie History. Maryborough Chronicle.

[180] (1981). ‘A Dingo Did Not Take My Brother’. The Gympie Times.

[181] Miles A. Gesch Family of Miva.

[182] Smith B.P., Smith B.P. (2015). The personality, behaviour and suitability of dingoes as companion animals. The Dingo Debate: Origins, Behaviour and Conservation.

[183] Smith B.P., Smith B.P. (2015). Dingo intelligence: A dingo’s brain is sharper than its teeth. The Dingo Debate: Origins, Behaviour and Conservation.

[184] Prentis M.D. (1991). The life and death of Johnny Campbell. Aborig. Hist..

[185] (1938). The Spirit of the Pioneers. Achievements of Mr. W. Gesch, Miva. Maryborough Chronicle, Wide Bay and Burnett Advertiser.

[186] Torney K. (2004). A city child lost in the bush. La Trobe Journal.

[187] (1907). White Child among Blacks. Northern Star.

[188] Meston A. (1898). Mundi and Coyeen. The Bulletin.

[189] (1898). Kidnapped by the Blacks. The Richmond River Herald and Northern Districts Advertiser.

[190] Behrendorff L., Belonje G., Allen B.L. (2018). Intraspecific killing behaviour of canids: How dingoes kill dingoes. Ethol. Ecol. Evol..

[191] (1804). Sydney. The Sydney Gazette and New South Wales Advertiser.

[192] (1804). Sydney. The Sydney Gazette and New South Wales Advertiser.

[193] (1891). Eaten Alive by Dingoes. The Boort Standard.

[194] Cummins P. (1891). A Disclaimer. The Boort Standard.

[195] (1878). No title. Mackay Mercury and South Kennedy Advertiser.

[196] (1878). Northern News. The Brisbane Courier.

[197] Butterworth L. (2019). Investigating death in Moreton Bay: Coronial inquests and magisterial inquiries. Qld. Rev..

[198] Taylor R., Lewis M., Powles J. (1998). The Australian mortality decline: All-cause mortality 1788–1990. Aust. N. Z. J. Public Health.

[199] (1804). Fatal Accident. The Sydney Gazette and New South Wales Advertiser.

[200] (1860). Local and Domestic. The Darling Downs Gazette and General Advertiser.

[201] (1865). The Lachlan. The Sydney Morning Herald.

[202] (1865). Singular Providence. The Sydney Morning Herald.

[203] (1870). Heroic Conduct of a Woman with Two Children Lost in the Bush. The Maitland Mercury and Hunter River General Advertiser.

[204] Rickard J. (2017). Australia: A Cultural History.

[205] (1875). Lost, and Supposed Death in the Bush. The Sydney Morning Herald.

[206] (1928). 102 Years of Age. Western Pioneer’s Death. Treed by Dingoes. Glen Innes Examiner.

[207] McGuire J. (1911). A Wild Colonial Boy: Thrilling Incidents of Early Days. The Adventures of John McGuire. Truth.

[208] H.J.C. (1881). Wild Dog Adventures. Evening Journal.

[209] (1889). A Terrible Tramp. The Albury Banner and Wodonga Express.

[210] (1884). Tree’d by Dingoes. The Bulletin.

[211] Sorenson E.S. (1914). The Tale of the Dingo. Sydney Mail.

[212] (1920). Story of a Dingo. The School Magazine of Literature for our Boys and Girls.

[213] Kernovake R. (1926). An Adventure with Dingoes. Maryborough Chronicle, Wide Bay and Burnett Advertiser.

[214] (1932). Brave Granny. Maryborough Chronicle, Wide Bay and Burnett Advertiser.

[215] (1870). Some of My Early Adventures. Australian Town and Country Journal.

[216] Hetherington C. (1906). Original Poetry: The Schooley’s Adventure. The Tumut Advocate and Farmers and Settlers’ Adviser.

[217] Winton J.M. (1923). The Lost Child. Transcontinental.

[218] Charnley W. (1946). The Dingo. Walkabout.

[219] (1865). Incidents of the Month. Geelong Advertiser.

[220] (1990). With Burke and Wills. Survivors’ Memories. The Telegraph.

[221] (1842). News from the Interior. The Sydney Morning Herald.

[222] (1859). Upper Castlereagh River. The Sydney Morning Herald.

[223] (1878). Our District Council. Northern Territory Times and Gazette.

[224] (1899). Obituary. The Late Alexander Mckenzie. The Queanbeyan Observer.

[225] Paterson A.B. (1905). The Old Bush Songs: Composed and Sung in the Bushranging, Digging, and Overlanding Days.

[226] Karskens G. (2010). The Colony: A History of Early Sydney.

[227] Rus R. (1844). Adventure in the Wollombi Mountains. The Weekly Register of Politics, Facts and General Literature.

[228] Thompson E.H. (1900). The Kennel. The Sydney Morning Herald.

[229] (1876). Colonial News. Maryborough Chronicle, Wide Bay and Burnett Advertiser.

[230] Storey W.K. (1991). Big Cats and imperialism: Lion and tiger hunting in Kenya and northern India, 1898–1930. J. World Hist..

[231] Wilson M. (1927). The Dingo. Sydney Mail.

[232] (1933). No title. The Bulletin.

[233] Possum J. (1928). No title. The Bulletin.

[234] Drover (1933). The Dingo: Its Fear of the Human Being. Sydney Mail.

[235] (1928). Me Bit ‘er Skirt. The Capricornian.

[236] (1938). Man-Killers Rare in Australian Bush. Sunday Mail.

[237] Domino (1928). Dingoes and Their Destruction. Great Southern Leader.

[238] (1924). Fear of Dingoes. Advising Migrants. Humour and Pathos. The Daily Mail.

[239] (1936). No Fear of Dingoes. The Queenslander.

[240] Yasmar (1934). Random Notes and Observations. Dingoes and Dogs. The Wingham Chronicle and Manning River Observer.

[241] (1926). A Northern Woman. Dingoes. The World’s News.

[242] Gouger (1928). No title. The Bulletin.

[243] Idriess I.L. (1958). Back O’ Cairns: The Story of Gold Prospecting in the Far North.

[244] Meston R. (1852). On the Management and Diseases of Australian Sheep. No. 22. Wild Dogs. The People’s Advocate and New South Wales Vindicator.

[245] (1932). Ban on Alsatians. Maryborough Chronicle, Wide Bay and Burnett Advertiser.

[246] (1928). Alsatians Banned. Potential Danger. Crossing with Dingo. Maryborough Chronicle, Wide Bay and Burnett Advertiser.

[247] (1934). “Alsatian” Dingo?. Maryborough Chronicle, Wide Bay and Burnett Advertiser.

[248] (1933). Legislative Assembly Land Estimates. Maryborough Chronicle, Wide Bay and Burnett Advertiser.

[249] Iford (1933). The Menace. The Bulletin.

[250] Threadingham T. (2015). Wild Dog Trapper Becomes Prey in Patrick Estate Attack. The Gatton, Lockyer and Brisbane Valley Star.

[251] (1941). Dangerous Dingoes Will Not Attack Man. The Inverell Times.

[252] Sinclair J.G. (1948). Dingo Habits. Maryborough Chronicle.

[253] Sinclair J.C. (1946). The Dingo. Maryborough Chronicle, Wide Bay and Burnett Advertiser.

[254] (1946). Missing Man May be Dingo’s Victim. Newcastle Morning Herald and Miners’ Advocate.

[255] (1947). Around the Camp Fire. Townsville Daily Bulletin.

[256] McNeill A.T., Luke K.-P.L., Goullet M.S., Gentle M.N., Allen B.L. (2016). Dingoes at the doorstep: Home range sizes and activity patterns of dingoes and other wild dogs around urban areas of north-eastern Australia. Animals.

[257] Green M. Peri-urban wild dog GPS collar project: Case study. Proceedings of the 5th Queensland Pest Animal Symposium.

[258] Harriott L., Gentle M., Traub R., Soares-Magalhaes R., Cobbold R. *Echinococcus granulosus* and other zoonotic pathogens of periurban wild dogs in south-east Queensland. Proceedings of the 5th Queensland Pest Animal Symposium.

[259] Allen B.L., Allen L.R., Amos M., Carmelito E., Gentle M.N., Goullet M.S., Leung L.K.-P., McNeill A.T., Speed J. Peri-urban wild dogs: Diet and movements in north-eastern Australia. Proceedings of the 5th Queensland Pest Animal Symposium.

[260] Allen B.L., Carmelito E., Amos M., Goullet M.S., Allen L.R., Speed J., Gentle M., Leung L.K.P. (2016). Diet of dingoes and other wild dogs in peri-urban areas of north-eastern Australia. Sci. Rep..

[261] Cairns K.M., Nesbitt B.J., Laffan S.W., Letnic M., Crowther M.S. (2019). Geographic hot spots of dingo genetic ancestry in southeastern Australia despite hybridisation with domestic dogs. Conserv. Genet..

[262] Gentle M., Oakey J., Speed J., Allen B., Allen L. Dingoes, domestic dogs, or hybrids? Genetics of peri-urban wild dogs in NE Australia. Proceedings of the 5th Queensland Pest Animal Symposium.

[263] Gentle M., Allen B.L., Speed J. (2017). Peri-Urban Wild Dogs in North-Eastern Australia: Ecology, Impacts and Management.

[264] Pierce J. (2012). Packs of Wild Dogs Venturing in to Queensland Urban Areas. The Courier Mail.

[265] Klein F. (2017). Brazen Attack: What Will This Killer Take Next?. The Gympie Times.

[266] Crowley C. (2014). Safety of Kids and Pets in Doubt as Dingo Attacks Increase. Daily Mercury.

[267] Alp C. (2019). Beloved Family Pets ‘Torn to Pieces’. Whitsunday Times.

[268] Shepperson R. (2018). Wild Dog Pack Terrorise Sarina Residents. Daily Mercury.

[269] Elder K. (2019). Loved Pet Killed by Wild Dog Attack on Castle Hill. Townsville Bulletin.

[270] Martinelli P. (2020). Kewarra Beach Family Loses Pup in Wild Dog Attack. The Cairns Post.

[271] Glenday J. (2012). MP Warns of Dingo Dangers in Darwin Suburbs. ABC News.

[272] Weston P. (2014). Gold Coast Hinterland Farmers Arm Themselves against Wild Dogs, Sparking Concern the Measures Are Cruel. Gold Coast Bulletin.

[273] Arylko (2015). Dog victim: “I Was Screaming—I Thought I Was Going to Die”. Tweed Daily News.

[274] Crockford T. (2018). Young Boy ‘Mauled by Red-Coloured Dog’ on North Queensland Beach. Brisbane Times.

[275] (1805). A Trip to Botany. The Sydney Gazette and New South Wales Advertiser.

[276] (1815). Sydney. The Sydney Gazette and New South Wales Advertiser.

[277] (1815). Sydney. The Sydney Gazette and New South Wales Advertiser.

[278] (1841). A Child Lost. Sydney Free Press.

[279] (1843). Hunter River District News, [From Our Correspondents] Upper Hunter. The Maitland Mercury and Hunter River General Advertiser.

[280] (1844). Melbourne. Geelong Advertiser.

[281] Gale J. (1927). Canberra: History of and Legends Relating to the Federal Capital Territory of the Commonwealth of Australia.

[282] (1850). Hunter River District News. The Maitland Mercury and Hunter River General Advertiser.

[283] (1850). Bathurst. Quarter Sessions. The Sydney Morning Herald.

[284] (1851). Domestic Intelligence. The Argus.

[285] (1851). Country News. Bathurst Free Press.

[286] (1864). Local Notes and Events. Queanbeyan Age and General Advertiser.

[287] (1867). Miscellaneous Information. New South Wales Police Gazette and Weekly Record of Crime.

[288] (1870). Albany. The Inquirer and Commercial News.

[289] (1872). Colonial Reminiscences. Two Days at Pammamera. Australian Town and Country Journal.

[290] (1875). A Man Attacked by Native Dogs. Ovens and Murray Advertiser.

[291] (1876). Local and General. Maryborough Chronicle, Wide Bay and Burnett Advertiser.

[292] (1879). No title. The Brisbane Courier.

[293] (1883). The Affirmation Bill. South Australian Register.

[294] (1933). Tragedy of a Lost Child. Chronicle.

[295] (1884). Local News. Maryborough Chronicle, Wide Bay and Burnett Advertiser.

[296] (1934). Fifty Years Ago. Maryborough Chronicle, Wide Bay and Burnett Advertiser.

[297] (1887). Child Lost in the Scrub. South Australian Weekly Chronicle.

[298] Carson W.E. (1932). Do Dingoes Eat Men?. Chronicle.

[299] (1888). News of the Day. The Inquirer and Commercial News.

[300] (1889). Rural Topics and Events. The Australasian.

[301] (1899). Sensational Story of the Bush. Living on Nettles and Fern Roots. Treed by Dingoes. A woman’s Experience. Barrier Miner.

[302] (1901). Paradise. Maryborough Chronicle, Wide Bay and Burnett Advertiser.

[303] (1902). A Prospector’s Sad Death. Body Eaten by Dingoes. The Port Augusta Dispatch, Newcastle and Flinders Chronicle.

[304] Field J. (1902). Body of Harry Hemming found by the Native Trackers—Re.

[305] Idriess I.L. (1946). Man Tracks: With the Mounted Police in Australian Wilds.

[306] (1906). Sydney. Maryborough Chronicle, Wide Bay and Burnett Advertiser.

[307] (1909). Attacked by Dingoes. Miner’s Terrible Experience. Singleton Argus.

[308] (1912). Wild Dogs Supposed to Have Killed a Boy. The Brisbane Courier.

[309] (1916). Lost Child Found Dead. Attacked by Animals. The Telegraph.

[310] (1917). A Child’s Sad Fate. Warwick Examiner and Times.

[311] Herodot (1924). No title. The Bulletin.

[312] (1925). Surrounded by Dingoes. Maryborough Chronicle, Wide Bay and Burnett Advertiser.

[313] (1927). Dingoes Attack Youth. Manilla Express.

[314] (1927). Attacked by Dingoes. Nepean Times.

[315] (1928). Dingoes Attack Men. One Beast Is Killed in Early Morning Fight. Barrier Miner.

